# Supplementary material for: On the interplay between lipids and asymmetric dynamics of an NBS degenerate ABC transporter
Source: Commun Biol. 2023 Feb 3;6:149. doi: 10.1038/s42003-023-04537-3 (PMC9898250; doi:10.1038/s42003-023-04537-3)
Supplement: Supplementary file 2 — Supplementary Information [file 42003_2023_4537_MOESM2_ESM.pdf]

Supplementary information for

On the interplay between lipids and asymmetric dynamics of an NBS degenerate ABC transporter

Ágota Tóth, Angelika Janaszkiewicz, Veronica Crespi and Florent Di Meo*

Inserm U1248 Pharmacology & Transplantation, ΩHealth Institute – Univ. Limoges, 2 rue du Prof. Descottes, 87000 F-Limoges, France

Contact information

Dr. Florent Di Meo

Inserm U1248 Pharmacology & Transplantation, ΩHealth Institute - Univ. Limoges,

2 rue du Prof. Descottes,

87000 F-Limoges, France

florent.di-meo@inserm.fr

Tel: +33(0)5 19 56 42 76

Table of contents

List of Supplementary Tables 2
List of Supplementary Figures 3
Supplementary Tables 6
Supplementary Figures 33
Supplementary Movies 83

List of Supplementary Tables

Supplementary Table 1. Residues selected for the calculations of ABC structural parameters of ABC transporters used for mapping the ABC conformational space, namely a) EC and IC angles and b) NBD distance and twist. Selected ABC transporters are reported according to their PBD ID. Residues were selected as explained in the Method section........ 6

Supplementary Table 2. EC angles (°) calculated from each MD simulation replica performed for the different *b*MRP1 systems in different lipid bilayer membranes considered in the present study. Standard deviations are reported in brackets. 11

Supplementary Table 3. IC angles (°) calculated from each MD simulation replica performed for the different *b*MRP1 systems in different lipid bilayer membranes considered in the present study. Standard deviations are reported in brackets. 12

Supplementary Table 4. NBD distances (Å) calculated from each MD simulation replica performed for the different *b*MRP1 systems in different lipid bilayer membranes considered in the present study. Standard deviations are reported in brackets. 13

Supplementary Table 5. NBD twists (°) calculated from each MD simulation replica performed for the different *b*MRP1 systems in different lipid bilayer membranes considered in the present study. Standard deviations are reported in brackets. 14

Supplementary Table 6. Overlaps of calculated network communities between the different IF systems and OF *b*MRP1-$(ATP)_2$. Analyses were performed in POPC:POPE:Chol (2:1:1) lipid bilayer. For a given community, overlaps were obtained by calculating the proportion of shared residues between target system and OF *b*MRP1-$(ATP)_2$........ 15

Supplementary Table 7. The number of calculated raw NBD communities obtained from MD simulations performed in POPC:POPE:Chol (2:1:1). It is worth mentioning that for IF apo *b*MRP1 and OF *bMRP1*-$(ATP)_2$, more than 2 communities per NBD were sometimes observed. They were however systematically corresponding to subcommunities of either Walker A or signature community described in Supplementary Table 6. 16

Supplementary Table 8. Key distances between ATP and *b*MRP1 NBD or IC-loop for a) OF *b*MRP1-$(ATP)_2$, b) *b*MRP1-LTX-$(ATP)_2$ and c) *b*MRP1-$(ATP)_2$. Values were averaged over each replica; standard deviations are reported in brackets. 17

Supplementary Table 9. Selected residues for allosteric pathway calculations from substrate-binding site to NBS1 and NBS2........ 21

Supplementary Table 10. Membrane free energy deformations. Bilayer Thicknesses at the outer edge of the simulation cells in POPC:POPE:Chol (2:1:1). 22

Supplementary Table 11. Membrane free energy deformations. Bilayer Thicknesses at the outer edge of the simulation cells in POPC:Chol (3:1)........ 23

Supplementary Table 12. Membrane free energy deformations. Bilayer Thicknesses at the outer edge of the simulation cells in pure POPC. 24

Supplementary Table 13. L_0 modelling of OF state........ 25

Supplementary Table 14. Box sizes (Å) of each system considered in the present study. 26

Supplementary Table 15. Distances used to originally restrain Mg2+-ATP-NBD arrangement in MD simulations. Distances were measured on the minimized structure (OF *b*MRP1-$(ATP)_2$) and on the starting structure (*b*MRP1-$(ATP)_2$ and *b*MRP1-LTX-$(ATP)_2$) 28

Supplementary Table 16. Force constants (in kcal.mol^{-1}.Å^{-2}) used to restrain Mg2+-ATP-NBD arrangement. 30

Supplementary Table 17. Total length of the MD simulations for each replica in nanoseconds. In total, 112.37 µs MD simulations were conducted in the present study. 31

List of Supplementary Figures

Supplementary Figure 1. Distribution of ABC structural parameters obtained in different lipid bilayer compositions. a) POPC:POPE:Chol (2:1:1) b) POPC:Chol (3:1) and c) POPC. 33

Supplementary Figure 2. Evolution of EC angle (°) along MD simulations calculated for all the systems and for all different membrane compositions. Replica 1, 2 and 3 are coloured red, blue and yellow, respectively. 34

Supplementary Figure 3. IC angle (°) during the whole simulations calculated for all the systems and for all different membrane compositions. Replica 1, 2 and 3 are coloured red, blue and yellow, respectively. 35

Supplementary Figure 4. NBD distance (Å) during the whole simulations calculated for all the systems and for all different membrane compositions. Replica 1, 2 and 3 are coloured red, blue and yellow, respectively. 36

Supplementary Figure 5. NBD twist angle (°) during the whole simulations calculated for all the systems and for all different membrane compositions. Replica 1, 2 and 3 are coloured red, blue and yellow, respectively. 37

Supplementary Figure 6. EC distance (Å) during the whole simulations calculated for all the systems and for all different membrane compositions. Replica 1, 2 and 3 are coloured red, blue and yellow, respectively. 38

Supplementary Figure 7. Gly681-Ser1430 ($dGSNBS1$) distances (Å) during the whole simulations calculated for all the systems and for all different membrane compositions. Replica 1, 2 and 3 are coloured red, blue and yellow, respectively. 39

Supplementary Figure 8. Ser769-Gly1329 ($dGSNBS2$) distances (Å) during the whole simulations calculated for all the systems and for all different membrane compositions. Replica 1, 2 and 3 are coloured red, blue and yellow, respectively. 40

Supplementary Figure 9. Calculated z-dependent pore radii for all systems investigated in the present study. Replica 1, 2 and 3 are coloured red, blue and yellow, respectively. 41

Supplementary Figure 10. Time-dependent pore radius profiles at z = 18 Å. a) IF apo *b*MRP1, b) *b*MRP1-$(ATP)_2$, c) *b*MRP1-LTX, d) *b*MRP1-LTX-$(ATP)_2$ and e) OF *b*MRP1-$(ATP)_2$. 42

Supplementary Figure 11. Time-dependent pore radius profiles at z = 5 Å. a) IF apo *b*MRP1, b) *b*MRP1-$(ATP)_2$, c) *b*MRP1-LTX, d) *b*MRP1-LTX-$(ATP)_2$ and e) OF *b*MRP1-$(ATP)_2$. 43

Supplementary Figure 12. Time-dependent pore radius profiles at z = -15 Å. a) IF apo *b*MRP1, b) *b*MRP1-$(ATP)_2$, c) *b*MRP1-LTX, d) *b*MRP1-LTX-$(ATP)_2$ and e) OF *b*MRP1-$(ATP)_2$. 44

Supplementary Figure 13. Time-dependent pore radius profiles at z = -22 Å. a) IF apo *b*MRP1, b) *b*MRP1-$(ATP)_2$, c) *b*MRP1-LTX, d) *b*MRP1-LTX-$(ATP)_2$ and e) OF *b*MRP1-$(ATP)_2$. 45

Supplementary Figure 14. Per-residue averaged Root-mean-square fluctuations (RMSF), obtained after equilibration of the different systems in different lipid bilayer composition. 46

Supplementary Figure 15. Principal component analysis (PCA) of the different systems. For a given system, trajectories were aligned to an average structure obtained from the whole set of simulations. a) Arrows show the main movements in PC1-PC3, while on panel b) the variability of 10 PCs is shown. 47

Supplementary Figure 16. Residue contributions to the three first components. a) PC1, b) PC2 and c) PC3 highlighting the major contribution of NBD2. 48

Supplementary Figure 17. Contact maps between NBDs and Coupling Helices (CHs) obtained from MD simulations performed in POPC:POPE:Chol (2:1:1). a) IF apo *b*MRP1, b) *b*MRP1-(ATP)$_2$, c) *b*MRP1-LTX, d) *b*MRP1-LTX-(ATP)$_2$ and e) OF *b*MRP1-(ATP)$_2$. 49

Supplementary Figure 18. Contact maps between Coupling Helices (CHs) obtained from MD simulations performed in POPC:POPE:Chol (2:1:1). a) IF apo *b*MRP1, b) *b*MRP1-(ATP)$_2$, c) *b*MRP1-LTX, d) *b*MRP1-LTX-(ATP)$_2$ and e) OF *b*MRP1-(ATP)$_2$. 50

Supplementary Figure 19. Contact maps between NBDs obtained from MD simulations performed in POPC:POPE:Chol (2:1:1). a) IF apo *b*MRP1, b) *b*MRP1-(ATP)$_2$, c) *b*MRP1-LTX, d) *b*MRP1-LTX-(ATP)$_2$ and e) OF *b*MRP1-(ATP)$_2$ 51

Supplementary Figure 20. Overall dynamic cross-correlation matrices from MD simulations performed POPC:POPE:Chol (2:1:1). a) IF apo *b*MRP1, b) *b*MRP1-(ATP)$_2$, c) *b*MRP1-LTX, d) *b*MRP1-LTX-(ATP)$_2$ and e) OF *b*MRP1-(ATP)$_2$ 52

Supplementary Figure 21. Representative NBD communities from Network Analyses calculated from MD simulations performed in POPC:POPE:Chol (2:1:1) simulations. a) IF apo *b*MRP1, b) *b*MRP1-(ATP)$_2$, c) *b*MRP1-LTX, d) *b*MRP1-LTX-(ATP)$_2$ and e) OF *b*MRP1-(ATP)$_2$. NBD1 and NBD2 are coloured yellow and cyan, respectively. Orange and blue colours show the TMH parts involved in the communities. The Walker A community is purple, while the signature sequence community silver. NBD dimers are shown from upper view. 53

Supplementary Figure 22. Non-covalent interaction energies between LTX and *b*MRP1 extracted from Coulomb (top) and van der Waals (bottom) potentials obtained from MD simulations performed in a) POPC:POPE:Chol (2:1:1), b) POPC:Chol (3:1) and c) POPC lipid bilayers. 54

Supplementary Figure 23. Calculated per-residue betweennesses in the allosteric pathway from substrate binding pocket to NBS1 (left) and NBS2 (right). a) IF apo *b*MRP1, b) *b*MRP1-(ATP)$_2$, c) *b*MRP1-LTX, d) *b*MRP1-LTX-(ATP)$_2$ and e) OF *b*MRP1-(ATP)$_2$ systems embedded in POPC. 55

Supplementary Figure 24. Calculated per-residue betweennesses in the allosteric pathway from substrate binding pocket to NBS1 (left) and NBS2 (right). a) IF apo *b*MRP1, b) *b*MRP1-(ATP)$_2$, c) *b*MRP1-LTX, d) *b*MRP1-LTX-(ATP)$_2$ and e) OF *b*MRP1-(ATP)$_2$ systems embedded in POPC:Chol (3:1). 56

Supplementary Figure 25. Calculated per-residue betweennesses in the allosteric pathway from substrate binding pocket to NBS1 (left) and NBS2 (right). a) IF apo *b*MRP1, b) *b*MRP1-(ATP)$_2$, c) *b*MRP1-LTX, d) *b*MRP1-LTX-(ATP)$_2$ and e) OF *b*MRP1-(ATP)$_2$ systems embedded in POPC:POPE:Chol (2:1:1). 57

Supplementary Figure 26. Average normalized information flow through *b*MRP1 protein regarding the allosteric communication from substrate binding pocket to NBS1 and NBS2 for IF apo *b*MRP1 and *b*MRP1-LTX system in POPC:POPE:Chol (2:1:1). 58

Supplementary Figure 27. ABC conformational space of trajectories in POPC compared to resolved ABC transporters. 59

Supplementary Figure 28. ABC conformational space of trajectories in POPC:Chol (3:1) compared to resolved ABC transporters. 60

Supplementary Figure 29. Free energy landscapes according to NBD distance and NBD twist. obtained in different lipid bilayers using the InfleCS approach for IF systems, embedded in different lipid bilayers. a) IF apo *b*MRP1, b) *b*MRP1-(ATP)$_2$, c) *b*MRP1-LTX, d) *b*MRP1-LTX-(ATP)$_2$ and e) OF *b*MRP1-(ATP)$_2$...61

Supplementary Figure 30. Free energy landscapes according to NBD twist and EC angle obtained in different lipid bilayers using the InfleCS approach for OF *b*MRP1-(ATP)$_2$ systems. 62

Supplementary Figure 31. Tilt angles with respect to membrane normal axis of different transmembrane helices. Since TMD$_0$ was not modelled in the present study, the conventional TMH1 to TMH12 labelling for type I ABC transporter was used. 63

Supplementary Figure 32. Tilt angles of bundles A, B, C and D which consist in (TMH1, 2, 10 and 11), (TMH4, 5, 7 and 8), (TMH 3 and 6) and (TMH9 and 12), respectively (see main text). 64

Supplementary Figure 33. Calculated binding hotspots obtained from cholesterol defined by presence likelihood higher than 50%. Cryo-EM resolved cholesterols are coloured violet, other cholesterols green. 65

Supplementary Figure 34. PE-lipid distribution in POPC:POPE:Chol (2:1:1) lipid bilayer. 66

Supplementary Figure 35. Cholesterol distribution in POPC:POPE:Chol (2:1:1) lipid bilayer. 67

Supplementary Figure 36. Cholesterol distribution in POPC:Chol (3:1) lipid bilayer. 68

Supplementary Figure 37. Putative substrate access to *b*MRP1 binding pocket. We here propose different possible substrate access to *b*MRP1 binding pocket depending on substrate lipid bilayer partitioning. Amphiphilic compounds including charged molecules were shown to possibly partition within the high-density polar head region of lipid bilayer membrane. Our MD simulations suggest that access to TMD binding pocket may be possible between TMH4 and TMH6 while access between TMH10 and TMH12 might less likely. 69

Supplementary Figure 38. Root-mean square fluctuation of L_0. The part which was modelled using the sequence and not the loop from the IF model (around 80-95) is the most flexible part in the IF models, as well. 70

Supplementary Figure 39. L_0 structures. a) Starting frames converge to the same conformation shown by b) the final frames. Top view., aligned on TH1-3,6,10-11 (bundle A and C). IF apo is blue, IF ATP-bound ochre, IF LTX-bound yellow, IF LTX-ATP-bound green, and OF ATP-bound mauve/pink. 71

Supplementary Figure 40. Root-mean-square deviation (RMSD) along MD simulations calculated for all the systems and for all different membrane compositions. Replica 1, 2 and 3 are coloured red, blue and yellow, respectively. 72

Supplementary Figure 41. Convergence of InfleCS calculations in POPC:POPE:Chol (2:1:1). a) IF apo *b*MRP1, b) *b*MRP1-$(ATP)_2$, c) *b*MRP1-LTX, d) *b*MRP1-LTX-$(ATP)_2$ and e) OF *b*MRP1-$(ATP)_2$...............73

Supplementary Figure 42. Convergence of InfleCS calculations in POPC:Chol (3:1). a) IF apo *b*MRP1, b) *b*MRP1-$(ATP)_2$, c) *b*MRP1-LTX, d) *b*MRP1-LTX-$(ATP)_2$ and e) OF *b*MRP1-$(ATP)_2$. 74

Supplementary Figure 43. Convergence of InfleCS calculations in pure POPC. a) IF apo *b*MRP1, b) *b*MRP1-$(ATP)_2$, c) *b*MRP1-LTX, d) *b*MRP1-LTX-$(ATP)_2$ and e) OF *b*MRP1-$(ATP)_2$. 75

Supplementary Figure 44. Atom selections for co-factor nodes for allosteric pathway calculations. Each cofactor as split into fragments (depicted in blue, green, red and pink shades, accordingly) that were used to defined nodes for allosteric pathway calculations. PC and PE lipids were split into three nodes corresponding to polar head and lipid tails. Cholesterol and Mg^{2+} were considered as single node each. ATP molecule was split into three fragments (purine and sugar moieties as well as phosphate tail). LTX was split into three fragments (namely, glutathione moiety, aliphatic chain and 6-ketohexanoate moiety). 76

Supplementary Figure 45. Number of H-bonds calculated between nucleotides and *b*MRP1 protein over MD simulations. H-bond interactions were counted between nucleotides and protein residues over MD simulations performed on *b*MRP1-$(ATP)_2$, *b*MRP1-LTX-$(ATP)_2$ and OF *b*MRP1-$(ATP)_2$ systems embedded in a) POPC:POPE:Chol (2:1:1), b) POPC:Chol (3:1) and c) POPC. Distance and angle cutoffs were set 3.5 Å and 120°, respectively. 77

Supplementary Figure 46. Number of H-bonds calculated between LTX and *b*MRP1 protein over MD simulations. H-bond interactions were counted between LTX and proteins over MD simulations performed on *b*MRP1-LTX and *b*MRP1-LTX-$(ATP)_2$ systems embedded in a) POPC:POPE:Chol (2:1:1), b) POPC:Chol (3:1) and c) POPC. Distance and angle cutoffs were set 3.5 Å and 120°, respectively. 78

Supplementary Figure 47. Overall dynamic cross-correlation matrices from MD simulations performed in POPC. a) IF apo *b*MRP1, b) *b*MRP1-$(ATP)_2$, c) *b*MRP1-LTX, d) *b*MRP1-LTX-$(ATP)_2$ and e) OF *b*MRP1-$(ATP)_2$. 79

Supplementary Figure 48. Overall dynamic cross-correlation matrices from MD simulations performed in POPC:Chol (3:1). a) IF apo *b*MRP1, b) *b*MRP1-$(ATP)_2$, c) *b*MRP1-LTX, d) *b*MRP1-LTX-$(ATP)_2$ and e) OF *b*MRP1-$(ATP)_2$..80

Supplementary Figure 49. Overall dynamic cross-correlation matrices from MD simulations performed in POPC:POPE (3:1). a) IF apo *b*MRP1 and b) OF *b*MRP1-$(ATP)_2$. 81

Supplementary Figure 50. Overall dynamic cross-correlation matrices from MD simulations performed in POPE. a) IF apo *b*MRP1 and b) OF *b*MRP1-$(ATP)_2$. 82

Supplementary Tables

Supplementary Table 1. Residues selected for the calculations of ABC structural parameters of ABC transporters used for mapping the ABC conformational space, namely a) EC and IC angles and b) NBD distance and twist. Selected ABC transporters are reported according to their PBD ID. Residues were selected as explained in the Methods section. ABCC1/MRP1 PDB IDs are shown as bold.

a) EC and IC angles

PDB ID	EC angle		IC angle	
	**Vector1 direction**	**Vector2 direction**	**Vector1 direction**	**Vector2 direction**
4PL0	Chain A 273-274,285-286<br>Chain B 48-49,64-67	Chain A 47-52,60-67<br>Chain B 273-275,284-286	Chain A 24-25,90-109,125-141,309-323<br>Chain B 188-212,229-250	Chain A 188-213,228-250<br>Chain B 21-25,90-109,124-141,308-322
5TTP	Chain A 161,167,270-273,284-287<br>Chain B 47-52,63-67	Chain A 46-52,61-67<br>Chain B 270-272,283-286	Chain A 91-110,123-138,309-322<br>Chain B 190-211,226-244	Chain A 190-211,227-245<br>Chain B 90-108,123-139,309-321
4RY2	Chain A 412<br>Chain B 192-194,204-206	Chain A 192-195,203-206<br>Chain B 412,423	Chain A 232-252,265-282,447-462<br>Chain B 327-351,366-388	Chain A 327-352,366-388<br>Chain B 232-249,264-282,447-461
4S0F		Chain A 194-195,203-206<br>Chain B 412,423	Chain A 231-252,265-284,447-462<br>Chain B 327-351,366-388	
5C73	Chain C 278<br>Chain K 42-43,72	Chain C 40-44,69-74<br>Chain K 277-278,286-287	Chain C 16-17,98-118,131-148,311-327<br>Chain K 195-218,232-253	Chain A 195-218,232-255<br>Chain B 15-17,96-117,131-148,311-324
2ONJ	Chain A 265-267,279-282<br>Chain B 37-42,58-62	Chain A 38-43,56-59<br>Chain B 267,278-282	Chain A 15,83,86-105,120-135,304-317<br>Chain B 182-205,221-244	Chain A 183-208,221-242<br>Chain B 86-103,119-135,303-316
4Q4A	Chain A 256-259,271-272<br>Chain B 61-65,76-79	Chain A 37-42,49-56<br>Chain B 280-283,294-296	Chain A 78-98,111-129,294-309	Chain A 174-200,213-235<br>Chain B 100-121,135-155,316-332

			Chain B 199-223,237-258,260-260	
5MKK	Chain A 276-280,292-293<br>Chain B 47-50,58-61	Chain A 51-55,67-76<br>Chain B 264-267,276-277	Chain A 99-116,133-151,314-330<br>Chain B 181-204,221-240,242	Chain A 197-222,234-254<br>Chain B 22-24,83-103,117-137,297-314
6RAF	Chain A 277-280,292-293<br>Chain B 46-50,58-61	Chain A 51-55,67-76<br>Chain B 263-267,276-278	Chain A 98-116,133-152,314-330<br>Chain B 181-204,221-239	Chain A 196-222,234-255<br>Chain B 23,84-103,117-136,300-314
6RAG	Chain A 277-280,292-293<br>Chain B 47-50,58-61	Chain A 51-55,67-76<br>Chain B 264-267,276-277	Chain A 98-116,133-152,314-330<br>Chain B 180-204,221-242	Chain A 196-222,234-255<br>Chain B 22-24,83-103,117-137,297-314
6RAH	Chain A 279-280,292-295<br>Chain B 46-50,58	Chain A 48,50-55,67-75<br>Chain B 262-267,276-280	Chain A 99-116,133-149,315-330<br>Chain B 181-204,221-240	Chain A 195-222,234-256<br>Chain B 23,84-103,117-134,301-314
6RAI	Chain A 279-280,292<br>Chain B 46-50,58-60	Chain A 52-55,67-74<br>Chain B 264-267,276-277	Chain A 27-29,96-116,133-153,313-330<br>Chain B 178-204,221-243	Chain A 195-222,234-257<br>Chain B 22-24,81-103,117-137,297-297,299-314
6RAJ	Chain A 276-280,292-295<br>Chain B 46-50,58-61	Chain A 48,50-55,67-74<br>Chain B 263-267,276-280	Chain A 99-116,133-149,316-330<br>Chain B 181-204,221-240	Chain A 195-222,234-255<br>Chain B 84-103,117-134,301-314
6RAK	Chain A 279-280,292<br>Chain B 46-50,58-60	Chain A 52-55,67-75<br>Chain B 265-267,276-277	Chain A 27-29,96-116,133-152,313-330<br>Chain B 178-204,221-243	Chain A 195-222,234-257<br>Chain B 22-24,81-103,117-137,299-314
6RAL	Chain A 276-280,292-294<br>Chain B 46-50,58-61	Chain A 51-55,67-76<br>Chain B 263-267,276-278	Chain A 99-116,133-150,315-330<br>Chain B 180-204,221-241	Chain A 196-222,234-255<br>Chain B 22-23,84-103,117-134,136-136,300-314
6RAM	Chain A 277-280,292-293<br>Chain B 46-50,58-61	Chain A 51-55,67-75<br>Chain B 263-267,276-278	Chain A 27-29,96-116,133-152,313-330<br>Chain B 180-204,221-242	Chain A 195-222,234-257<br>Chain B 22-23,82-103,117-136,299-314
6RAN	Chain A 277-280,292-293<br>Chain B 46-50,58-61	Chain A 51-55,67-76<br>Chain B 262-267,276-278	Chain A 27-28,98-116,133-152,312-330<br>Chain B 180-204,221-240,242	Chain A 195-222,234-257<br>Chain B 22-24,82-103,117-137,299-314

**5UJ9**	Chain A 568-569,581,996-999,1011-1017	Chain A 348-352,360-363,1217-1218,1226-1228	Chain A 324-325,386-409,422-439,441,603-619,1130-1158,1172-1195	Chain A 484-510,523-542,1039-1056,1070-1089,1250-1264
**5UJA**	Chain A 460,565-569,581-582,995-999,1011-1017	Chain A 348-352,360-363,1226-1227	Chain A 324-325,386-409,422-439,605-619,1130-1158,1172-1195,1197-1197	Chain A 487-510,523-540,1039-1056,1070-1087,1089-1089,1248-1248,1250-1264
**6BHU**	Chain A 460,565-569,581-585,995-999,1011-1016	Chain A 348-352,360-363,1218,1226-1230	Chain A 324-325,386-409,422-438,605-619,1130-1158,1172-1195	Chain A 487-510,523-542,971,1037-1056,1070-1087,1251-1264
**6UY0**	Chain A 460,565-569,581-584,996-999,1011-1015	Chain A 348,350-352,360-363,1218,1226-1230	Chain A 324-325,386-409,422-438,605-619,1131-1158,1172-1195	Chain A 487-510,523-542,1038-1056,1070-1087,1251-1264
6LR0	Chain U 342-345,358-359,778-781,793-798	Chain U 84-89,135-141,1001-1006,1015-1017	Chain U 164-184,197-214,381-396,920-943,960-977	Chain U 261-286,299-319,820-838,856-875,1037-1053
6S7P	Chain A 319-321,332-334,733-737,748-752	Chain A 77-80,109-116,957-960,971-974	Chain A 52-53,140-158,173-188,358-370,877-900,914-933	Chain A 236-261,275-294,777-793,812-829,996-1009
6QEX	Chain A 317-318,330-330,736-737,751-753	Chain A 74-82,104-114,961-962,971-972	Chain A 137-153,171-187,353-368,880-900,916-935	Chain A 235,237-259,272-295,709-712,775-796,813-833,995-1009
6C0V	Chain A 209-209,314-318,330-334,732-737,751-755	Chain A 70-78,107-116,957-962,971-976	Chain A 139-156,171-185,356-368,878-900,916-934	Chain A 235-259,272-292,779-796,813-830,997-1009

b) NBD distance and NBD twist angle

**PDB ID**	**NBD distance**		**NBD twist angle**			
	NBD1	***NBD2***	***Vector1 origin***	***Vector1 direction***	***Vector2 origin***	***Vector2 direction***
4PL0	Chain A 344-554	Chain B 345-559	Chain A 344-425,500-506,530-554	Chain A 432-497,512-527	Chain B 345-425,500-506,530-559	Chain B 432-497,512-527
5TTP	Chain A 341-553	Chain B 342-556	Chain A 341-423,500-	Chain A 432-497,512-527	Chain B 342-423,500-505,530-556	Chain B 432-497,512-527

			505,530-553			
4RY2<br>4S0F	Chain A 485-695	Chain B 486-700	Chain A 485-566,643-648,673-695	Chain A 573-640,654-669	Chain B 486-566,643-647,673-700	Chain B 573-640,654-669
5C73	Chain C 348-558	Chain K 348-562	Chain C 348-429,505-511,535-558	Chain C 434-501,517-532	Chain K 348-429,505-511,535-562	Chain K 434-501,517-532
2ONJ	Chain A 339-550	Chain B 340-554	Chain A 339-421,498-503,528-550	Chain A 427-495,509-525	Chain B 340-421,498-503,528-554	Chain B 427-495,509-525
4Q4A	Chain A 331-541	Chain B 355-569	Chain A 331-413,490-495,520-541	Chain A 419-487,501-515	Chain B 355-435,512-516,542-569	Chain B 440-509,523-539
5MKK<br>6RAF<br>6RAG<br>6RAH<br>6RAI<br>6RAJ<br>6RAK<br>6RAL<br>6RAM<br>6RAN	Chain A 352-569	Chain B 339-553	Chain A 352-440,518-523,548-569	Chain A 447-514,530-545	Chain B 339-418,495-500,525-553	Chain B 422-492,507-520
**5UJ9**<br>**5UJA**<br>**6BHU**<br>**6UY0**	Chain A 643-842	Chain A 1291-1505	Chain A 643-711,788-791,821-850	Chain A 722-784,800-810	Chain A 1290-1372,1449-1453,1479-1507	Chain A 1381-1445,1461-1474
6LR0	Chain U 419-630	Chain U 1080-1294	Chain U 419-502,578-584,609-630	Chain U 506-574,591-605	Chain U 1080-1160,1239-1243,1269-1294	Chain U 1166-1235,1252-1263
6S7P	Chain A 393-605	Chain A 1034-1248	Chain A 393-476,553-558,583-612	Chain A 481-549,565-579	Chain A 1034-1116,1195-1200,1225-1248	Chain A 1121-1191,1207-1222
6QEX	Chain A 391-602	Chain A 1035-1249	Chain A 391-474,551-556,581-602	Chain A 479-547,563-576	Chain A 1035-1116,1196-1201,1226-1248	Chain A 1121-1191,1208-1222

6C0V	Chain A 392-602	Chain A 1035-1249	Chain A 392-473,551-556,581-602	Chain A 480-547,563-577	Chain A 1035-1117,1196-1201,1225-1249	Chain A 1123-1193,1208-1221

Supplementary Table 2. EC angles (°) calculated from each MD simulation replica performed for the different *b*MRP1 systems in different lipid bilayer membranes considered in the present study. Standard deviations are reported in brackets.

		IF apo *b*MRP1	*b*MRP1-$(ATP)_2$	*b*MRP1-LTX	*b*MRP1-LTX-$(ATP)_2$	OF *b*MRP1-$(ATP)_2$
POPC	*rep1*	13.43 (0.26)	13.71 (0.28)	14.10 (0.27)	14.10 (0.27)	13.93 (0.66)
	rep2	13.54 (0.31)	13.90 (0.36)	13.54 (0.29)	13.54 (0.29)	20.58 (1.57)
	rep3	13.37 (0.31)	13.12 (0.37)	13.33 (0.29)	13.33 (0.29)	16.98 (1.43)
POPE	*rep1*	13.34 (0.24)				17.53 (0.89)
	rep2	13.65 (0.28)	-	-	-	15.66 (0.52)
	rep3	13.32 (0.24)				16.74 (1.79)
POPC:POPE (3:1)	*rep1*	13.31 (0.33)				17.41 (2.46)
	rep2	13.48 (0.40)	-	-	-	13.32 (0.46)
	rep3	13.70 (0.41)				13.57 (0.34)
POPC:Chol (3:1)	*rep1*	13.52 (0.27)	13.44 (0.29)	13.33 (0.27)	13.33 (0.27)	18.87 (2.54)
	rep2	13.47 (0.28)	13.22 (0.24)	13.48 (0.3)	13.48 (0.3)	14.5 (0.79)
	rep3	13.69 (0.27)	13.12 (0.28)	13.34 (0.26)	13.34 (0.26)	14.63 (1.00)
POPC:POPE:Chol (2:1:1)	*rep1*	13.31 (0.26)	13.34 (0.25)	13.28 (0.25)	13.28 (0.25)	18.92 (1.42)
	rep2	13.55 (0.29)	13.36 (0.25)	13.47 (0.24)	13.47 (0.24)	18.67 (0.96)
	rep3	13.58 (0.34)	13.46 (0.23)	13.41 (0.26)	13.41 (0.26)	16.28 (0.86)

Supplementary Table 3. IC angles (°) calculated from each MD simulation replica performed for the different *b*MRP1 systems in different lipid bilayer membranes considered in the present study. Standard deviations are reported in brackets.

		IF apo *b*MRP1	*b*MRP1-$(ATP)_2$	*b*MRP1-LTX	*b*MRP1-LTX-$(ATP)_2$	OF *b*MRP1-$(ATP)_2$
POPC	*rep1*	31.53 (2.60)	35.37 (1.77)	29.92 (2.86)	29.92 (2.86)	20.77 (0.31)
	rep2	33.22 (2.04)	33.86 (1.00)	27.58 (0.55)	27.58 (0.55)	20.79 (0.21)
	rep3	28.69 (2.56)	26.89 (1.29)	26.43 (0.61)	26.43 (0.61)	21.06 (0.24)
POPE	*rep1*	27.77 (0.91)				20.69 (0.28)
	rep2	28.38 (2.49)	-	-	-	20.56 (0.63)
	rep3	28.88 (1.22)				20.9 (0.42)
POPC:POPE (3:1)	*rep1*	26.94 (0.96)				20.64 (0.43)
	rep2	27.76 (2.27)	-	-	-	20.74 (0.29)
	rep3	27.85 (2.35)				21.09 (0.42)
POPC:Chol (3:1)	*rep1*	28.63 (1.57)	28.75 (1.70)	26.04 (0.53)	26.04 (0.53)	20.42 (0.25)
	rep2	28.36 (1.48)	27.15 (1.55)	26.78 (0.61)	26.78 (0.61)	20.64 (0.31)
	rep3	26.28 (1.62)	26.45 (1.59)	26.46 (0.44)	26.46 (0.44)	20.82 (0.21)
POPC:POPE:Chol (2:1:1)	*rep1*	27.69 (1.12)	27.50 (1.1)	27.60 (1.03)	27.60 (1.03)	21.51 (0.37)
	rep2	26.64 (1.66)	27.34 (1.22)	26.05 (0.73)	26.05 (0.73)	20.92 (0.24)
	rep3	31.12 (3.90)	30.98 (1.02)	26.25 (0.54)	26.25 (0.54)	20.75 (0.34)

Supplementary Table 4. NBD distances (Å) calculated from each MD simulation replica performed for the different *b*MRP1 systems in different lipid bilayer membranes considered in the present study. Standard deviations are reported in brackets.

		**IF apo *b*MRP1**	***b*MRP1-(ATP)$_2$**	***b*MRP1-LTX**	***b*MRP1-LTX-(ATP)$_2$**	**OF *b*MRP1-(ATP)$_2$**
POPC	*rep1*	39.04 (7.33)	51.80 (4.80)	43.52 (9.74)	43.52 (9.74)	27.05 (0.21)
	rep2	52.62 (6.75)	55.01 (2.83)	33.89 (1.04)	33.89 (1.04)	27.46 (0.41)
	rep3	34.42 (7.64)	33.25 (3.10)	32.13 (2.35)	32.13 (2.35)	27.25 (0.21)
POPE	*rep1*	31.92 (3.01)				27.12 (0.19)
	rep2	34.11 (7.18)	-	-	-	27.29 (0.18)
	rep3	34.19 (4.84)				27.29 (0.21)
POPC:POPE (3:1)	*rep1*	30.36 (2.63)				27.27 (0.16)
	rep2	32.81 (5.55)	-	-	-	27.11 (0.18)
	rep3	33.64 (6.71)				27.38 (0.19)
POPC:Chol (3:1)	*rep1*	31.68 (4.25)	33.48 (6.34)	28.93 (1.46)	28.93 (1.46)	27.59 (0.25)
	rep2	33.30 (4.12)	33.61 (4.11)	31.55 (1.86)	31.55 (1.86)	27.57 (0.36)
	rep3	35.82 (3.99)	32.05 (4.99)	31.09 (0.57)	31.09 (0.57)	27.88 (0.27)
POPC:POPE:Chol (2:1:1)	*rep1*	32.68 (4.48)	31.33 (2.74)	34.52 (3.36)	34.52 (3.36)	27.33 (0.21)
	rep2	34.91 (5.51)	31.48 (4.49)	30.25 (1.92)	30.25 (1.92)	27.36 (0.22)
	rep3	40.33 (10.09)	36.97 (1.52)	30.56 (1.6)	30.56 (1.60)	27.06 (0.17)

Supplementary Table 5. NBD twists (°) calculated from each MD simulation replica performed for the different *b*MRP1 systems in different lipid bilayer membranes considered in the present study. Standard deviations are reported in brackets.

		**IF apo *b*MRP1**	***b*MRP1-(ATP)$_2$**	***b*MRP1-LTX**	***b*MRP1-LTX-(ATP)$_2$**	**OF *b*MRP1-(ATP)$_2$**
POPC	*rep1*	-141.82 (5.55)	-156.78 (26.72)	-151.58 (8.55)	-151.58 (8.55)	-143.28 (1.88)
	rep2	-140.39 (8.67)	-150.38 (44.18)	-126.67 (6.09)	-126.67 (6.09)	-143.66 (2.26)
	rep3	-147.39 (5.87)	-145.18 (32.55)	-140.15 (4.47)	-140.15 (4.47)	-144.72 (1.93)
POPE	*rep1*	-126.04 (5.12)	-	-	-	-143.67 (1.65)
	rep2	-136.34 (7.63)				-143.58 (1.39)
	rep3	-143.84 (5.92)				-143.15 (1.80)
POPC:POPE (3:1)	*rep1*	-139.79 (4.03)	-	-	-	-140.56 (1.73)
	rep2	-143.57 (7.43)				-142.32 (2.01)
	rep3	-146.51 (11.38)				-143.98 (1.75)
POPC:Chol (3:1)	*rep1*	-148.02 (12.69)	-148.89 (17.76)	-131.54 (4.38)	-131.54 (4.38)	-142.01 (1.80)
	rep2	-155.97 (8.17)	-142.70 (6.88)	-137.66 (4.27)	-137.66 (4.27)	-144.01 (2.29)
	rep3	-136.86 (62.01)	-142.80 (5.18)	-139.52 (4.81)	-139.52 (4.81)	-147.90 (2.41)
POPC:POPE:Chol (2:1:1)	*rep1*	-146.03 (9.40)	-136.68 (5.57)	-137.92 (5.62)	-137.92 (5.62)	-141.05 (1.80)
	rep2	-150.91 (43.34)	-140.95 (9.11)	-134.44 (6.66)	-134.44 (6.66)	-146.40 (1.99)
	rep3	-146.99 (8.90)	-143.58 (4.61)	-131.17 (3.77)	-131.17 (3.77)	-145.81 (1.79)

Supplementary Table 6. Overlaps of calculated network communities between the different IF systems and OF *b*MRP1-(ATP)$_2$. Analyses were performed in POPC:POPE:Chol (2:1:1) lipid bilayer. For a given community, overlaps were obtained by calculating the proportion of shared residues between target system and OF *b*MRP1-(ATP)$_2$.

Community name	**IF apo *b*MRP1**	***b*MRP1-(ATP)$_2$**	***b*MRP1-LTX**	***b*MRP1-LTX-(ATP)$_2$**
**NBD1 Walker A**	81.94%	87.39%	88.37%	87.79%
**NBD1 signature**	82.35%	83.08%	83.33%	84.85 %
**NBD2 Walker A**	71.32%	75.70%	72.32%	69.66%
**NBD2 signature**	71.64%	73.24%	64.63%	71.01%

Supplementary Table 7. The number of calculated raw NBD communities obtained from MD simulations performed in POPC:POPE:Chol (2:1:1). For IF apo *b*MRP1 and OF *bMRP1*-$(ATP)_2$, more than 2 communities per NBD were sometimes observed. They were however systematically corresponding to subcommunities of either Walker A or signature community described in Supplementary Table 6.

	**IF apo *b*MRP1**		***b*MRP1-$(ATP)_2$**		***b*MRP1-LTX**		***b*MRP1-LTX-$(ATP)_2$**		**OF *b*MRP1-$(ATP)_2$**	
	**NBD1**	**NBD2**	**NBD1**	**NBD2**	**NBD1**	**NBD2**	**NBD1**	**NBD2**	**NBD1**	**NBD2**
**Rep1**	**4**	**3**	2	2	2	2	2	**3**	2	**3**
**Rep2**	2	2	2	2	2	2	2	2	2	**4**
**Rep3**	**3**	**3**	2	2	2	2	2	2	2	2

Supplementary Table 8. Key distances between ATP and *b*MRP1 NBD or IC-loop for a) OF *b*MRP1-(ATP)$_2$, b) *b*MRP1-LTX-(ATP)$_2$ and c) *b*MRP1-(ATP)$_2$. Values were averaged over each replica; standard deviations are reported in brackets. The highest difference for the signature motif is highlighted by a red box.

a) OF *b*MRP1-(ATP)$_2$

Membrane	Replica	ATP1										
		W653 (A-loop)	K684 (Walker A)	S685 (Walker A)	W653 (A-loop)	S1430 (Signature)	PG				W653 (A-loop)	6 amino group
							D793 (Walker B)	G770 (Signature)	Q713 (Q-loop)	H827 (H-loop)	G770 Signature)	N412
POPC	1	4.48 (0.35)	4.34 (0.09)	5.03 (0.07)	7.06 (0.09)	4.72 (0.15)	9.33 (0.32)	17.29 (0.36)	7.44 (0.24)	8.61 (0.41)	20.88 (0.34)	5.41 (0.74)
	2	4.38 (0.31)	4.38 (0.13)	5.02 (0.07)	7.05 (0.09)	4.72 (0.14)	9.32 (0.43)	17.23 (0.54)	7.36 (0.31)	8.8 (0.4)	20.71 (0.63)	5.4 (0.77)
	3	4.99 (0.52)	4.35 (0.08)	5.04 (0.07)	7.03 (0.09)	5.57 (0.52)	9.09 (0.37)	16.86 (0.38)	7.52 (0.28)	8.64 (0.43)	19.76 (0.53)	6.2 (0.91)
POPC:Chol (3:1)	1	4.68 (0.45)	4.42 (0.11)	5.03 (0.07)	7.08 (0.1)	4.7 (0.13)	9.27 (0.35)	16.46 (0.41)	6.76 (0.16)	8.94 (0.39)	19.76 (0.48)	6.35 (1.2)
	2	5.16 (0.3)	4.37 (0.08)	5.03 (0.07)	7.02 (0.09)	4.86 (0.31)	9.14 (0.37)	15.86 (0.59)	7.21 (0.19)	8.66 (0.51)	18.6 (0.73)	6.59 (0.64)
	3	5.38 (0.42)	4.38 (0.08)	5.07 (0.07)	7.07 (0.1)	5.71 (0.22)	8.58 (0.37)	16.1 (0.73)	7.09 (0.19)	9.62 (0.66)	18.24 (0.6)	7.71 (0.93)
POPC:POPE: Chol (2:1:1)	1	4.59 (0.35)	4.36 (0.08)	5.02 (0.07)	7.04 (0.09)	4.71 (0.12)	11.43 (1.12)	17.43 (0.49)	6.68 (0.22)	8.92 (0.39)	20.93 (0.44)	5.02 (0.64)
	2	4.72 (0.57)	4.41 (0.11)	5.02 (0.07)	7.05 (0.1)	4.74 (0.2)	9.3 (0.33)	17.61 (0.48)	6.79 (0.17)	8.92 (0.41)	20.74 (0.67)	5.68 (1.36)
	3	4.64 (0.37)	4.44 (0.15)	5.02 (0.07)	7.06 (0.1)	4.68 (0.12)	9.17 (0.39)	17.64 (0.44)	6.94 (0.26)	8.85 (0.42)	21.12 (0.36)	7.35 (1.34)

Membrane	Replica	ATP2										
		Y1301 (A-loop)	K1332 (Walker A)	S1333 (Walker A)	S1334 (Walker A)	G771 (Signature)	PG				Ribose ring	6 amino group
							E1454 (Walker B)	V1431 (Signature)	Q1374 (Q-loop)	H1485 (H-loop))	V1431 (Signature)	E1064
POPC	1	4.62 (0.35)	4.49 (0.09)	5.07 (0.07)	7.09 (0.1)	6.94 (0.17)	8.3 (0.31)	16.55 (0.29)	6.25 (0.15)	8.19 (0.68)	20.09 (0.29)	6.1 (0.79)
	2	4.67 (0.74)	4.49 (0.1)	5.09 (0.08)	7.1 (0.1)	7.72 (1.28)	8.29 (0.76)	17.03 (0.73)	6.49 (0.37)	8.88 (0.88)	20.34 (0.41)	6.3 (1.29)
	3	4.45 (0.33)	4.55 (0.11)	5.06 (0.07)	7.07 (0.1)	6.97 (0.18)	8.2 (0.57)	14.05 (1.27)	6.34 (0.16)	9.26 (1.19)	17.55 (1.4)	5.65 (0.74)

**POPC:Chol (3:1)**	**1**	4.36 (0.33)	4.62 (0.18)	5.06 (0.09)	7.01 (0.12)	8.26 (0.46)	6.8 (0.22)	17.61 (0.39)	7.29 (0.2)	9.6 (1.19)	20.1 (0.35)	5.53 (1.0)
	**2**	4.38 (0.3)	4.65 (0.22)	5.07 (0.09)	7.02 (0.12)	8.81 (0.75)	6.55 (0.22)	15.68 (0.7)	7.08 (0.27)	10.28 (0.64)	18.24 (0.61)	4.92 (0.8)
	**3**	5.07 (0.84)	4.8 (0.16)	5.1 (0.08)	7.01 (0.11)	9.52 (0.66)	6.62 (0.17)	14.22 (0.81)	7.03 (0.26)	7.82 (0.65)	16.6 (0.86)	6.48 (1.88)
**POPC:POPE: Chol (2:1:1)**	**1**	4.56 (0.38)	4.75 (0.15)	5.04 (0.07)	7.12 (0.12)	6.89 (0.23)	7.92 (0.46)	16.34 (0.49)	6.22 (0.13)	10.33 (1.27)	20.08 (0.49)	5.18 (0.55)
	**2**	4.51 (0.3)	4.51 (0.12)	5.03 (0.07)	7.1 (0.11)	7.05 (0.17)	9.23 (0.42)	16.5 (0.61)	6.16 (0.16)	10.0 (0.83)	20.24 (0.6)	4.99 (0.44)
	**3**	4.64 (0.36)	4.62 (0.15)	5.01 (0.07)	7.09 (0.1)	6.9 (0.18)	8.23 (0.98)	16.66 (0.37)	6.13 (0.11)	10.74 (0.74)	20.37 (0.37)	6.45 (0.8)

b) *b*MRP1-LTX-(ATP)$_2$

**Membrane**	**Replica**	**ATP1**										
							**PG**				**Ribose ring**	**6 amino group**
		**W653 (A-loop)**	**K684 (Walker A)**	**S685 (Walker A)**	**S686 (Walker A)**	**S1430 (Signature)**	**D793 (Walker B)**	**G770 (Signature)**	**Q713 (Q-loop)**	**H827**	**G770**	**N412**
**POPC**	**1**	4.94 (0.52)	4.64 (0.36)	5.12 (0.11)	7.07 (0.12)	8.33 (2.9)	9.63 (0.63)	16.58 (0.89)	7.54 (1.02)	10.57 (1.47)	19.13 (0.68)	5.56 (1.3)
	**2**	5.97 (1.26)	4.41 (0.1)	5.11 (0.08)	7.09 (0.1)	18.46 (8.73)	9.08 (0.54)	16.04 (0.7)	6.92 (0.21)	9.4 (1.05)	18.06 (0.88)	6.68 (1.79)
	**3**	5.43 (0.7)	4.49 (0.11)	5.17 (0.09)	6.94 (0.1)	7.29 (4.33)	12.0 (0.48)	16.21 (0.67)	7.01 (0.31)	8.96 (0.94)	18.8 (0.77)	8.64 (1.91)
**POPC:Chol (3:1)**	**1**	9.14 (3.24)	4.42 (0.11)	5.08 (0.08)	7.07 (0.12)	10.23 (4.32)	9.24 (0.55)	16.07 (0.64)	7.06 (0.27)	9.75 (1.0)	18.78 (0.9)	12.84 (7.44)
	**2**	5.47 (1.56)	4.46 (0.14)	5.12 (0.08)	7.06 (0.1)	20.6 (1.74)	8.6 (0.45)	15.67 (0.61)	7.15 (0.28)	8.64 (0.65)	17.16 (0.8)	7.22 (3.78)
	**3**	6.76 (2.73)	4.43 (0.12)	5.09 (0.09)	7.09 (0.15)	17.95 (3.19)	8.97 (0.54)	16.32 (0.74)	7.02 (0.26)	9.85 (0.92)	18.62 (0.96)	9.35 (5.62)
**POPC:POPE:C hol (2:1:1)**	**1**	6.81 (2.4)	4.46 (0.16)	5.11 (0.1)	7.08 (0.11)	9.89 (2.03)	8.71 (0.56)	15.6 (0.71)	7.02 (0.24)	8.74 (0.89)	17.48 (0.96)	9.08 (5.41)
	**2**	5.92 (1.61)	4.41 (0.11)	5.1 (0.08)	7.07 (0.1)	9.58 (1.35)	8.76 (0.62)	16.63 (0.95)	7.03 (0.27)	9.58 (1.17)	18.38 (1.28)	8.45 (2.75)
	**3**	7.12 (2.42)	4.41 (0.13)	5.1 (0.08)	7.11 (0.16)	10.25 (1.53)	9.04 (0.61)	16.99 (0.62)	6.87 (0.24)	9.41 (1.27)	18.5 (0.94)	7.56 (3.06)

Membrane	Replica	ATP2										
		Y1301 (A-loop)	K1332 (Walker A)	S1333 (Walker A)	S1334 (Walker A)	G771 (Signature)	PG				Ribose ring	6 amino group
							E1454 (Walker B)	V1431 (Signature)	Q1374 (Q-loop)	H1485	V1431	E1064
POPC	1	9.39 (0.61)	4.53 (0.09)	5.15 (0.09)	6.91 (0.1)	11.04 (2.19)	6.4 (0.31)	16.97 (0.49)	6.75 (0.25)	6.72 (0.62)	19.92 (0.95)	11.91 (1.25)
	2	10.01 (1.31)	4.53 (0.11)	5.15 (0.09)	6.92 (0.09)	23.93 (8.47)	6.02 (0.43)	17.05 (0.84)	6.92 (0.28)	9.45 (1.61)	19.45 (0.96)	7.63 (2.85)
	3	12.68 (1.49)	4.53 (0.09)	5.09 (0.09)	6.92 (0.1)	13.67 (2.73)	6.49 (0.32)	17.53 (0.94)	6.46 (0.29)	6.4 (0.53)	20.62 (1.04)	11.21 (2.63)
POPC:Chol (3:1)	1	10.79 (1.85)	4.53 (0.1)	5.17 (0.09)	6.94 (0.1)	14.25 (4.12)	7.17 (0.53)	14.83 (1.06)	6.84 (0.29)	11.27 (0.93)	17.51 (1.27)	13.76 (6.93)
	2	10.46 (2.99)	4.48 (0.11)	5.11 (0.08)	7.07 (0.1)	22.66 (1.79)	6.56 (0.67)	15.38 (1.35)	6.7 (0.2)	8.51 (0.99)	17.84 (1.47)	7.11 (1.8)
	3	10.85 (0.98)	4.48 (0.11)	5.08 (0.09)	7.06 (0.12)	13.91 (1.17)	6.87 (0.54)	16.7 (0.78)	6.74 (0.26)	9.53 (1.33)	19.78 (0.87)	9.97 (2.61)
POPC:POPE:Chol (2:1:1)	1	8.59 (0.85)	4.51 (0.09)	5.15 (0.09)	6.94 (0.1)	13.93 (1.63)	12.35 (1.11)	17.02 (1.01)	6.7 (0.31)	9.8 (0.98)	20.24 (1.23)	5.36 (1.12)
	2	8.4 (1.3)	4.63 (0.16)	5.14 (0.09)	6.93 (0.11)	13.27 (1.29)	6.85 (0.36)	14.12 (1.04)	6.63 (0.24)	12.58 (1.17)	17.52 (1.51)	13.45 (5.28)
	3	6.19 (2.0)	4.48 (0.12)	5.11 (0.09)	7.06 (0.1)	15.85 (1.71)	7.65 (0.55)	17.13 (0.6)	6.97 (0.25)	10.55 (0.54)	19.25 (0.8)	10.96 (6.62)

c) *b*MRP1-(ATP)$_2$

Membrane	Replica	ATP1										
		W653 (A-loop)	K684 (Walker A)	S685 (Walker A)	S686 (Walker A)	S1430 (Signature)	PG				Ribose ring	6 amino group
							D793 (Walker B)	G770 (Signature)	Q713 (Q-loop)	H827	G770	N412
POPC	1	5.32 (0.47)	4.48 (0.1)	5.08 (0.09)	6.9 (0.09)	33.15 (5.18)	12.42 (0.6)	16.06 (0.53)	6.08 (0.17)	9.18 (1.15)	19.45 (0.59)	4.82 (1.04)
	2	6.27 (2.03)	4.87 (0.23)	5.09 (0.1)	7.07 (0.12)	34.74 (3.66)	9.52 (0.8)	15.82 (0.65)	6.24 (0.13)	10.99 (1.17)	19.15 (0.83)	9.21 (4.7)
	3	5.07 (0.73)	4.5 (0.14)	5.09 (0.08)	6.89 (0.09)	15.59 (3.87)	12.2 (0.62)	16.01 (0.51)	6.13 (0.18)	9.09 (0.86)	19.4 (0.61)	5.85 (1.62)

POPC:Chol (3:1)	1	5.15 (0.45)	4.49 (0.14)	5.08 (0.08)	6.89 (0.09)	12.38 (5.73)	12.01 (0.74)	15.95 (0.54)	6.07 (0.18)	8.98 (0.73)	19.49 (0.6)	6.07 (1.35)
	2	5.37 (0.52)	4.46 (0.1)	5.09 (0.08)	6.91 (0.09)	12.23 (3.96)	12.39 (0.5)	16.21 (0.52)	6.04 (0.17)	9.51 (1.01)	19.6 (0.62)	5.26 (1.01)
	3	5.47 (0.57)	4.47 (0.09)	5.09 (0.08)	6.91 (0.09)	12.65 (5.29)	12.17 (0.49)	15.98 (0.51)	6.11 (0.23)	9.4 (0.92)	19.26 (0.6)	5.76 (1.25)
POPC:POPE:Chol (2:1:1)	1	5.17 (0.5)	4.53 (0.16)	5.09 (0.08)	6.89 (0.09)	10.93 (3.2)	11.89 (0.6)	15.75 (0.61)	6.18 (0.21)	8.68 (1.17)	19.05 (0.62)	5.51 (1.0)
	2	5.6 (1.16)	6.44 (1.42)	5.59 (0.45)	7.15 (0.5)	11.18 (4.85)	10.95 (0.85)	15.37 (0.86)	6.23 (0.17)	13.21 (1.25)	18.53 (0.96)	8.12 (3.14)
	3	5.1 (0.5)	4.52 (0.17)	5.1 (0.09)	6.9 (0.1)	23.86 (2.34)	12.32 (0.83)	16.04 (0.8)	6.13 (0.19)	10.01 (1.53)	19.52 (0.81)	5.73 (1.19)

Membrane	Replica	ATP2										
							PG				Ribose ring	6 amino group
		Y1301 (A-loop)	K1332 (Walker A)	S1333 (Walker A)	S1334 (Walker A)	G771 (Signature)	E1454 (Walker B)	V1431 (Signature)	Q1374 (Q-loop)	H1485	V1431	E1064
POPC	1	9.46 (1.03)	4.52 (0.1)	5.12 (0.09)	6.94 (0.1)	31.35 (5.06)	8.57 (0.63)	16.49 (0.69)	6.1 (0.17)	11.12 (1.33)	19.54 (0.77)	6.63 (1.56)
	2	9.42 (1.19)	4.56 (0.12)	5.12 (0.09)	6.91 (0.1)	37.96 (3.45)	10.08 (0.56)	15.25 (0.71)	6.14 (0.15)	12.31 (0.99)	18.91 (0.71)	9.45 (1.9)
	3	12.15 (3.24)	4.55 (0.17)	5.13 (0.09)	7.04 (0.13)	17.68 (3.05)	7.66 (1.24)	15.79 (1.3)	6.81 (0.2)	8.76 (1.21)	17.37 (1.2)	11.61 (4.82)
POPC:Chol (3:1)	1	9.52 (1.85)	4.47 (0.12)	5.15 (0.09)	7.06 (0.1)	18.29 (5.85)	8.28 (0.64)	17.0 (0.89)	7.02 (0.29)	8.27 (1.12)	19.08 (1.3)	13.52 (4.88)
	2	8.77 (2.21)	4.47 (0.11)	5.11 (0.08)	7.08 (0.12)	18.74 (3.46)	6.59 (0.56)	16.5 (1.15)	6.8 (0.18)	8.24 (1.92)	19.76 (1.47)	12.59 (5.28)
	3	9.25 (0.92)	4.52 (0.09)	5.16 (0.09)	6.95 (0.1)	15.32 (4.64)	7.5 (1.09)	15.94 (0.51)	6.68 (0.27)	7.81 (1.33)	18.85 (0.62)	6.2 (1.2)
POPC:POPE:Chol (2:1:1)	1	8.98 (1.11)	4.54 (0.14)	5.15 (0.1)	6.94 (0.1)	14.11 (2.12)	8.49 (1.05)	15.89 (1.27)	6.56 (0.28)	9.46 (1.0)	19.0 (1.21)	5.63 (1.43)
	2	8.55 (1.61)	4.51 (0.09)	5.14 (0.09)	6.94 (0.1)	15.02 (3.44)	9.88 (1.05)	16.66 (0.62)	5.94 (0.15)	11.18 (0.95)	19.77 (0.73)	6.29 (1.1)
	3	12.25 (1.67)	4.49 (0.15)	5.07 (0.1)	7.05 (0.12)	14.68 (2.04)	6.46 (0.57)	17.22 (0.52)	6.63 (0.18)	6.78 (0.67)	20.39 (0.59)	11.24 (0.76)

Supplementary Table 9. Selected residues for allosteric pathway calculations from substrate-binding site to NBS1 and NBS2.

Sink	Sources	
LTX-binding site	***NBS1***	***NBS2***
K332<br>Y440<br>R593<br>R1196<br>E1203<br>T1241<br>N1244<br>W1245<br>R1248	Walker A^{NBD1}:<br>G681, G683, K684, S686	Walker A^{NBD2}:<br>R1328, T1329, A1331, G1332, S1334
	Q-loop^{NBD1}:<br>Q713	Q-loop^{NBD2}:<br>Q1374
	A-loop^{NBD1}:<br>W653	A-loop^{NBD2}:<br>Y1301
	ABC Signature^{NBD2}:<br>S1430, V1431, G1432, Q1433	ABC Signature^{NBD1}:<br>S769, G770, G771, Q772

Supplementary Table 10. Membrane free energy deformations. Bilayer Thicknesses at the outer edge of the simulation cells in POPC:POPE:Chol (2:1:1).

		**IF apo *b*MRP1**	***b*MRP1-$(ATP)_2$**	***b*MRP1-LTX**	***b*MRP1-LTX-$(ATP)_2$**	**OF *b*MRP1-$(ATP)_2$**
d_0 (Å)	*rep1*	42.4	44.6	45.0	44.8	44.6
	rep2	42.0	44.0	44.6	44.5	44.6
	rep3	42.2	44.3	44.6	44.7	44.5
Free energy cost of deformation due to membrane protein (kT)	*rep1*	60.2	42.9	47.7	43.7	42.5
	rep2	54.3	53.4	56.3	50.6	48.9
	rep3	66.3	46.6	49.3	47.8	46.7
Reference free energy cost for flat membrane (kT)	*rep1*	65.9	58.6	60.5	61.1	58.6
	rep2	65.9	59.1	62.9	59.5	59.1
	rep3	67.7	59.7	61.7	60.6	59.7
Number of iterations	*rep1*	150	150	150	130	150
	rep2	150	150	150	130	150
	rep3	150	150	150	150	150
Assumed ΔG (Deformation - flat) (kT)	*rep1*	-5.7	-15.7	-14.0	-17.4	-16.1
	rep2	11.6	-5.7	-5.4	-8.9	-10.2
	rep3	-1.4	-13.1	-12.4	-12.8	-13.0

Supplementary Table 11. Membrane free energy deformations. Bilayer Thicknesses at the outer edge of the simulation cells in POPC:Chol (3:1).

		**IF apo *b*MRP1**	***b*MRP1-$(ATP)_2$**	***b*MRP1-LTX**	***b*MRP1-LTX-$(ATP)_2$**	**OF *b*MRP1-$(ATP)_2$**
d_0 (Å)	*rep1*	40.0	42.7	42.8	42.9	42.9
	rep2	40.7	43.4	43.1	43.8	41.8
	rep3	40.7	43.3	43.8	43.2	42.3
Free energy cost of deformation due to membrane protein (kT)	*rep1*	54.9	32.8	26.2	35.5	30.1
	rep2	43.3	32.1	25.7	31.5	23.6
	rep3	56.3	30.7	33.7	30.5	32.1
Reference free energy cost for flat membrane (kT)	*rep1*	30.2	28.6	26.7	27.6	29.3
	rep2	31.4	28.4	26.9	26.1	27.7
	rep3	29.9	28.6	25.8	26.0	30.4
Number of iterations	*rep1*	150	150	150	150	150
	rep2	150	150	150	150	150
	rep3	150	150	150	150	150
Assumed ∆G (Deformation - flat) (kT)	*rep1*	14.7	4.2	-0.5	7.9	0.8
	rep2	10.9	3.7	-1.2	5.3	-4.1
	rep3	16.4	2.1	7.9	4.5	1.7

Supplementary Table 12. Membrane free energy deformations. Bilayer Thicknesses at the outer edge of the simulation cells in pure POPC.

		IF apo *b*MRP1	*b*MRP1-$(ATP)_2$	*b*MRP1-LTX	*b*MRP1-LTX-$(ATP)_2$	OF *b*MRP1-$(ATP)_2$
d_0 (Å)	*rep1*<br>*rep2*<br>*rep3*	36.6<br>36.7<br>36.8	38.0<br>38.4<br>38.3	38.5<br>37.7<br>38.9	38.7<br>38.2<br>37.9	35.1<br>33.4<br>35.8
Free energy cost of deformation due to membrane protein (kT)	*rep1*<br>*rep2*<br>*rep3*	15.2<br>19.9<br>18.0	20.9<br>16.7<br>-	12.3<br>16.7<br>9.9	11.9<br>12.2<br>14.4	18.6<br>22.5<br>18.0
Reference free energy cost for flat membrane (kT)	*rep1*<br>*rep2*<br>*rep3*	1.0<br>0.9<br>1.0	1.0<br>0.9<br>-	0.8<br>0.9<br>0.9	0.9<br>0.9<br>0.9	1.0<br>1.1<br>1.1
Number of iterations	*rep1*<br>*rep2*<br>*rep3*	150<br>150<br>150	150<br>150<br>-	150<br>150<br>150	150<br>150<br>150	150<br>150<br>150
Assumed ∆G (Deformation - flat) (kT)	*rep1*<br>*rep2*<br>*rep3*	14.2<br>19.0<br>17.0	19.9<br>16.8<br>-	11.5<br>16.8<br>9.0	11.0<br>11.3<br>13.5	17.6<br>21.4<br>16.9

Supplementary Table 13. L_0 modelling of OF state.

269-RKQPVKIV-276	L_0 of IF system
277-YSSKDPAKPKGSSKVDV-293	sequence
294-NEEAEALIVKCPQKERD-310	L_0 of IF system

Supplementary Table 14. Box sizes (Å) of each system considered in the present study.

Membranes	System Setup	IF apo bMRP1	IF bMRP1-(ATP)2	IF bMRP1-LTX	IF bMRP1-LTX-(ATP)2	OF bMRP1-(ATP)2
POPC	Box size (x, A)	121.5	121.6	121.6	121.6	121.5
	Box size (y, A)	121.2	121.2	121.5	121.5	121.6
	Box size (z, A)	179.8	179.8	179.8	179.8	179.5
	Natoms	240956	241044	241408	241492	242975
	Nwater	57444	57444	57654	57654	57905
	NNa+	155	155	156	156	156
	NCl-	160	160	161	157	160
	NPOPC	364	364	362	362	363
	NChol	0	0	0	0	3
	NPOPE	0	0	0	0	0
POPE	Box size (x, A)	119.6	-	-	-	121.5
	Box size (y, A)	119.6	-	-	-	121.6
	Box size (z, A)	178.3	-	-	-	179.5
	Natoms	244324	-	-	-	247676
	Nwater	57326	-	-	-	58104
	NNa+	154				156
	NCl-	159	-	-	-	156
	NPOPC	0	-	-	-	0
	NChol	0	-	-	-	3
	NPOPE	420	-	-	-	422

Supplementary Table 14. *(Next)*

Membranes	System Setup	IF apo bMRP1	IF bMRP1-(ATP)2	IF bMRP1-LTX	IF bMRP1-LTX-(ATP)2	OF bMRP1-(ATP)2
POPC:POPE (3:1)	Box size (x, A)	121.5	-	-	-	121.5
	Box size (y, A)	121.6	-	-	-	121.5
	Box size (z, A)	179.7	-	-	-	179.5
	Natoms	242943	-	-	-	243828
	Nwater	57763	-	-	-	57892
	NNa+	155				156
	NCl-	160	-	-	-	156
	NPOPC	284	-	-	-	282
	NChol	0	-	-	-	3
	NPOPE	94	-	-	-	94
POPC:Chol (3:1)	Box size (x, A)	123	123	121.9	121.9	123.1
	Box size (y, A)	123.7	123.7	122.8	122.8	126.3
	Box size (z, A)	179.7	179.8	179.2	179.2	179.5
	Natoms	248527	248615	242640	242724	244008
	Nwater	59517	59517	58138	58138	58324
	NNa+	162	162	158	158	158
	NCl-	167	167	163	159	158
	NPOPC	316	316	304	304	305
	NChol	105	105	102	102	105
	NPOPE	0	0	0	0	0
POPC:POPE:Chol (2:1:1)	Box size (x, A)	121.2	121.2	122.8	122.8	123.1
	Box size (y, A)	122.4	122.4	122.3	122.3	126.3
	Box size (z, A)	179.7	179.7	179.2	179.2	179.5
	Natoms	241020	241034	244089	244173	245145
	Nwater	57379	57379	58215	58215	58408
	NNa+	155	155	158	158	158
	NCl-	160	160	163	159	158
	NPOPC	210	210	212	212	212
	NChol	105	104	106	106	108
	NPOPE	105	105	106	106	105

Supplementary Table 15. Distances used to originally restrain Mg2+-ATP-NBD arrangement in MD simulations. Distances were measured on the minimized structure (OF *b*MRP1-(ATP)$_2$) and on the starting structure (*b*MRP1-(ATP)$_2$ and *b*MRP1-LTX-(ATP)$_2$)

	**OF *b*MRP1-(ATP)$_2$**					***b*MRP1-(ATP)$_2$ *b*MRP1-LTX-(ATP)$_2$**
	POPC	POPE	POPC:POPE (3:1)	POPC:Chol (3:1)	POPC:POPE:Chol (2:1:1)	POPC POPC:Chol (3:1) POPC:POPE:Chol (2:1:1)
ATP$_{NBD1}$ – Trp653	3.22	3.46	3.45	3.56	3.57	3.50
ATP$_{NBD1}$(Pα) – Ser686	2.74	2.80	2.80	2.78	2.78	2.71
ATP$_{NBD1}$(Pα) – Ser685	3.43	3.16	3.16	3.14	3.16	3.07
ATP$_{NBD1}$(Pβ) – Ser685	2.76	2.86	2.85	2.77	2.76	2.83
ATP$_{NBD1}$(Pβ) – Lys684	2.73	2.79	2.78	2.75	2.75	2.79
ATP$_{NBD1}$(Pγ) – Ser1430	2.49	2.68	2.67	2.65	2.66	-
ATP$_{NBD1}$(Pγ) – Lys684	2.50	2.64	2.61	2.68	2.68	2.71
ATP$_{NBD2}$ – Tyr1301	3.55	3.52	3.55	3.52	3.52	3.60
ATP$_{NBD2}$(Pα) – Ser1334	2.61	2.62	2.62	2.63	2.63	2.70
ATP$_{NBD2}$(Pα) – Ser1334	3.5	2.99	2.97	2.96	3.00	2.62

$ATP_{NBD2}(P\beta)$ – Ser1333	2.61	2.75	2.75	2.74	2.73	2.76
$ATP_{NBD2}(P\beta)$ – Lys1332	3.01	2.71	2.71	2.70	2.70	2.71
$ATP_{NBD2}(P\gamma)$ – Gly771	3.61	3.00	3.01	3.00	3.04	-
$ATP_{NBD2}(P\gamma)$ – Lys1332	2.63	2.74	2.73	2.77	2.77	2.74
Mg^{2+}_{NBD1} – $ATP_{NBD1}(P\alpha)$	1.88	1.86	1.90	1.90	1.90	1.88
Mg^{2+}_{NBD1} – $ATP_{NBD1}(P\beta)$	1.93	1.94	1.94	1.93	1.93	1.91
Mg^{2+}_{NBD1} – $ATP_{NBD1}(P\gamma)$	1.81	1.83	1.83	1.83	1.83	1.83
Mg^{2+}_{NBD1} – Ser685	1.95	1.98	1.98	1.98	1.98	2.06
Mg^{2+}_{NBD1} – Gln713	2.59	2.06	2.06	2.01	2.01	2.09
Mg^{2+}_{NBD2} – $ATP_{NBD2}(P\alpha)$	1.91	1.95	1.94	1.95	1.96	1.93
Mg^{2+}_{NBD2} – $ATP_{NBD2}(P\beta)$	1.87	1.89	1.90	1.90	1.90	1.90
Mg^{2+}_{NBD2} – $ATP_{NBD2}(P\gamma)$	2.32	1.86	1.86	1.87	1.87	1.87
Mg^{2+}_{NBD2} – Ser1333	2.06	2.00	1.99	2.01	2.02	2.05
Mg^{2+}_{NBD2} – Gln1374	2.52	2.03	2.03	2.03	2.04	1.97

Supplementary Table 16. Force constants (in kcal.mol^{-1}.Å^{-2}) used to restrain Mg2+-ATP-NBD arrangement.

**Distance between ATP and the protein**	
**< 2.9 Å**	61.60 kcal.mol^{-1}.Å^{-2}
**2.9 – 3.1 Å**	30.80 kcal.mol^{-1}.Å^{-2}
**> 3.1 Å**	12.32 kcal.mol^{-1}.Å^{-2}
**Any distance between Mg^{2+} and ATP or protein**	250 kcal.mol^{-1}.Å^{-2}

Supplementary Table 17. Total length of MD simulations for each replica in nanoseconds. In total, 112.37 μs MD simulations were conducted in the present study.

		IF apo *b*MRP1	*b*MRP1-(ATP)$_2$	*b*MRP1-LTX	*b*MRP1-LTX-(ATP)$_2$	OF *b*MRP1-(ATP)$_2$
POPC	*rep1*	2510	2010	2010	2010	1520
	rep2	2510	2010	2010	2010	1520
	rep3	2510	2010	2010	2010	1520
POPE	*rep1*	2010	-	-	-	1500
	rep2	2010				1520
	rep3	2010				1520
POPC:POPE (3:1)	*rep1*	2510	-	-	-	1520
	rep2	2510				1520
	rep3	2510				1520
POPC:Chol (3:1)	*rep1*	2090	2010	2010	2010	1520
	rep2	2060	2010	2010	2010	1520
	rep3	2060	2010	2010	2010	1520
POPC:POPE:Chol (2:1:1)	*rep1*	2170	2010	2010	2010	2010
	rep2	2200	2010	2010	2010	2010
	rep3	2180	2010	2010	2010	2010

Supplementary Table 18. Parameters used for Lipid Bilayer Deformation calculation.

**Lipid Bilayer**	**Compressibility Elastic modulus Ka ($1x10^{-11}$N/Ang)**	**Bending Modulus Kc ($1x10^{-10}$ N*Ang)**	**Spontaneous monolayer curvature c_0 AA^{-1}**	**Coefficient of Surface Tension α, ($1x10^{-13}$ N/Ang)**
**POPC**	2.55	9.93	-0.0026	3
**POPC:Chol (3:1)**	6.73	11.9	-0.0144	3
**POPC:POPE:Chol (2:1:1)**	6.73	11.9	-0.0217	3

Supplementary Figures

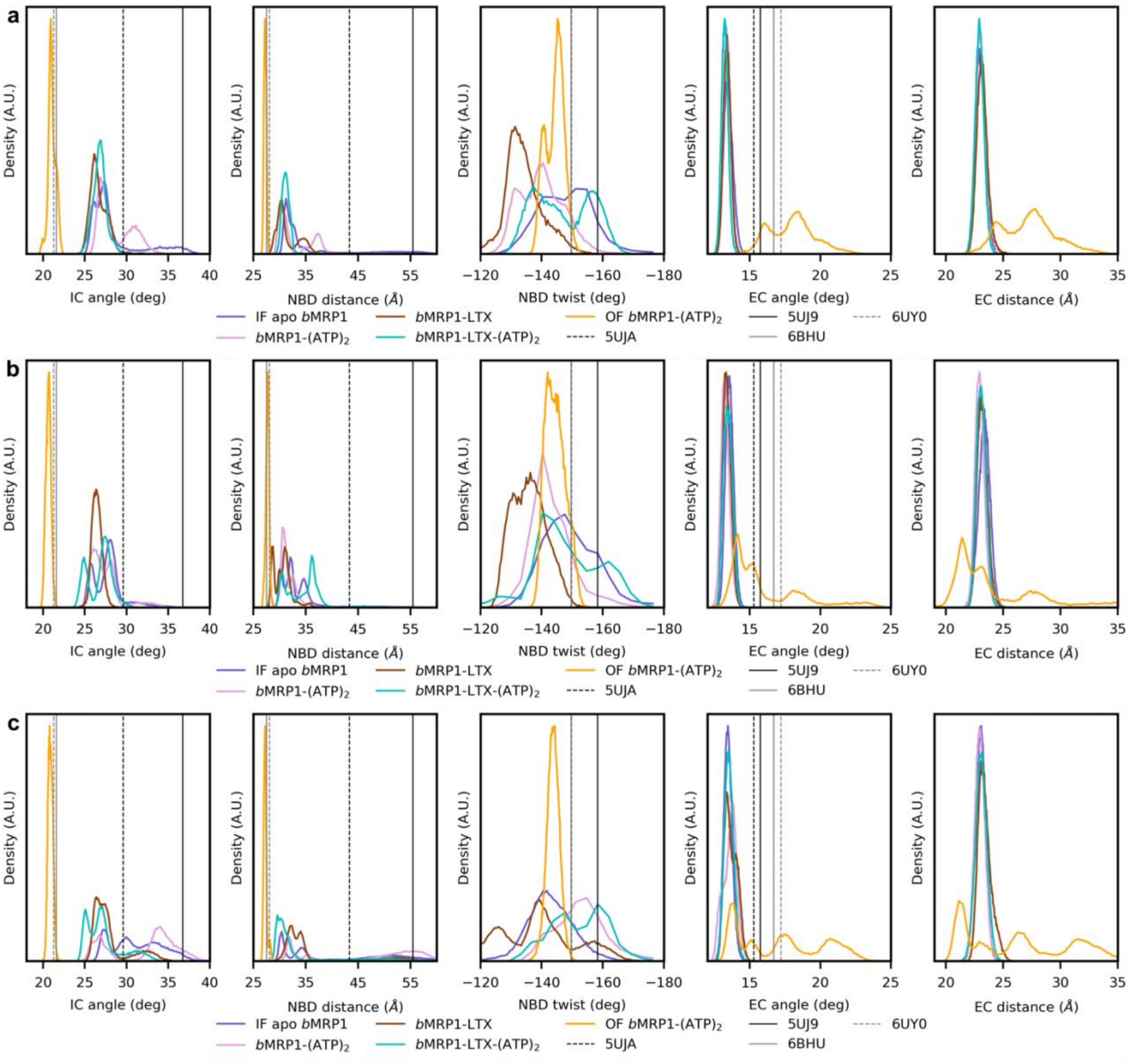

Supplementary Figure 1. Distribution of ABC structural parameters obtained in different lipid bilayer compositions. a) POPC:POPE:Chol (2:1:1) **b)** POPC:Chol (3:1) and **c)** POPC.

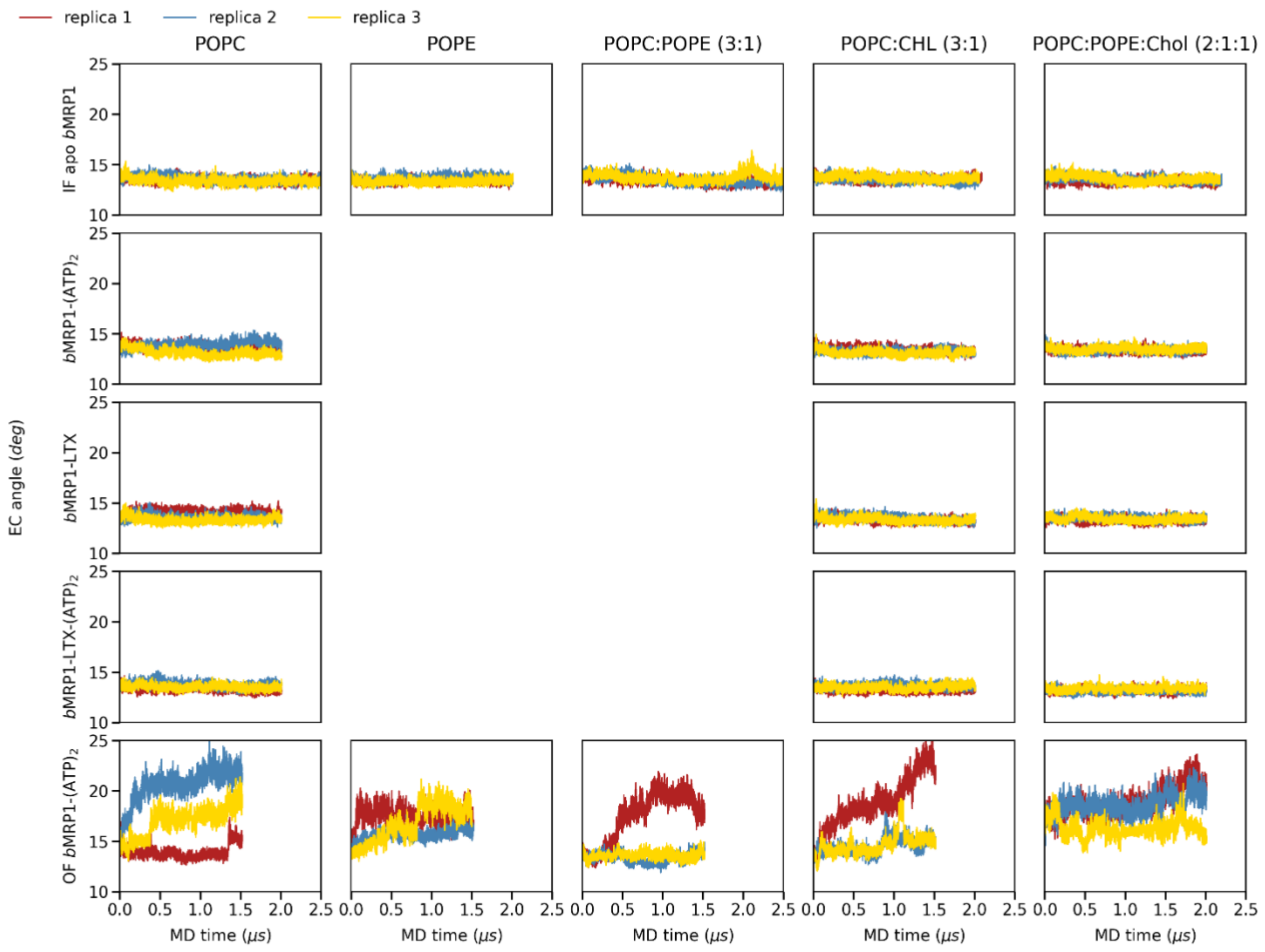

Supplementary Figure 2. Evolution of EC angle (°) along MD simulations calculated for all systems and for all different membrane compositions. Replica 1, 2 and 3 are coloured red, blue and yellow, respectively.

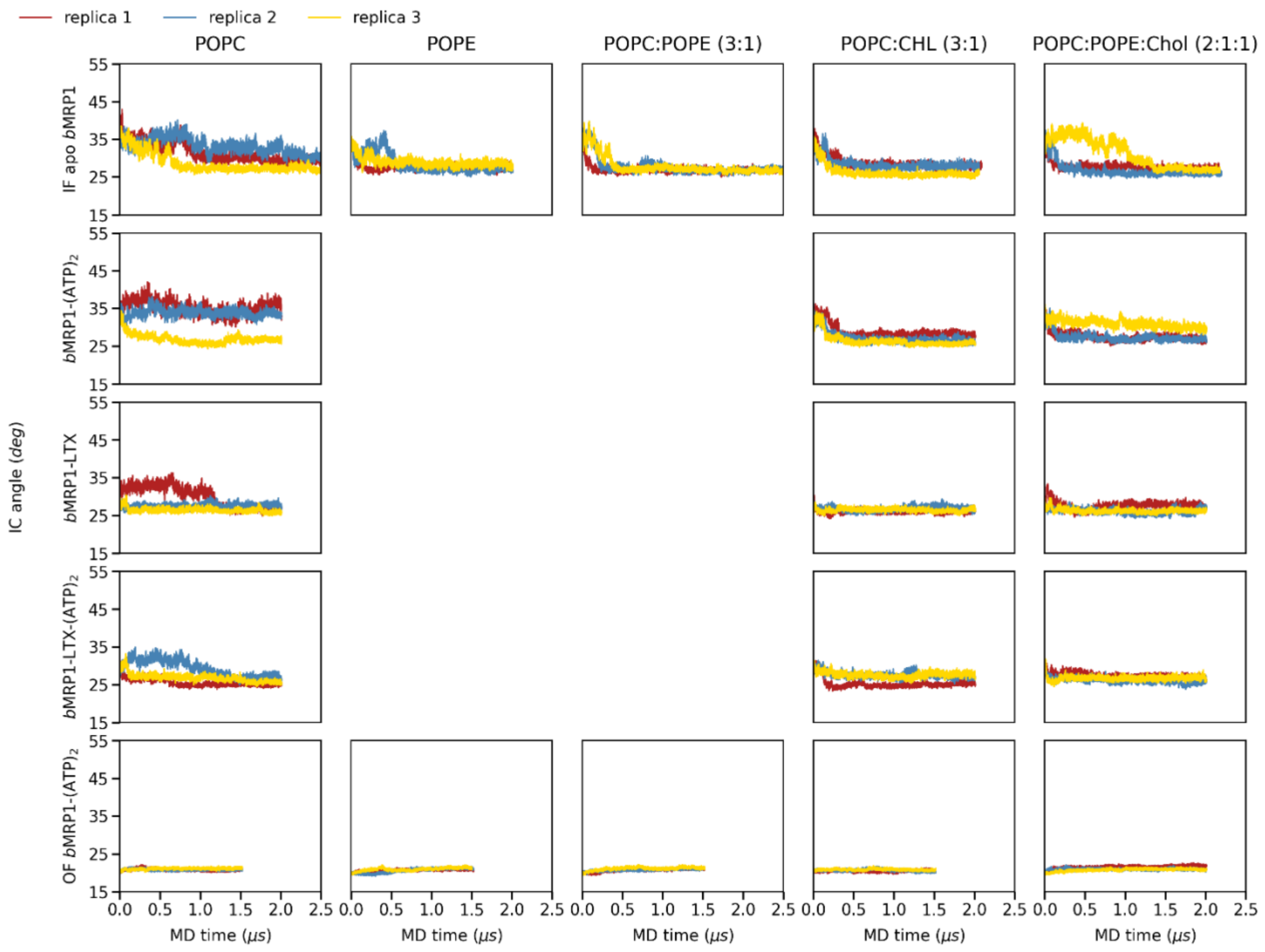

Supplementary Figure 3. IC angle (°) during the whole simulations calculated for all systems and for all different membrane compositions. Replica 1, 2 and 3 are coloured red, blue and yellow, respectively.

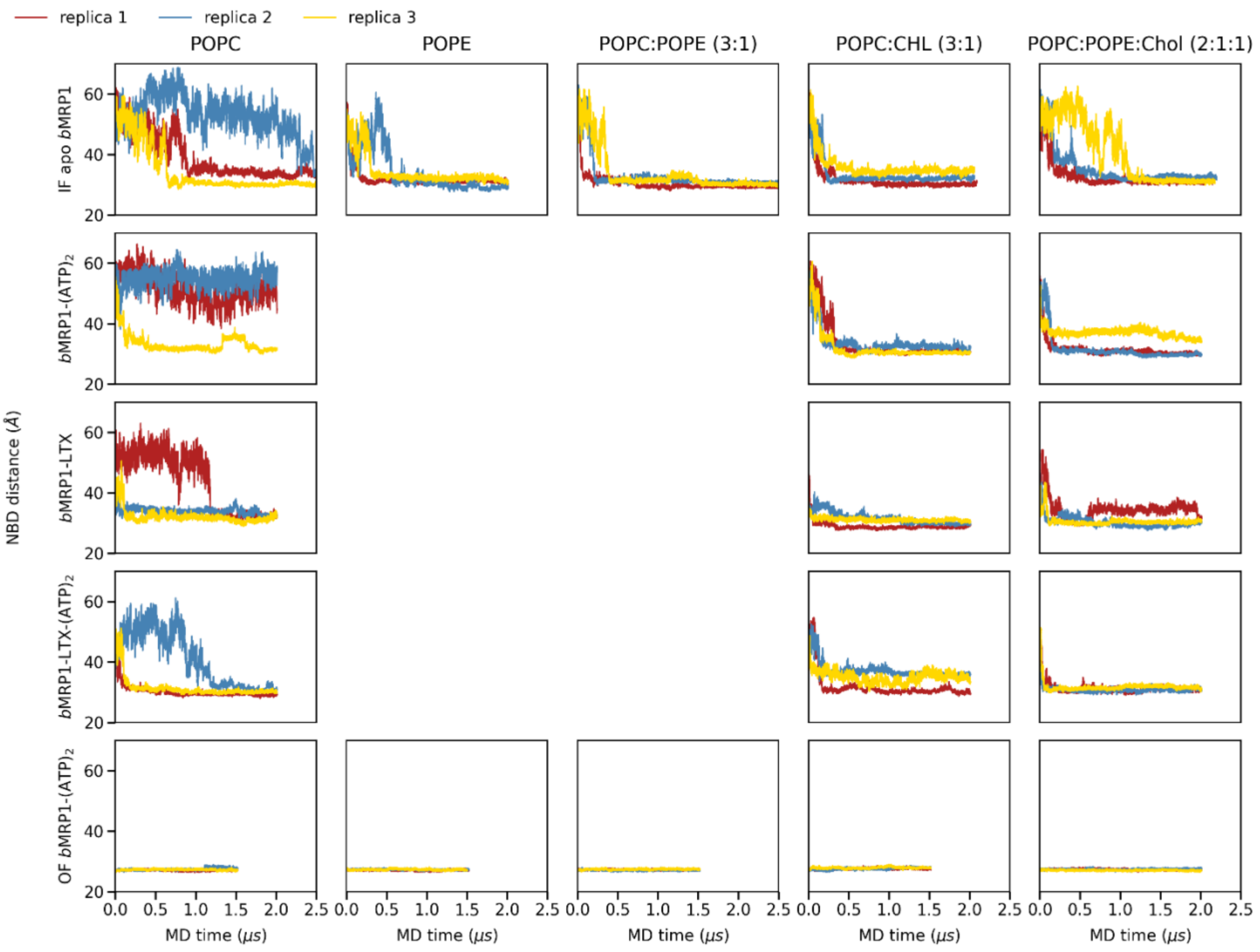

Supplementary Figure 4. NBD distance (Å) during the whole simulations calculated for all systems and for all different membrane compositions. Replica 1, 2 and 3 are coloured red, blue and yellow, respectively.

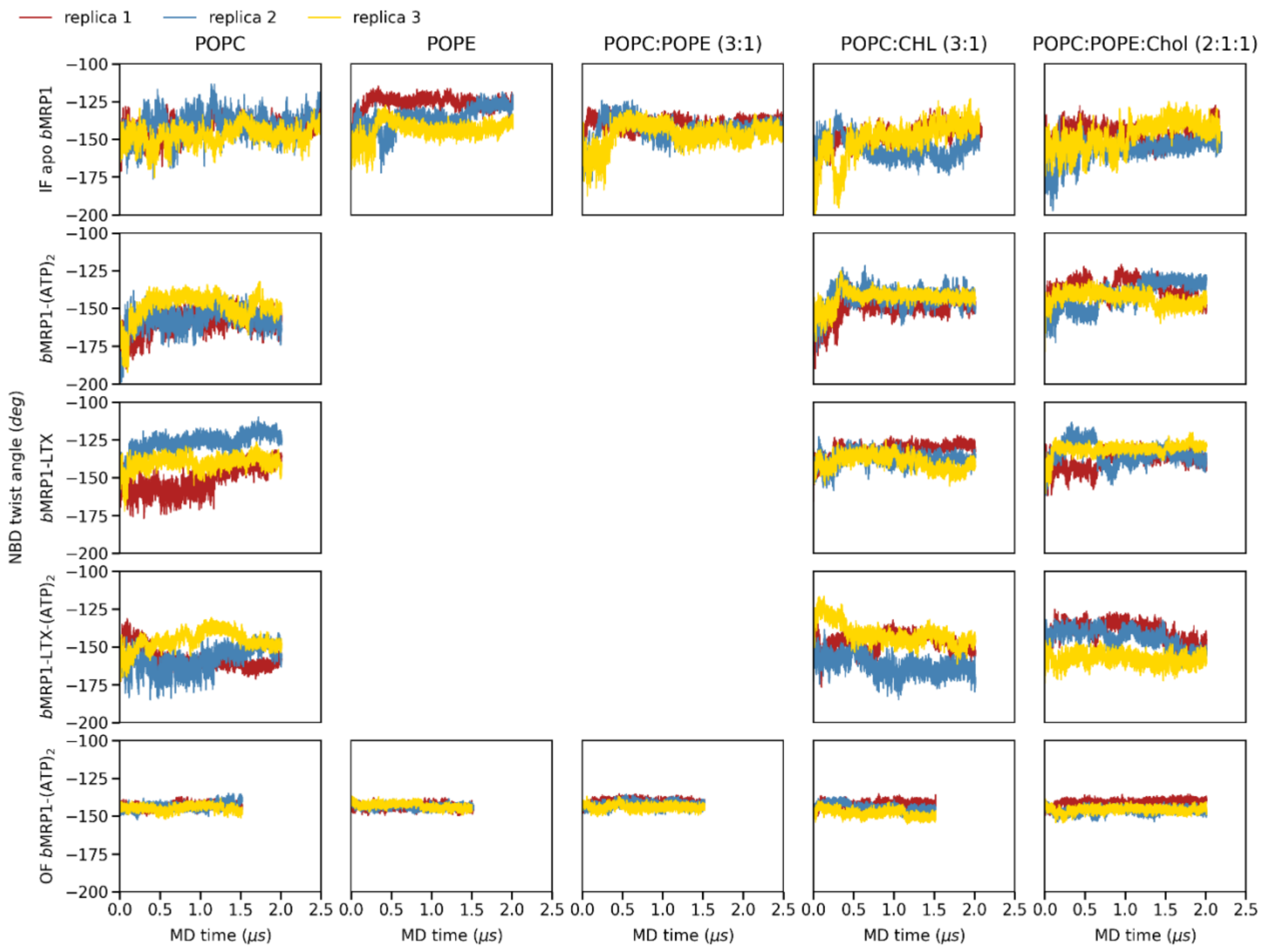

Supplementary Figure 5. NBD twist angle (°) during the whole simulations calculated for all systems and for all different membrane compositions. Replica 1, 2 and 3 are coloured red, blue and yellow, respectively.

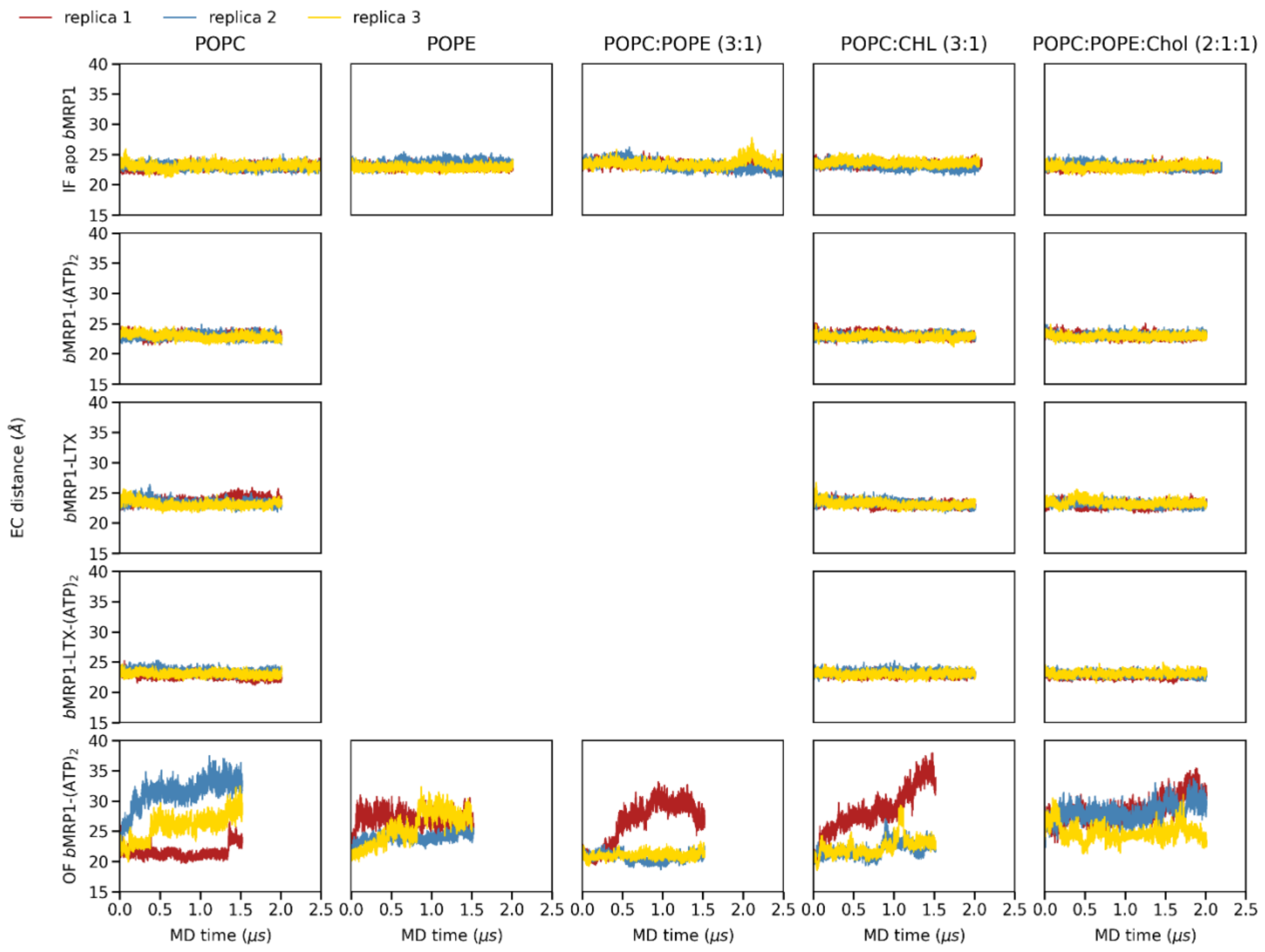

Supplementary Figure 6. EC distance (Å) during the whole simulations calculated for all systems and for all different membrane compositions. Replica 1, 2 and 3 are coloured red, blue and yellow, respectively.

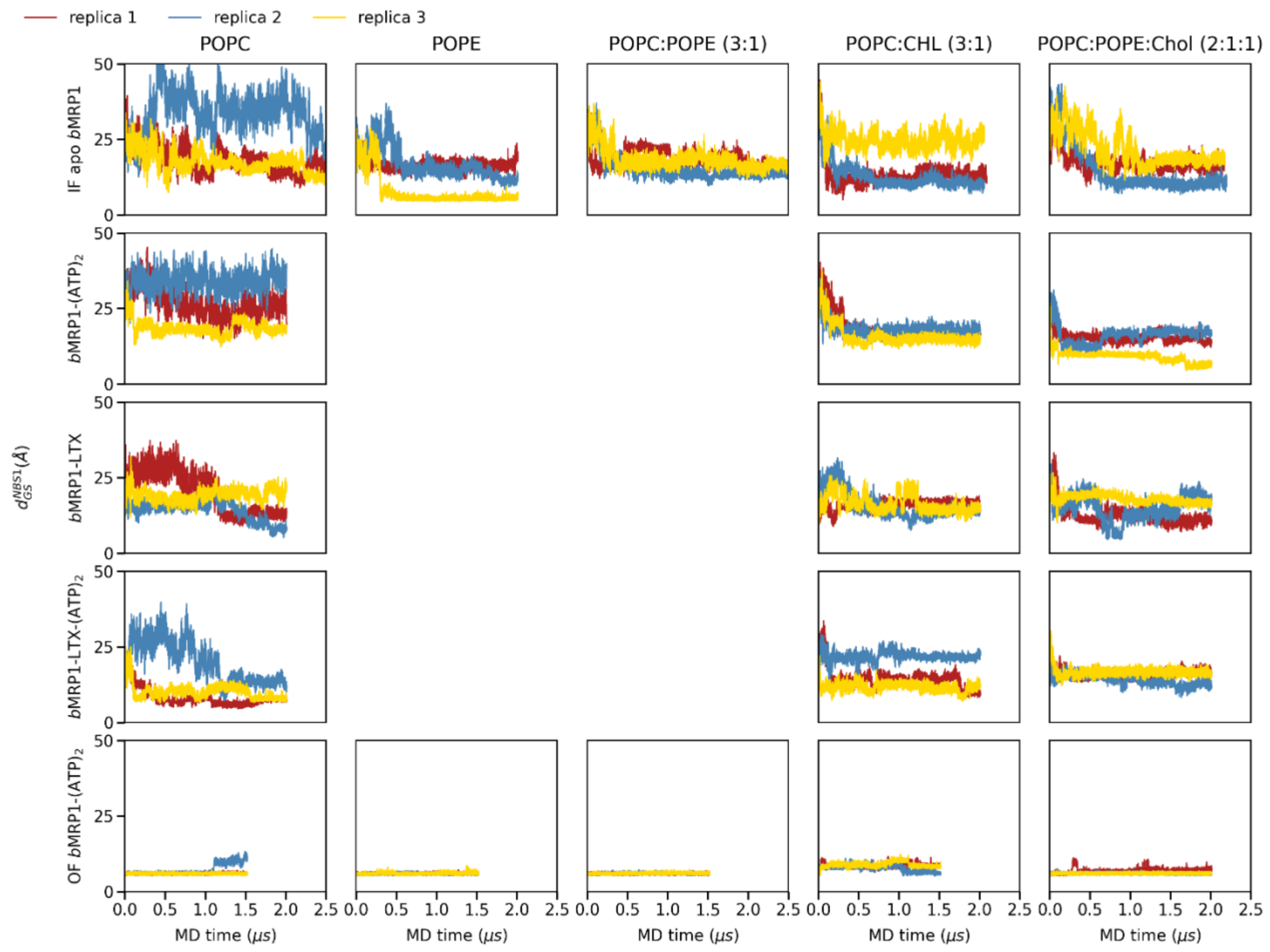

Supplementary Figure 7. Gly681-Ser1430 (d_{GS}^{NBS1}) distances (Å) during the whole simulations calculated for all systems and for all different membrane compositions. Replica 1, 2 and 3 are coloured red, blue and yellow, respectively.

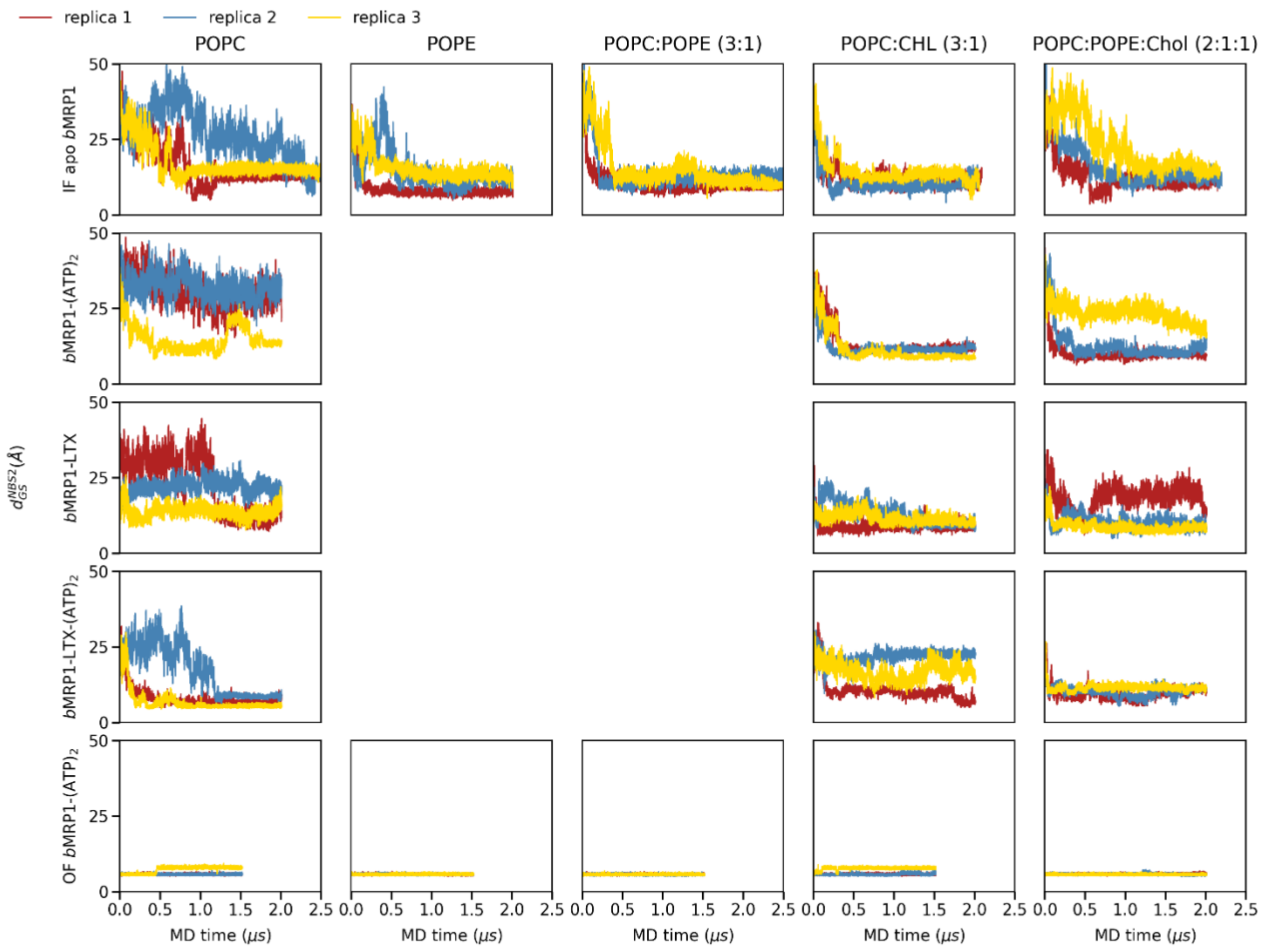

Supplementary Figure 8. Ser769-Gly1329 (d_{GS}^{NBS2}) distances (Å) during the whole simulations calculated for all systems and for all different membrane compositions. Replica 1, 2 and 3 are coloured red, blue and yellow, respectively.

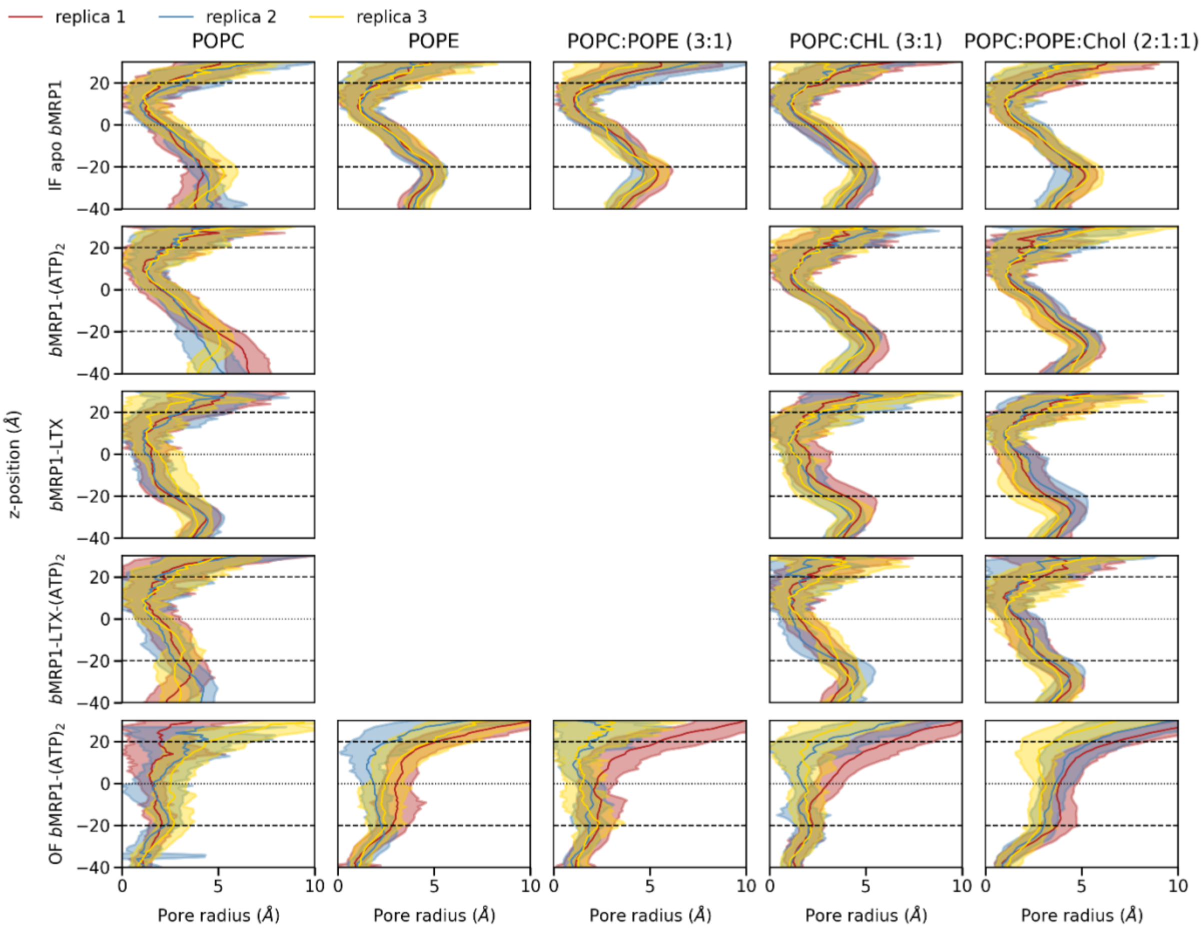

Supplementary Figure 9. Calculated z-dependent pore radii for all systems investigated in the present study. Replica 1, 2 and 3 are coloured red, blue and yellow, respectively.

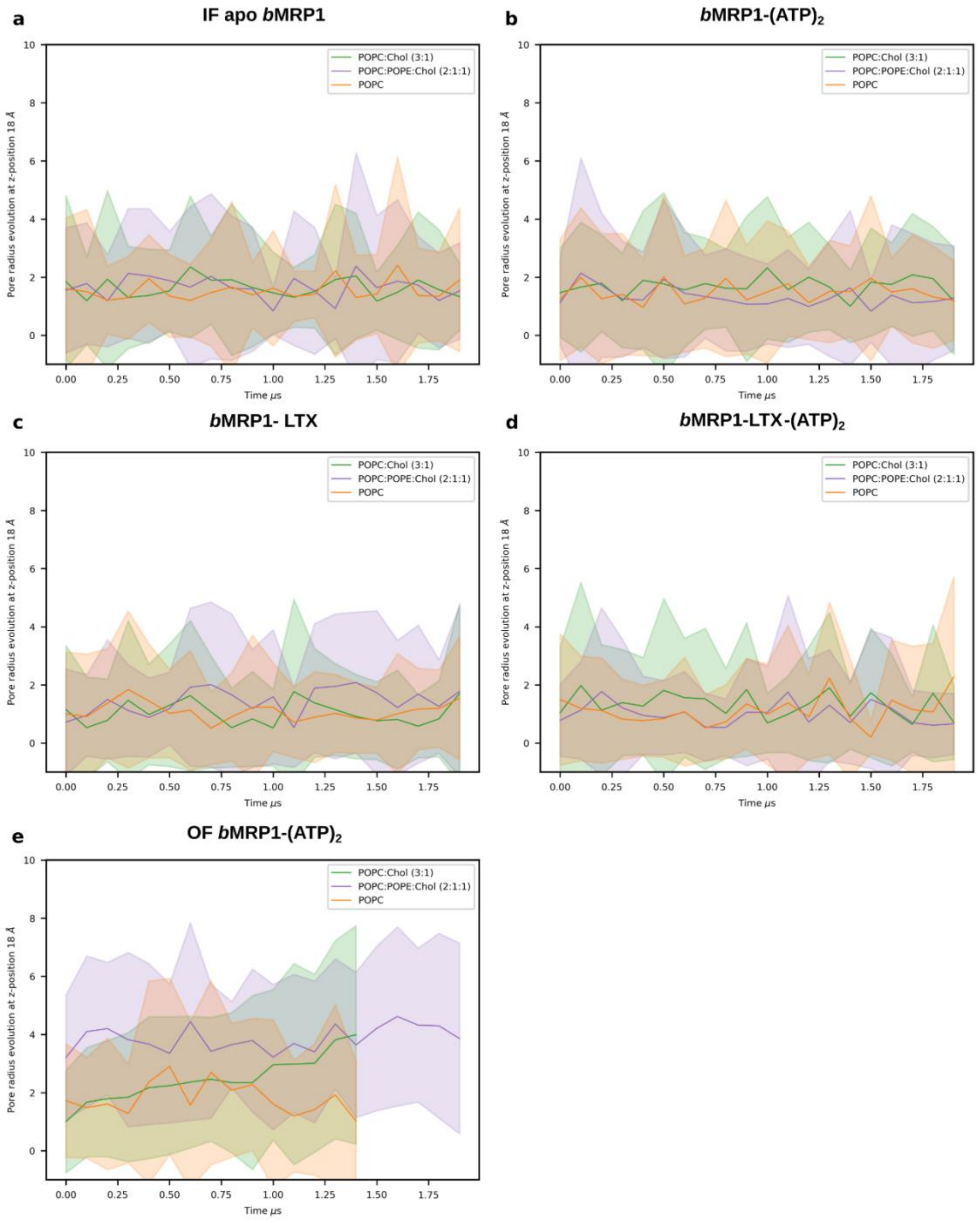

Supplementary Figure 10. Time-dependent pore radius profiles at z = 18 Å. a) IF apo *b*MRP1, **b)** *b*MRP1-$(ATP)_2$, **c)** *b*MRP1-LTX, **d)** *b*MRP1-LTX-$(ATP)_2$ and **e)** OF *b*MRP1-$(ATP)_2$.

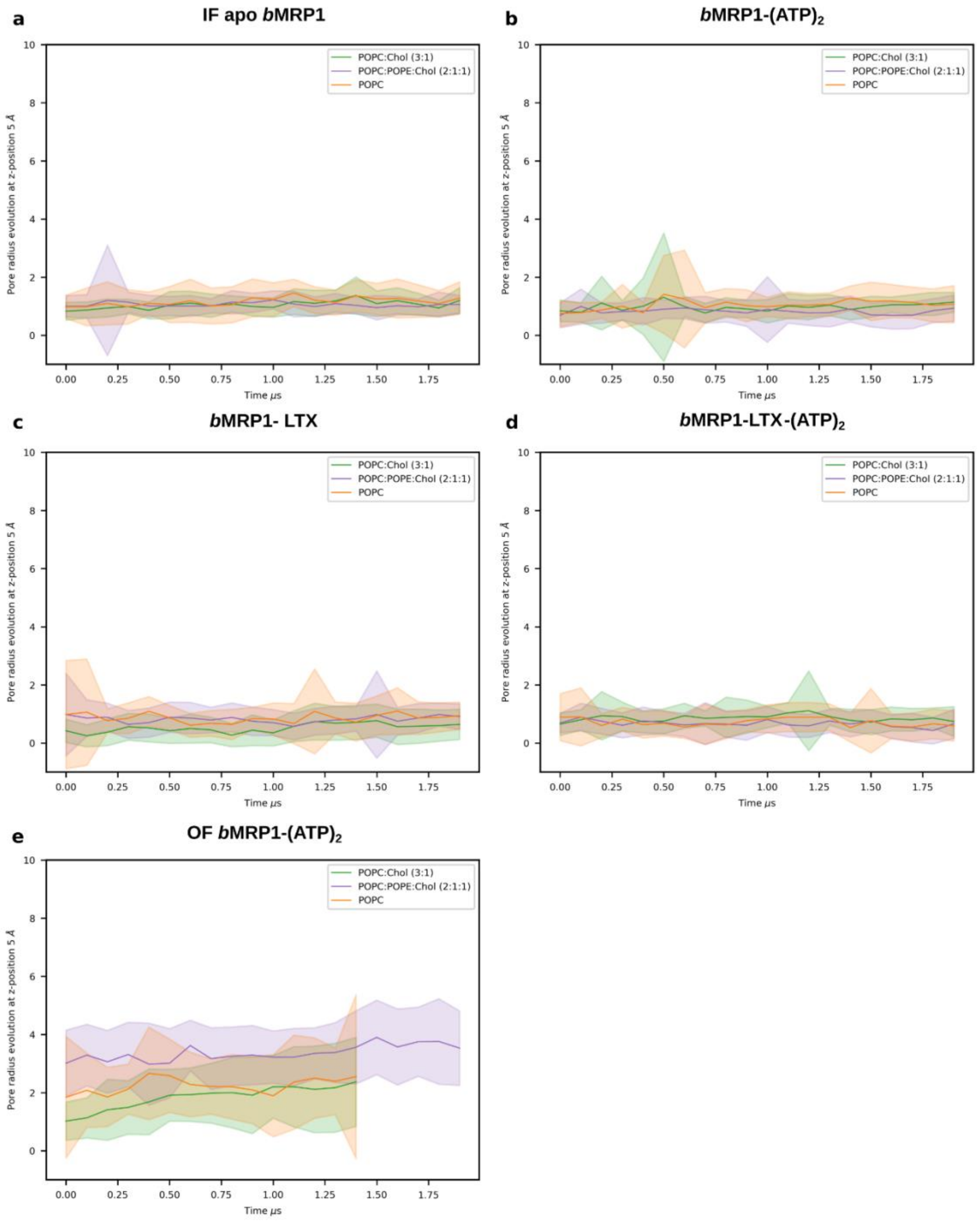

Supplementary Figure 11. Time-dependent pore radius profiles at z = 5 Å. a) IF apo *b*MRP1, **b)** *b*MRP1-(ATP)$_2$, **c)** *b*MRP1-LTX, **d)** *b*MRP1-LTX-(ATP)$_2$ and **e)** OF *b*MRP1-(ATP)$_2$.

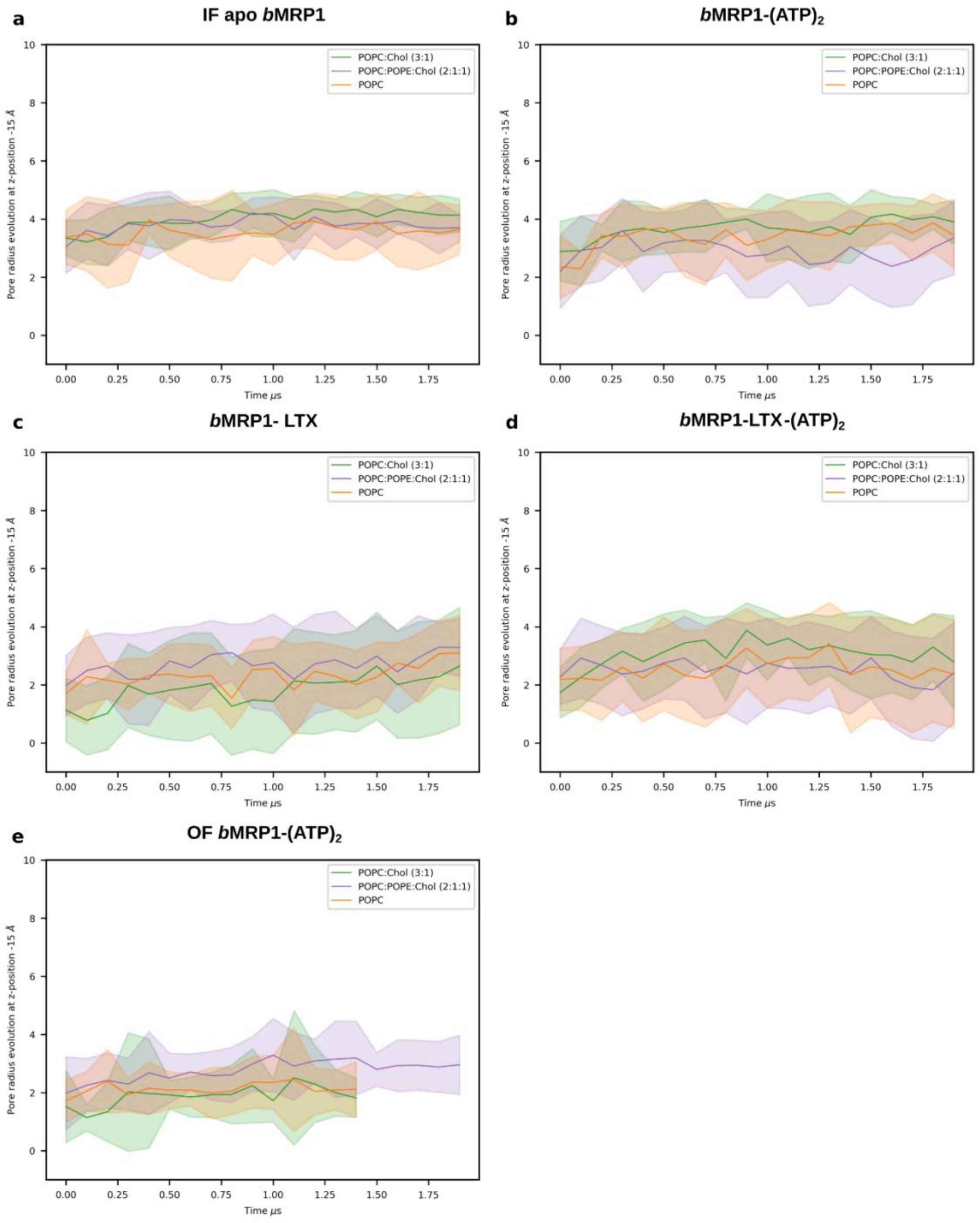

Supplementary Figure 12. Time-dependent pore radius profiles at z = -15 Å. a) IF apo *b*MRP1, **b)** *b*MRP1-$(ATP)_2$, **c)** *b*MRP1-LTX, **d)** *b*MRP1-LTX-$(ATP)_2$ and **e)** OF *b*MRP1-$(ATP)_2$.

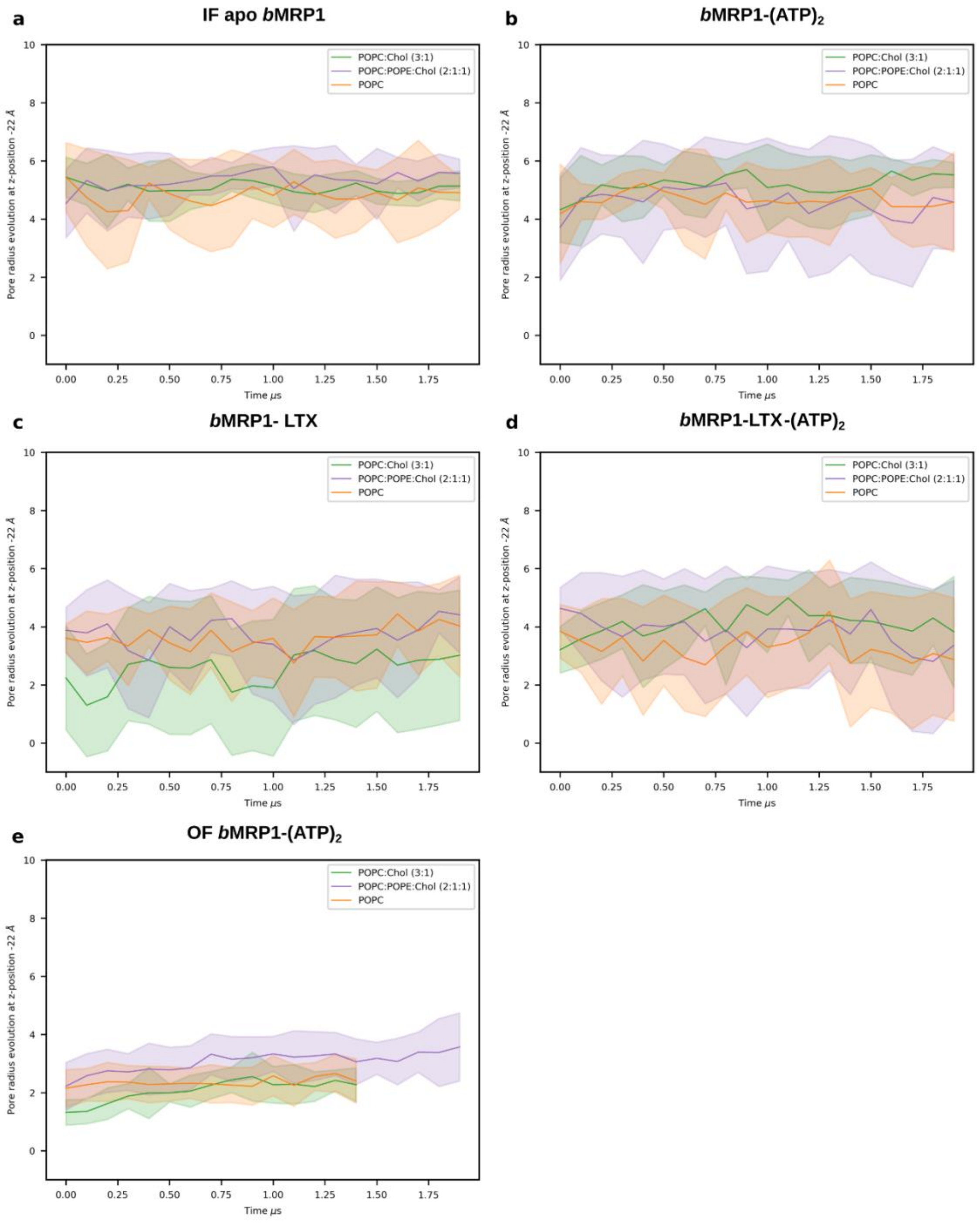

Supplementary Figure 13. Time-dependent pore radius profiles at z = -22 Å. a) IF apo *b*MRP1, **b)** *b*MRP1-(ATP)$_2$, **c)** *b*MRP1-LTX, **d)** *b*MRP1-LTX-(ATP)$_2$ and **e)** OF *b*MRP1-(ATP)$_2$.

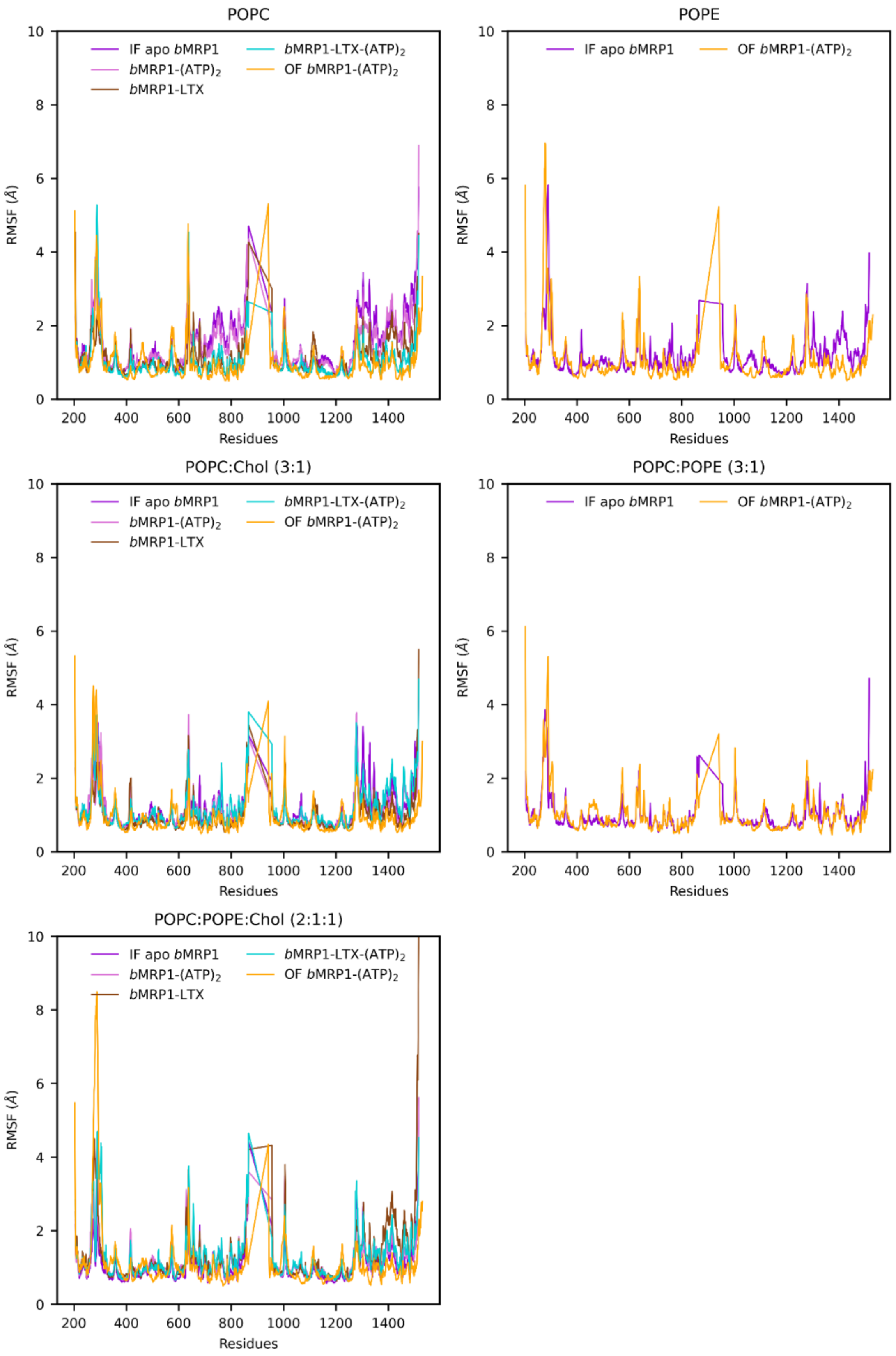

Supplementary Figure 14. Per-residue averaged Root-mean-square fluctuations (RMSF), obtained after equilibration of the different systems in different lipid bilayer compositions.

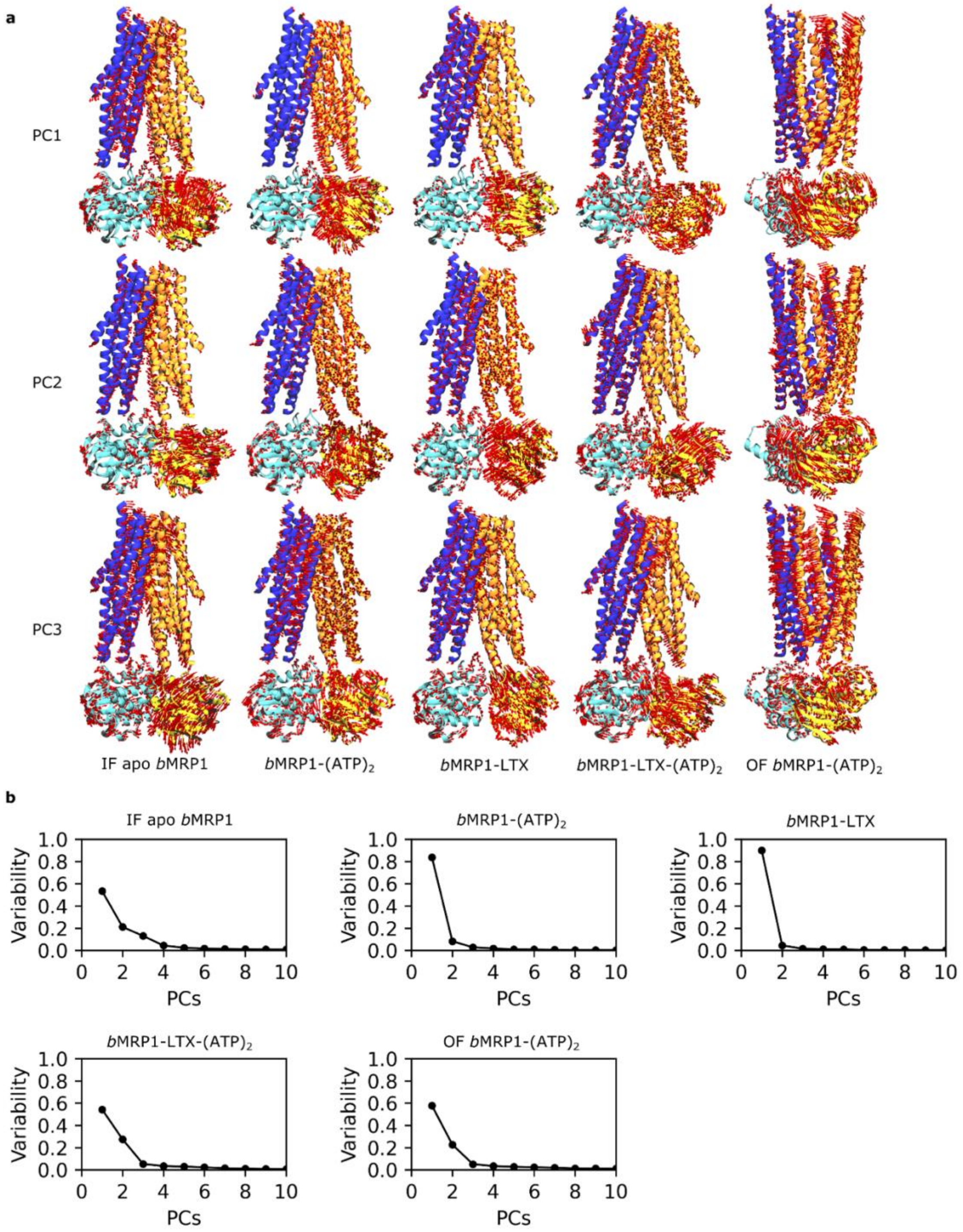

Supplementary Figure 15. Principal component analysis (PCA) of the different systems. For a given system, trajectories were aligned to an average structure obtained from the whole set of simulations. **a)** Arrows show the main movements in PC1-PC3, while on panel **b)** the variability of 10 PCs is shown.

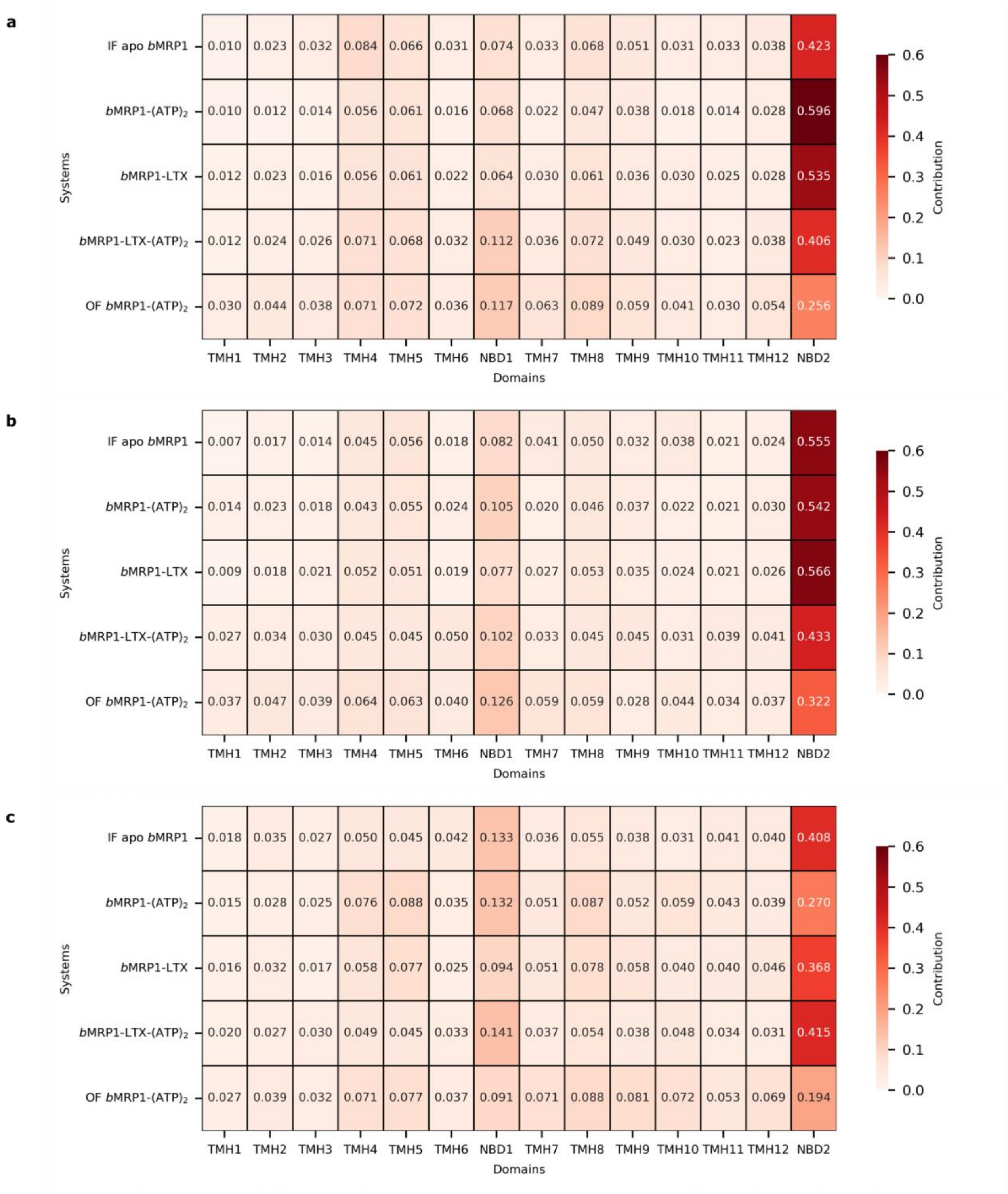

Supplementary Figure 16. Residue contributions to the three first components. a) PC1, **b)** PC2 and **c)** PC3 highlighting the major contribution of NBD2.

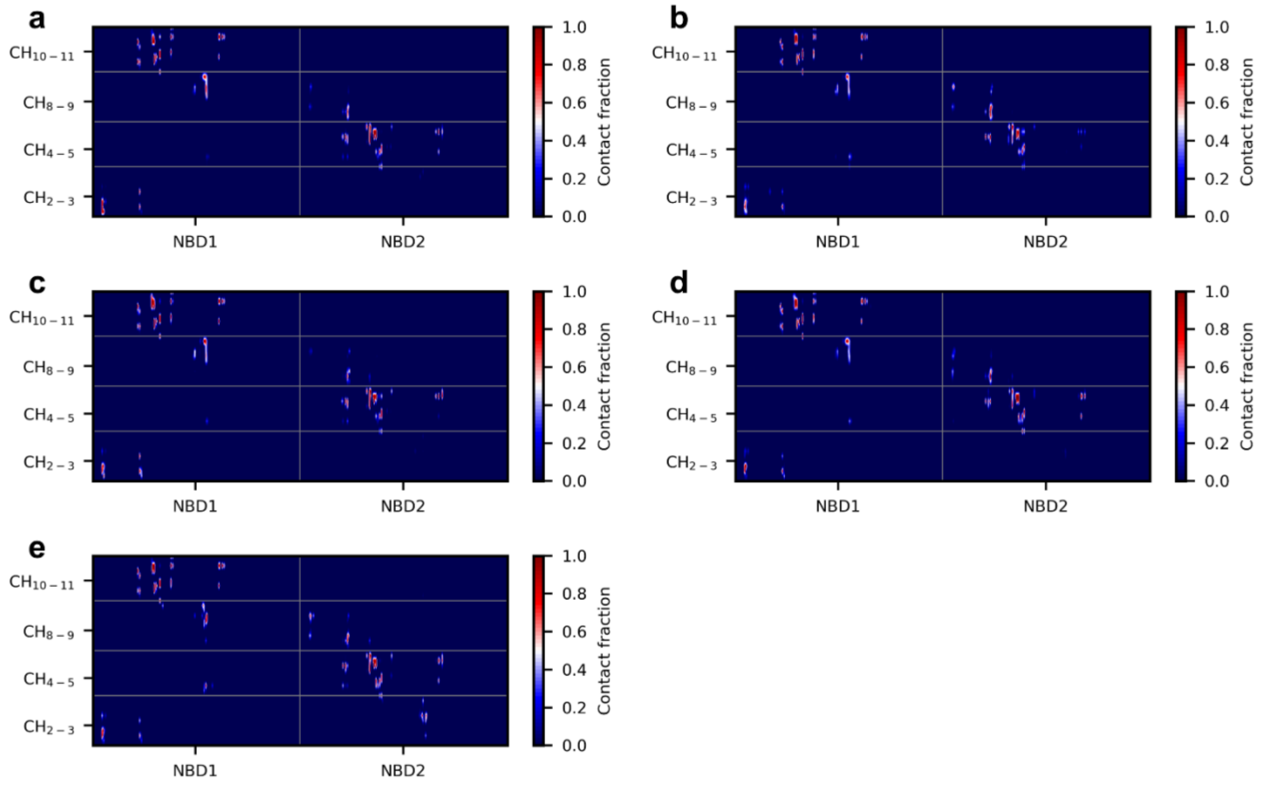

Supplementary Figure 17. Contact maps between NBDs and Coupling Helices (CHs) obtained from MD simulations performed in POPC:POPE:Chol (2:1:1). a) IF apo *b*MRP1, **b)** *b*MRP1-$(ATP)_2$, **c)** *b*MRP1-LTX, **d)** *b*MRP1-LTX-$(ATP)_2$ and **e)** OF *b*MRP1-$(ATP)_2$.

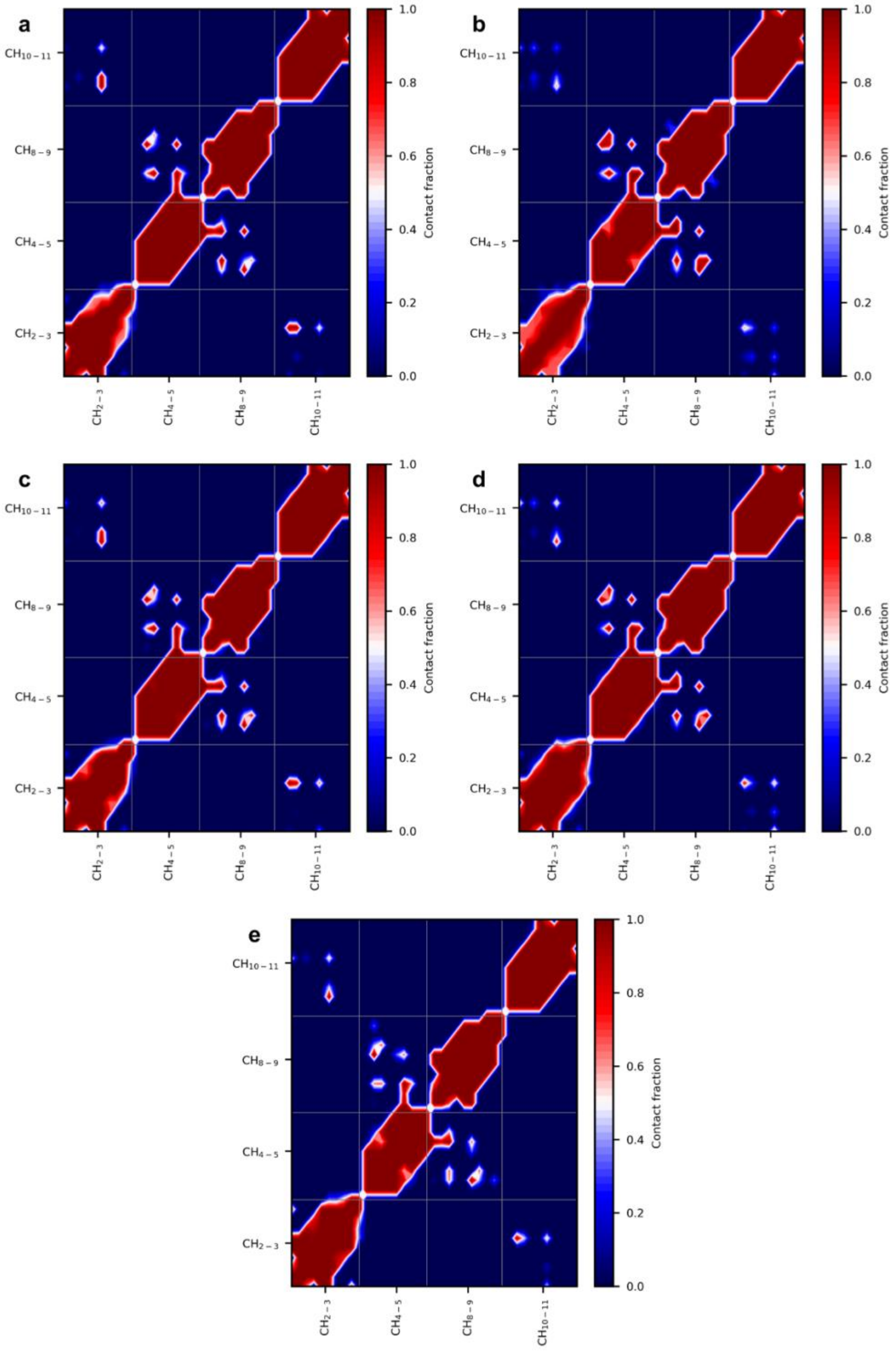

Supplementary Figure 18. Contact maps between Coupling Helices (CHs) obtained from MD simulations performed in POPC:POPE:Chol (2:1:1). a) IF apo *b*MRP1, **b)** *b*MRP1-$(ATP)_2$, **c)** *b*MRP1-LTX, **d)** *b*MRP1-LTX-$(ATP)_2$ and **e)** OF *b*MRP1-$(ATP)_2$.

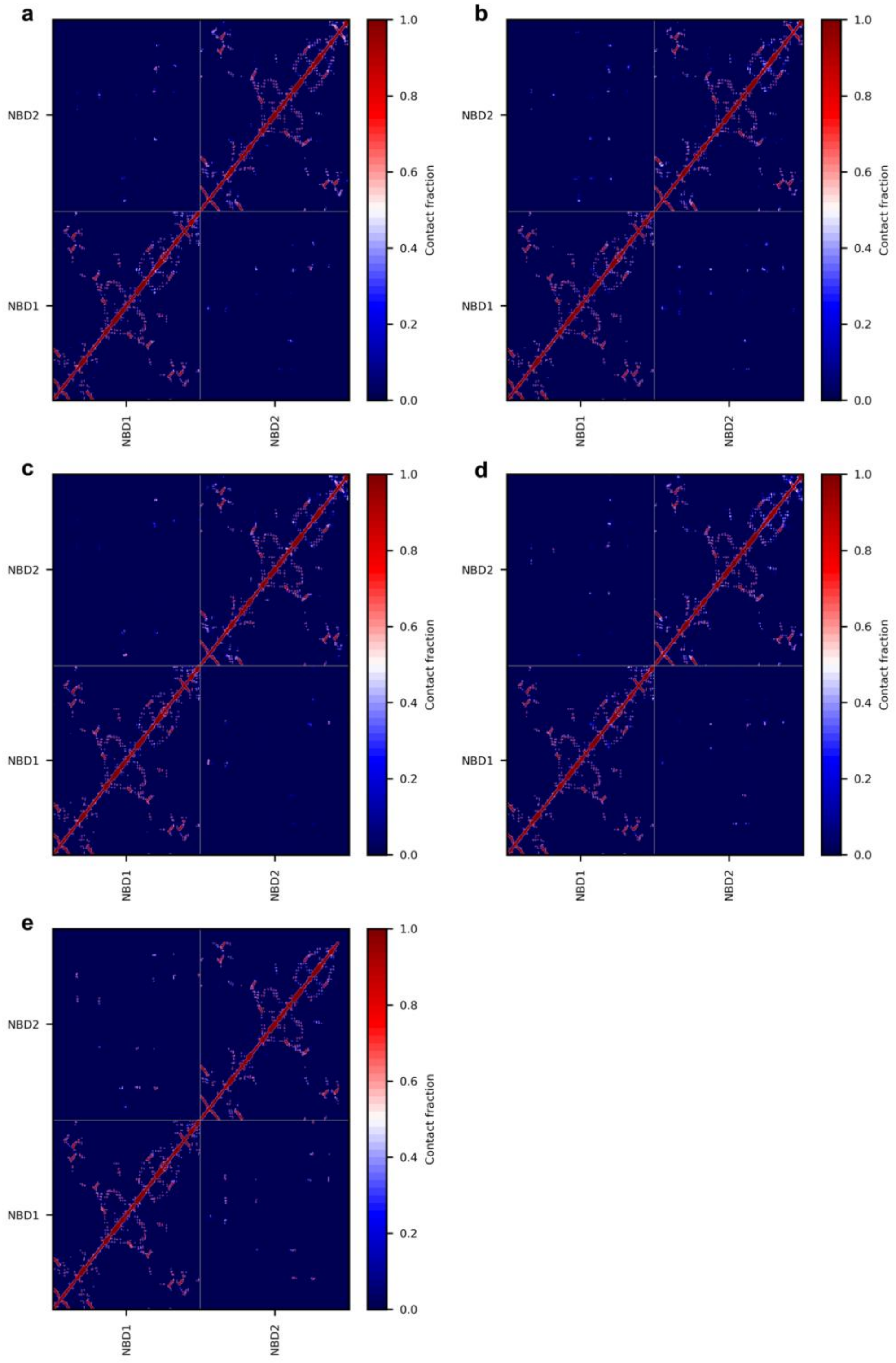

Supplementary Figure 19. Contact maps between NBDs obtained from MD simulations performed in POPC:POPE:Chol (2:1:1). a) IF apo *b*MRP1, **b)** *b*MRP1-$(ATP)_2$, **c)** *b*MRP1-LTX, **d)** *b*MRP1-LTX-$(ATP)_2$ and **e)** OF *b*MRP1-$(ATP)_2$.

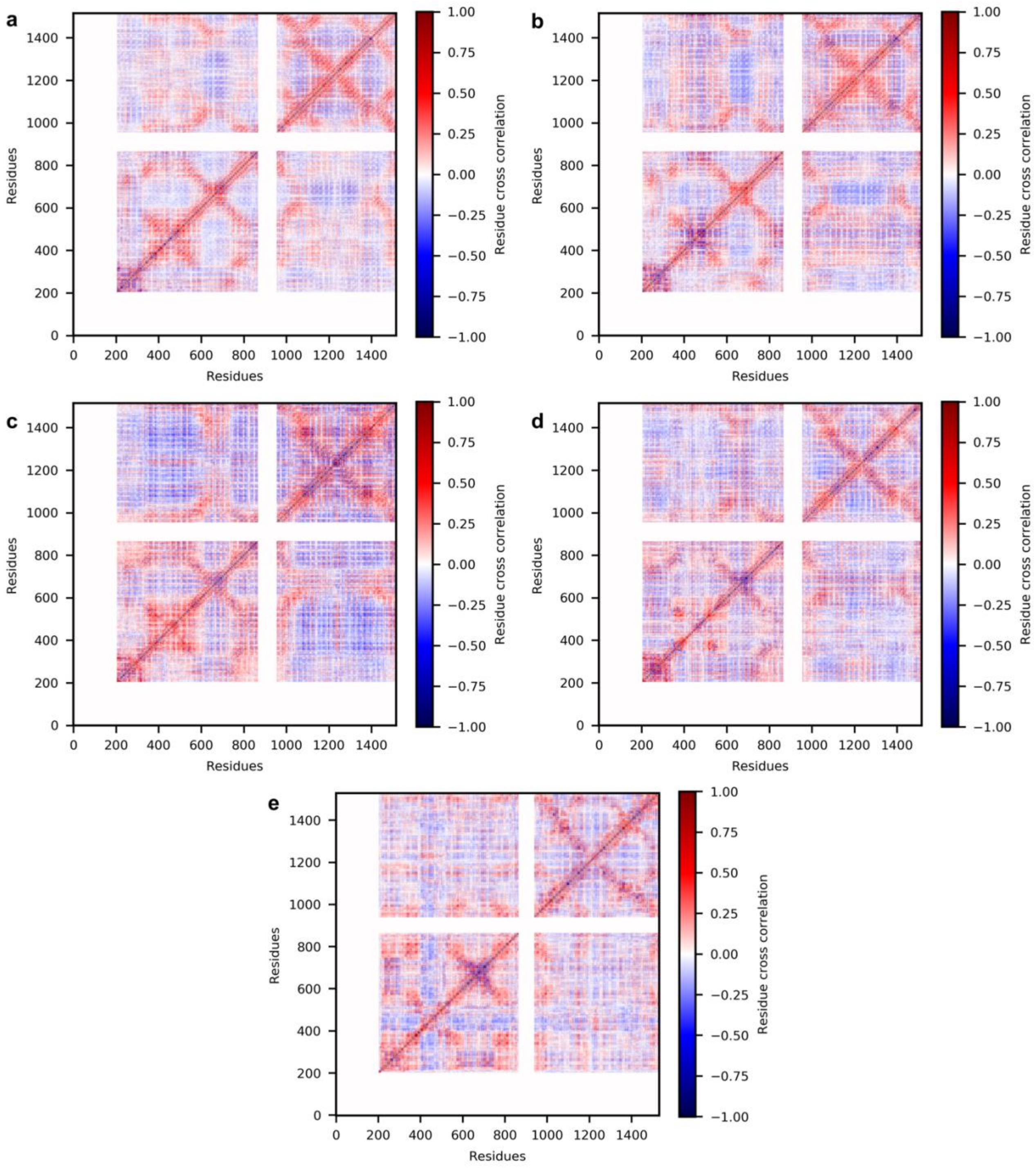

Supplementary Figure 20. Overall dynamic cross-correlation matrices from MD simulations performed in POPC:POPE:Chol (2:1:1). a) IF apo *b*MRP1, **b)** *b*MRP1-$(ATP)_2$, **c)** *b*MRP1-LTX, **d)** *b*MRP1-LTX-$(ATP)_2$ and **e)** OF *b*MRP1-$(ATP)_2$.

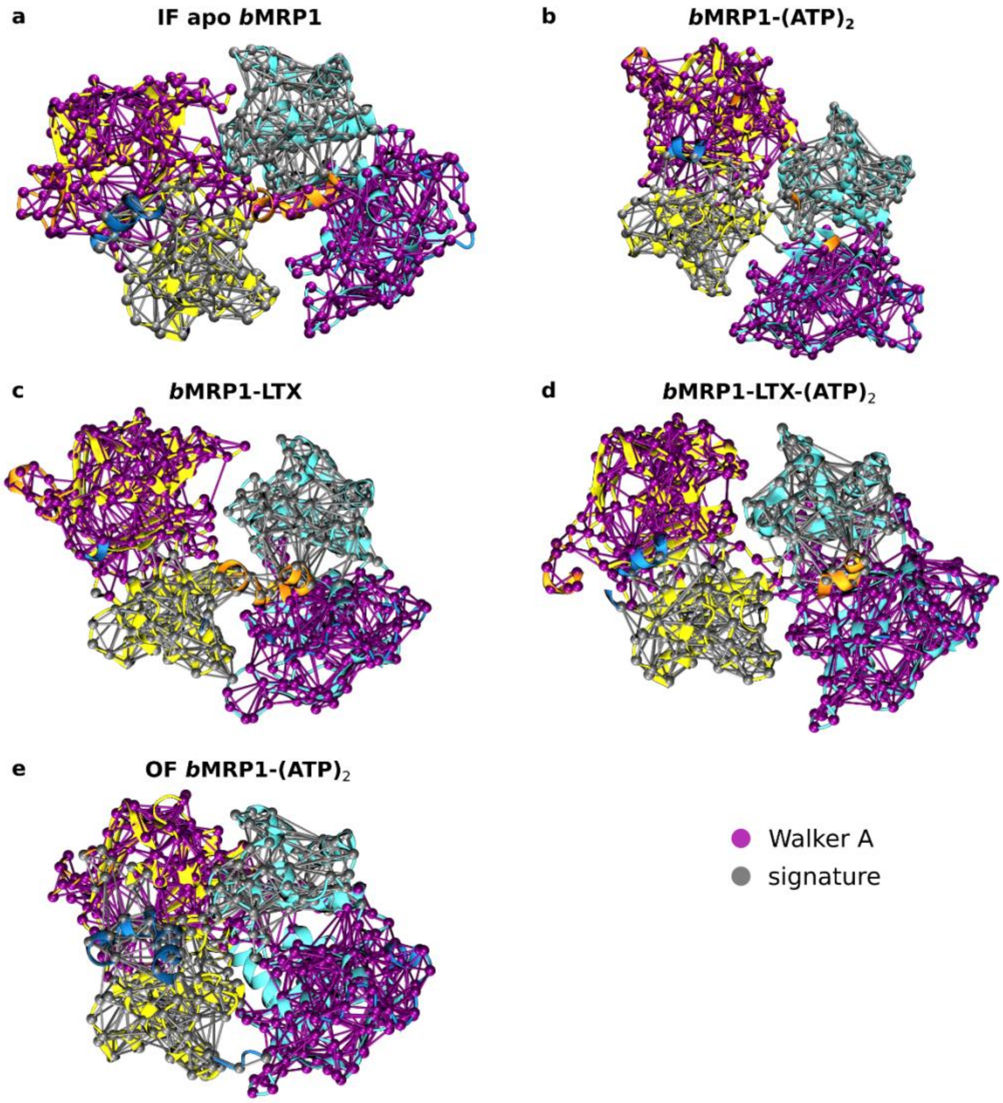

Supplementary Figure 21. Representative NBD communities from Network Analyses calculated from MD simulations performed in POPC:POPE:Chol (2:1:1) simulations. a) IF apo *b*MRP1, **b)** *b*MRP1-(ATP)$_2$, **c)** *b*MRP1-LTX, **d)** *b*MRP1-LTX-(ATP)$_2$ and **e)** OF *b*MRP1-(ATP)$_2$. NBD1 and NBD2 are coloured yellow and cyan, respectively. Orange and blue colours show the TMH parts involved in the communities. The Walker A community is purple, while the signature sequence community silver. NBD dimers are shown from upper view.

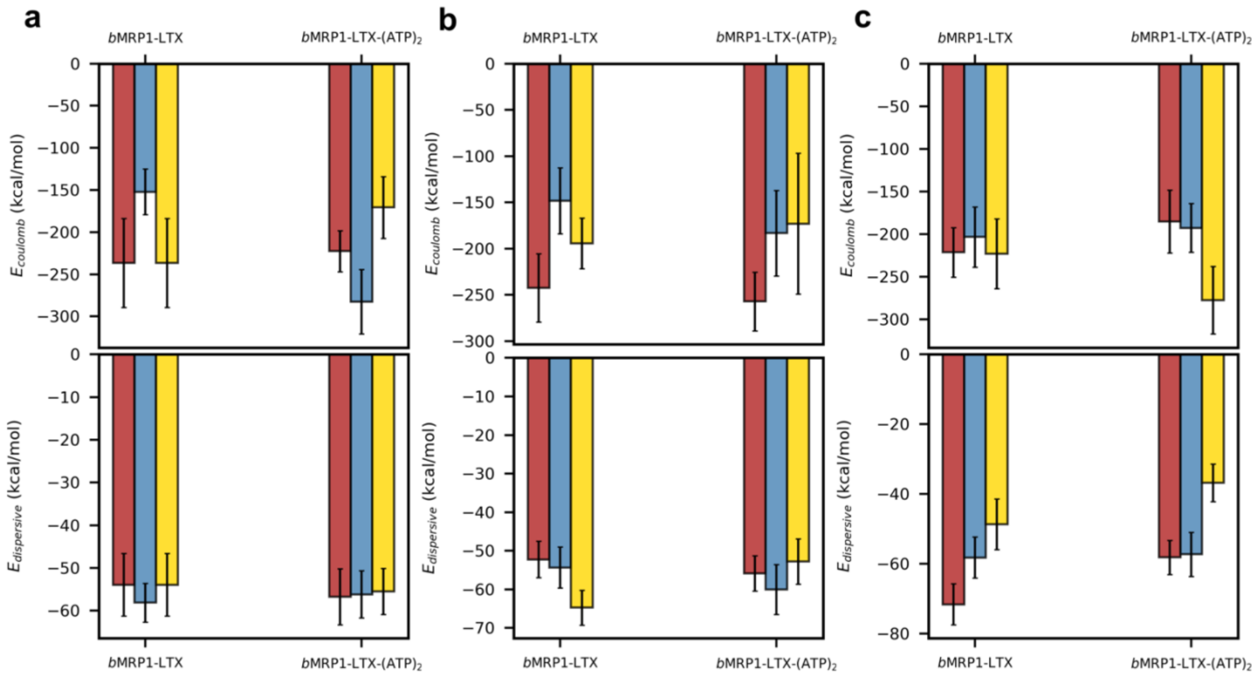

Supplementary Figure 22. Non-covalent interaction energies between LTX and *b*MRP1 extracted from Coulomb (top) and van der Waals (bottom) potentials obtained from MD simulations performed in a) POPC:POPE:Chol (2:1:1), **b)** POPC:Chol (3:1) and **c)** POPC lipid bilayers.

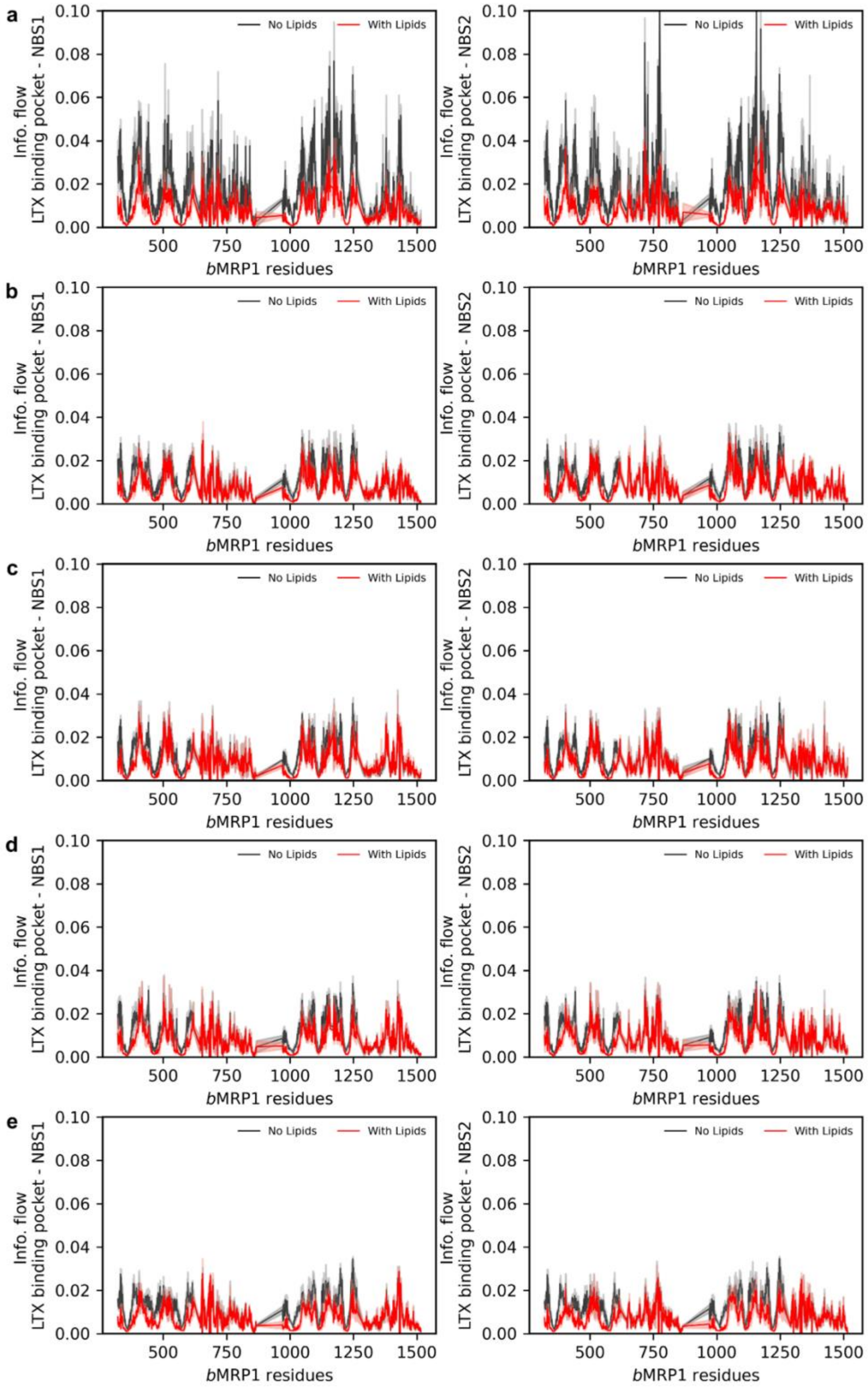

Supplementary Figure 23. Calculated per-residue betweennesses in the allosteric pathway from substrate binding pocket to NBS1 (left) and NBS2 (right). a) IF apo *b*MRP1**, b)** *b*MRP1-(ATP)$_2$, **c)** *b*MRP1-LTX, **d)** *b*MRP1-LTX-(ATP)$_2$ and **e)** OF *b*MRP1-(ATP)$_2$ systems embedded in POPC.

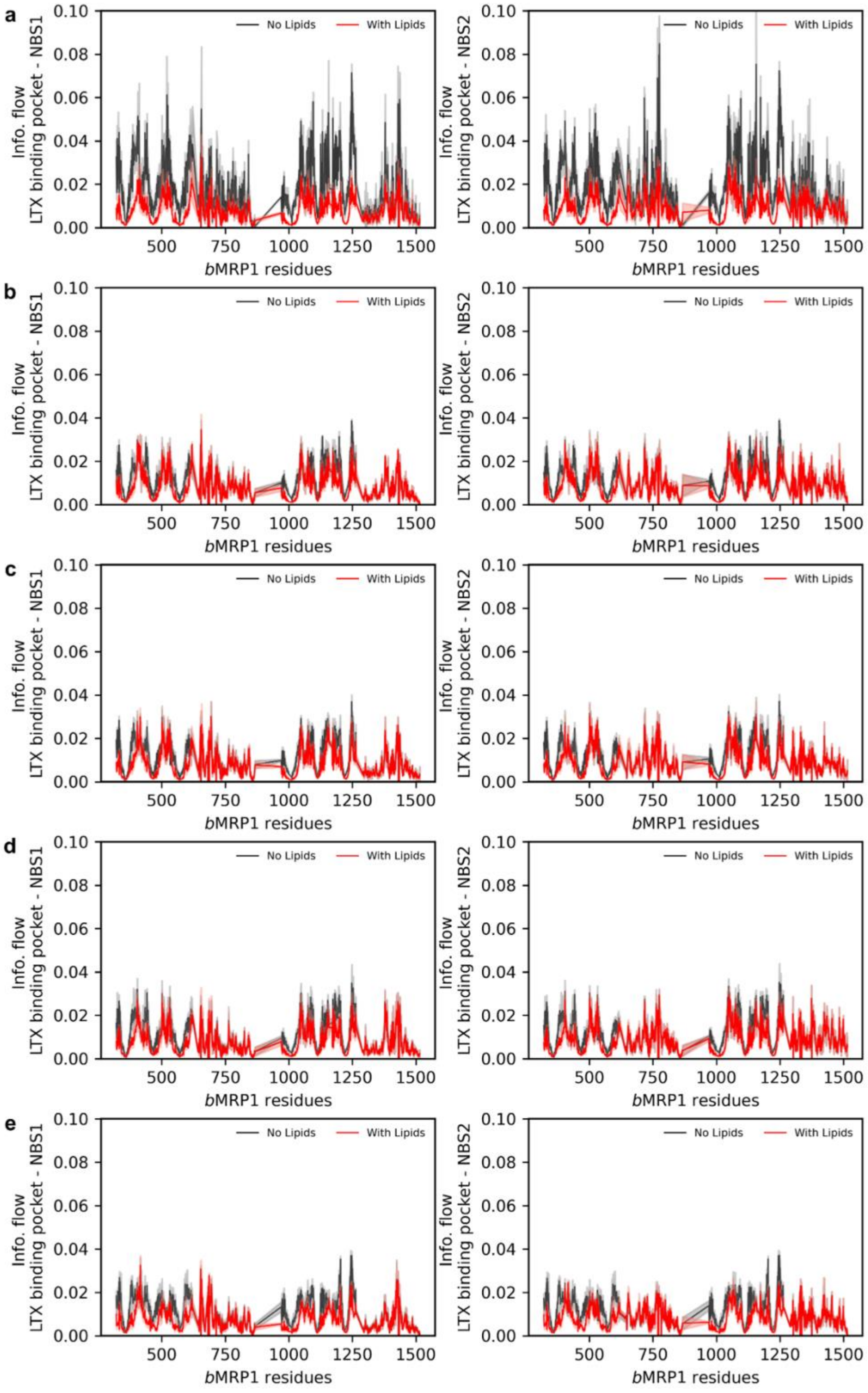

Supplementary Figure 24. Calculated per-residue betweennesses in the allosteric pathway from substrate binding pocket to NBS1 (left) and NBS2 (right). a) IF apo *b*MRP1, **b)** *b*MRP1-(ATP)$_2$, **c)** *b*MRP1-LTX, **d)** *b*MRP1-LTX-(ATP)$_2$ and **e)** OF *b*MRP1-(ATP)$_2$ systems embedded in POPC:Chol (3:1).

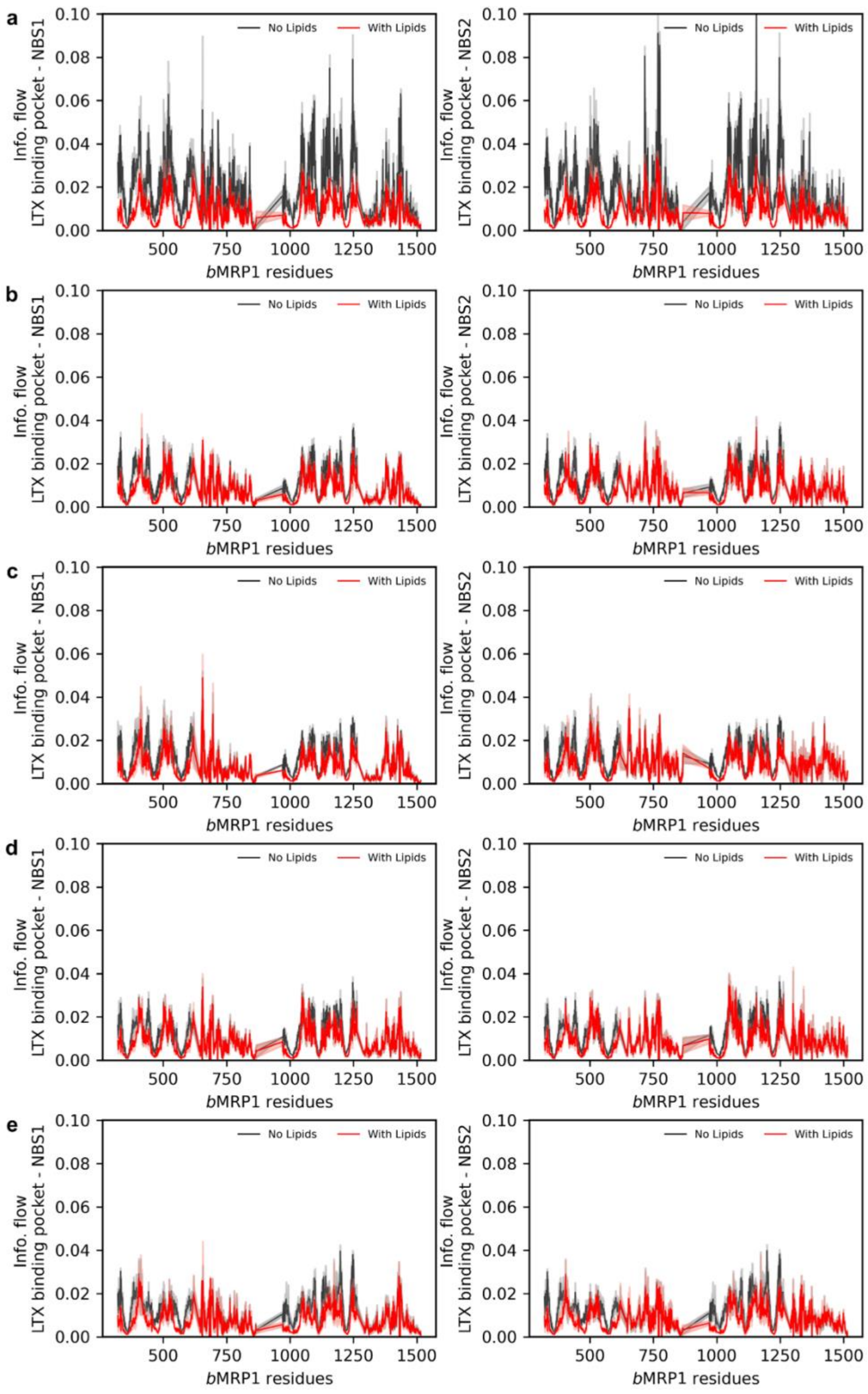

Supplementary Figure 25. Calculated per-residue betweennesses in the allosteric pathway from substrate binding pocket to NBS1 (left) and NBS2 (right). a) IF apo *b*MRP1, **b)** *b*MRP1-$(ATP)_2$, **c)** *b*MRP1-LTX, **d)** *b*MRP1-LTX-$(ATP)_2$ and **e)** OF *b*MRP1-$(ATP)_2$ systems embedded in POPC:POPE:Chol (2:1:1).

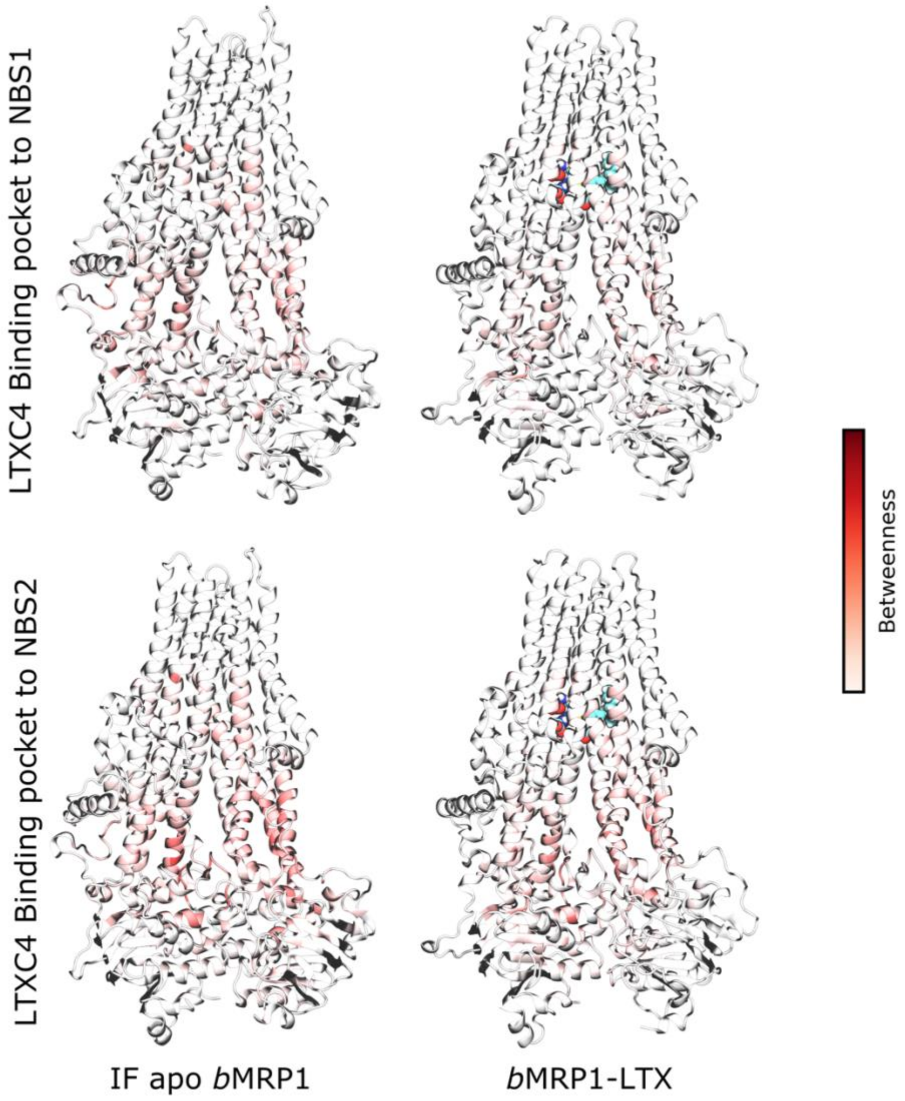

Supplementary Figure 26. Average normalized information flow through *b*MRP1 protein regarding the allosteric communication from substrate binding pocket to NBS1 and NBS2 for IF apo *b*MRP1 and *b*MRP1-LTX system in POPC:POPE:Chol (2:1:1).

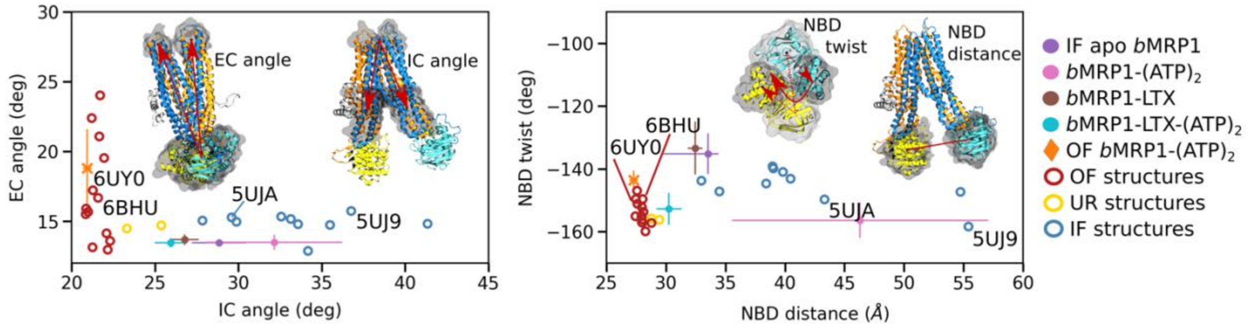

Supplementary Figure 27. ABC conformational space of trajectories in POPC compared to resolved ABC transporters.

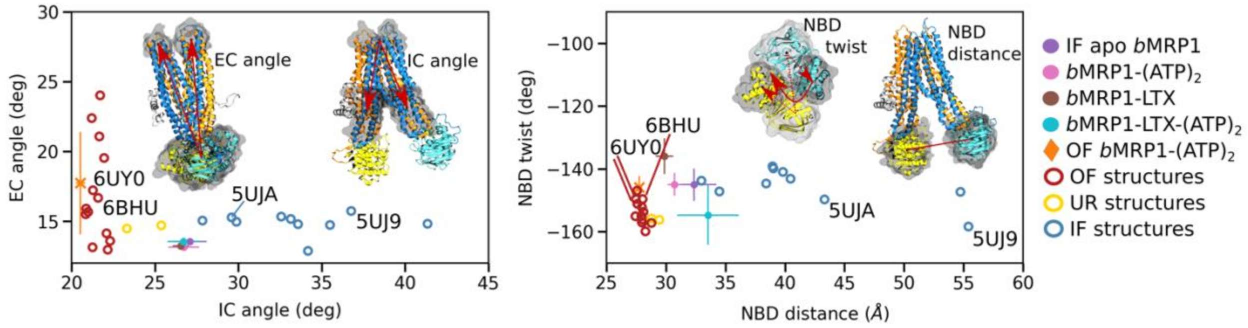

Supplementary Figure 28. ABC conformational space of trajectories in POPC:Chol (3:1) compared to resolved ABC transporters.

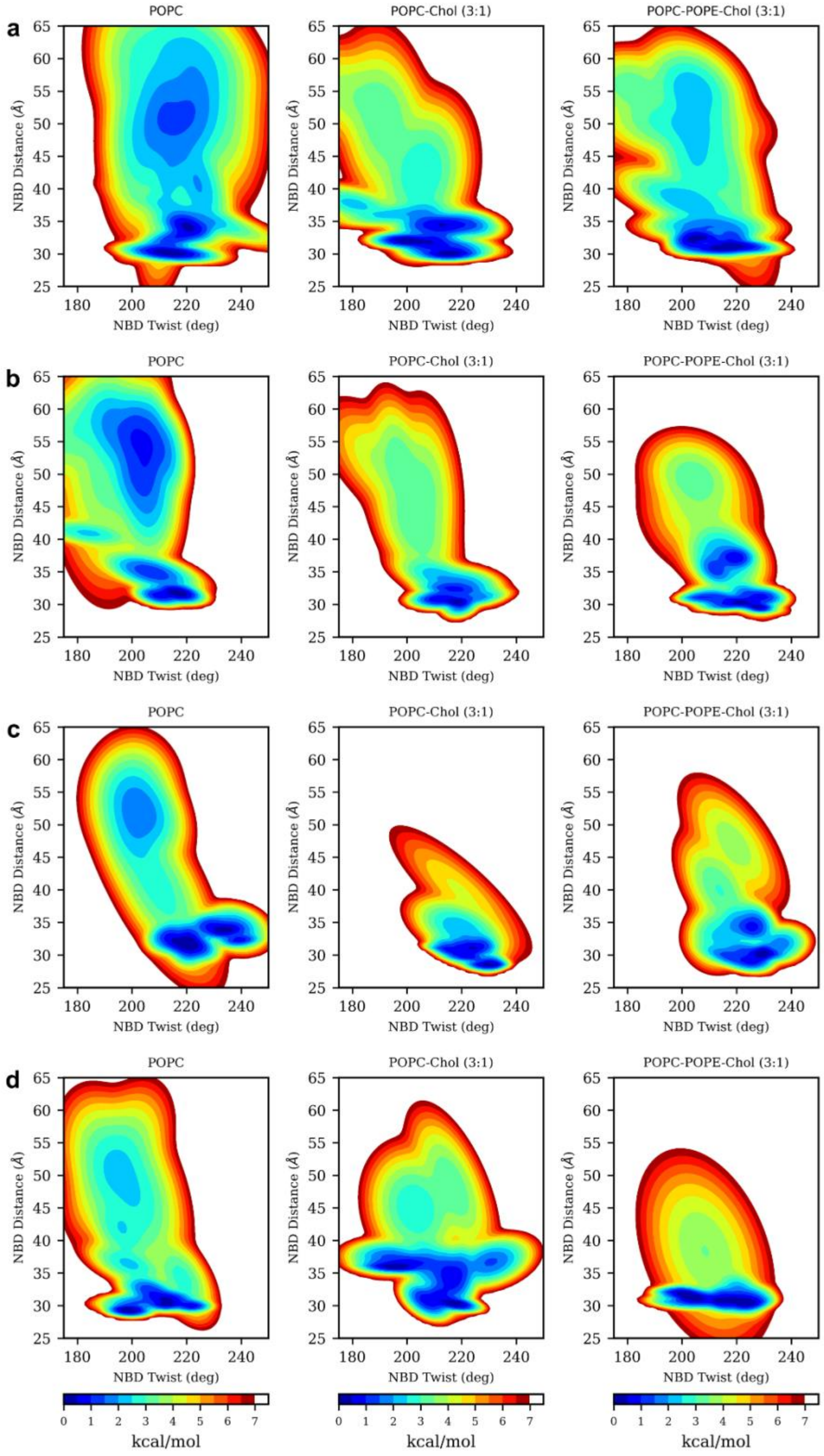

Supplementary Figure 29. Free energy landscapes according to NBD distance and NBD twist. obtained in different lipid bilayers using the InfleCS approach for IF systems, embedded in different lipid bilayers**. a)** IF apo *b*MRP1, **b)** *b*MRP1-$(ATP)_2$, **c)** *b*MRP1-LTX, **d)** *b*MRP1-LTX-$(ATP)_2$ and **e)** OF *b*MRP1-$(ATP)_2$.

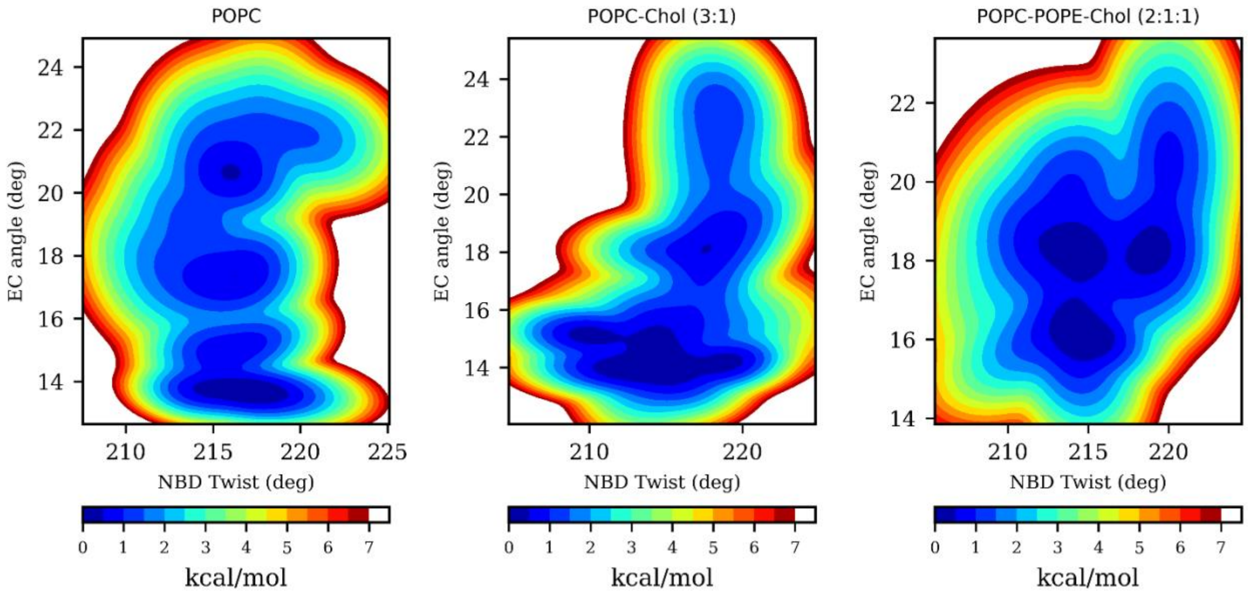

Supplementary Figure 30. Free energy landscapes according to NBD twist and EC angle obtained in different lipid bilayers using the InfleCS approach for OF *b*MRP1-(ATP)$_2$ systems.

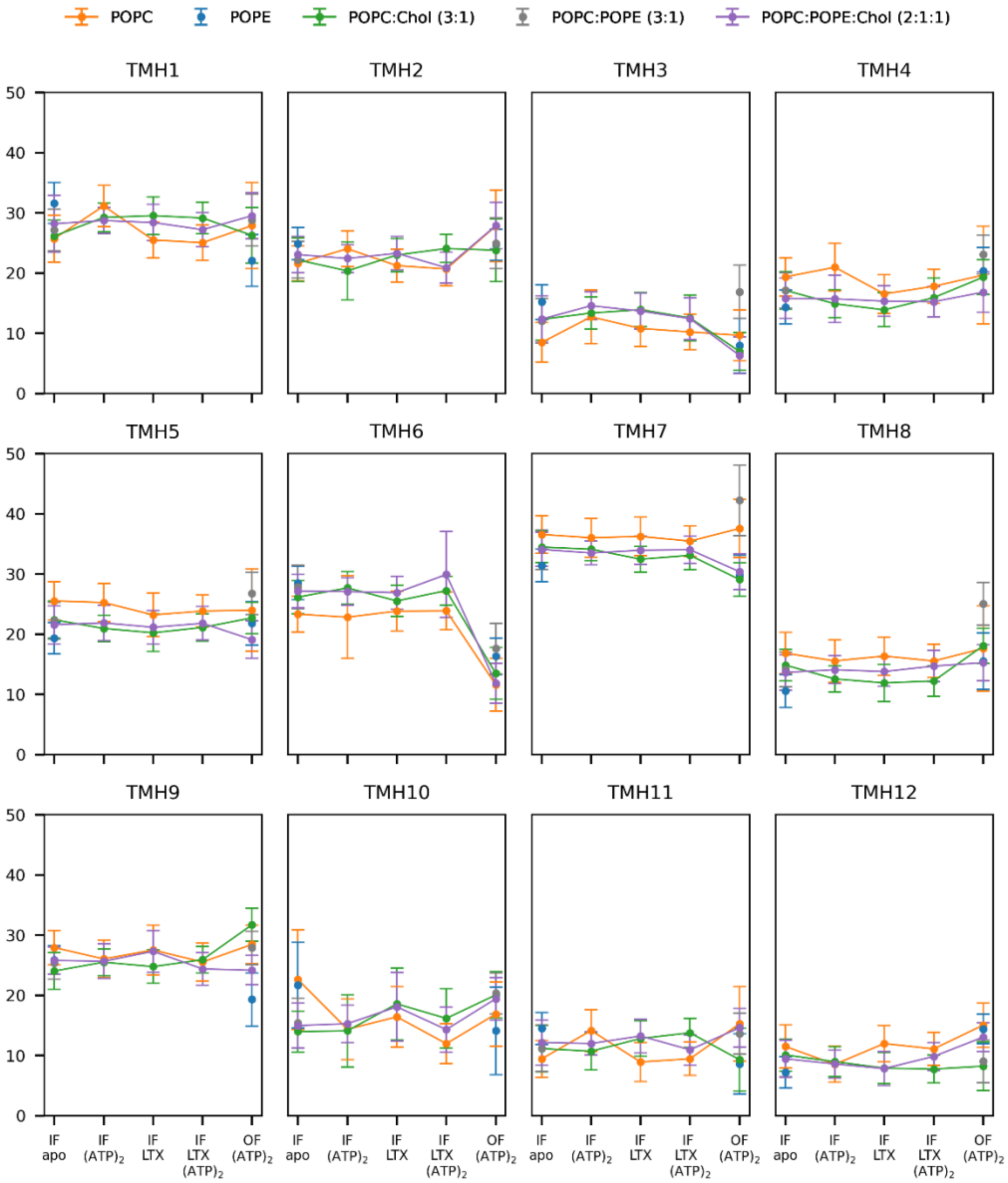

Supplementary Figure 31. Tilt angles with respect to membrane normal axis of different transmembrane helices. Since TMD_0 was not modelled in the present study, the conventional TMH1 to TMH12 labelling for type I ABC transporter was used.

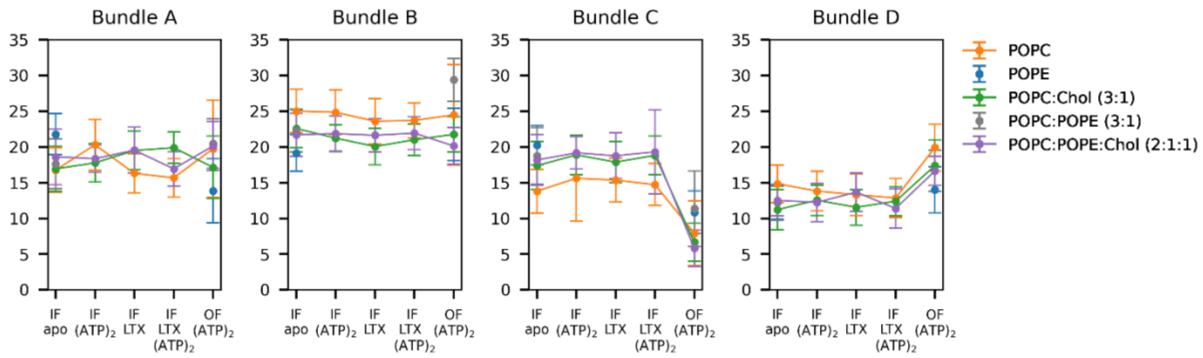

Supplementary Figure 32. Tilt angles of bundles A, B, C and D which consist in (TMH1, 2, 10 and 11), (TMH4, 5, 7 and 8), (TMH 3 and 6) and (TMH9 and 12), respectively (see main text).

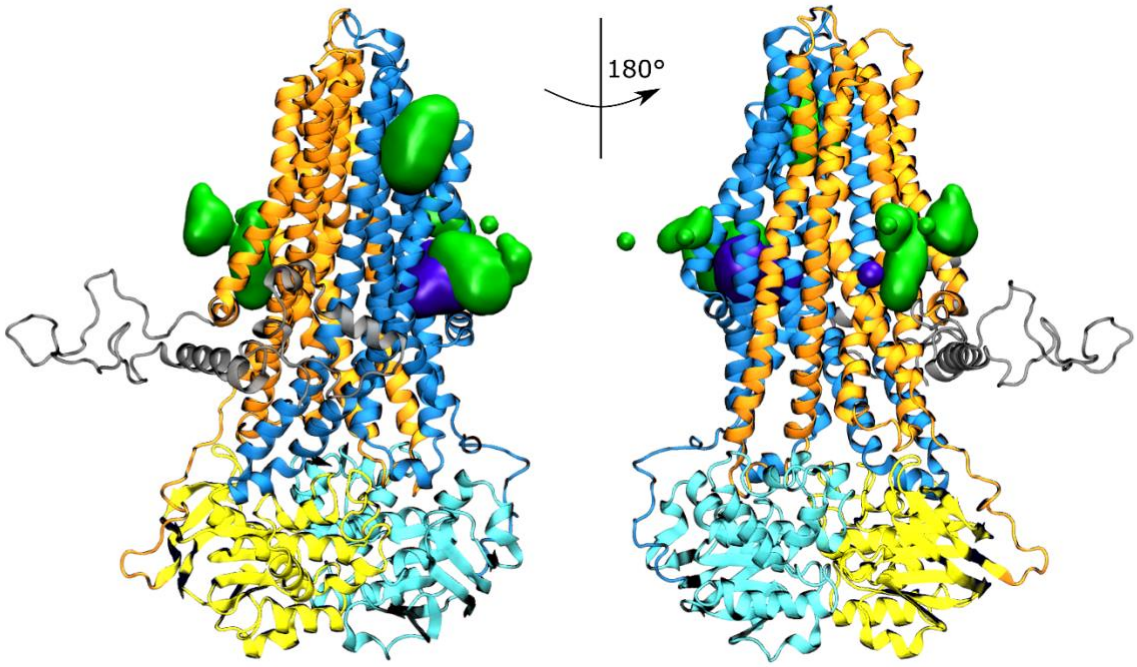

Supplementary Figure 33. Calculated binding hotspots obtained from cholesterol defined by presence likelihood higher than 50%. Cryo-EM resolved cholesterols are coloured violet, other cholesterols green.

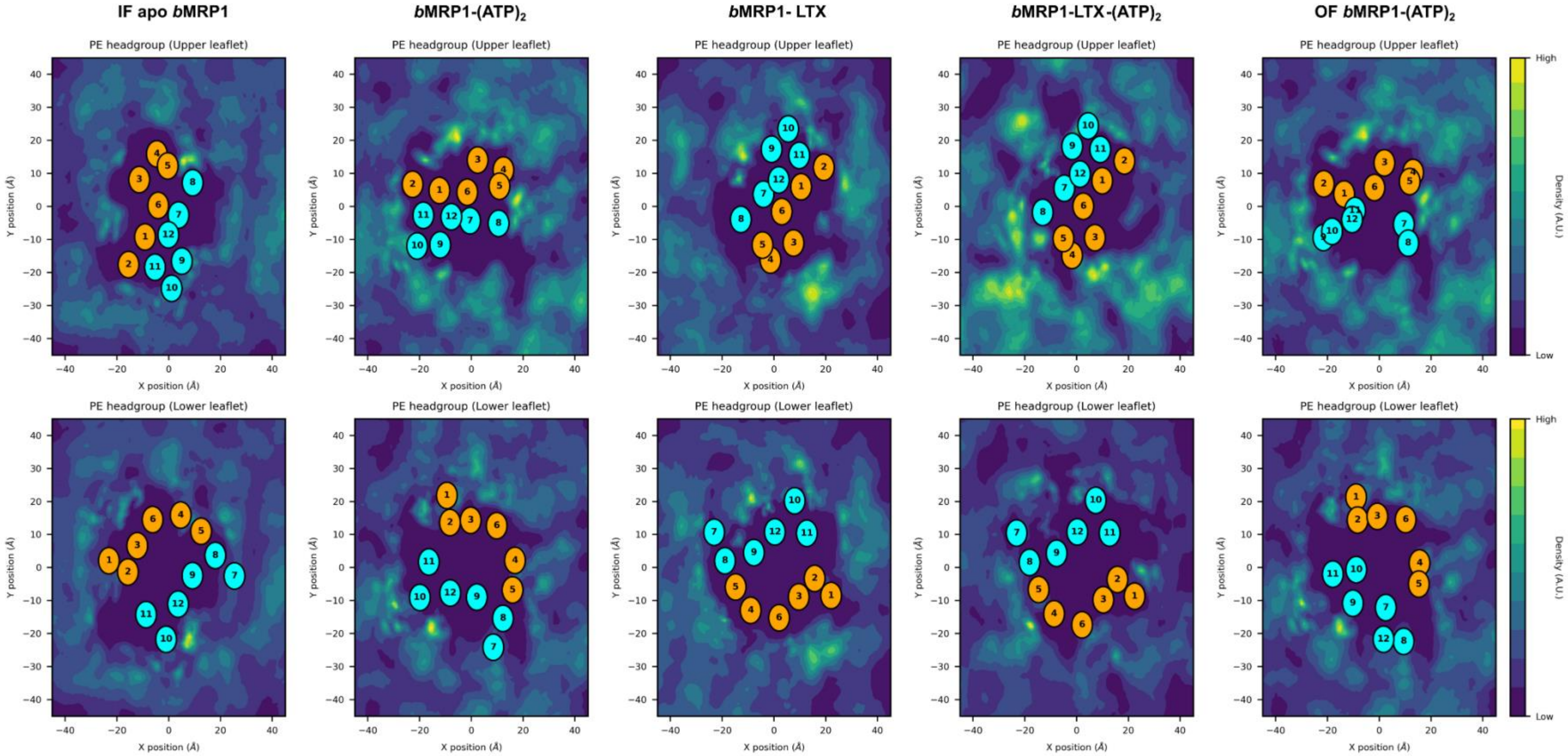

Supplementary Figure 34. PE-lipid distribution in POPC:POPE:Chol (2:1:1) lipid bilayer.

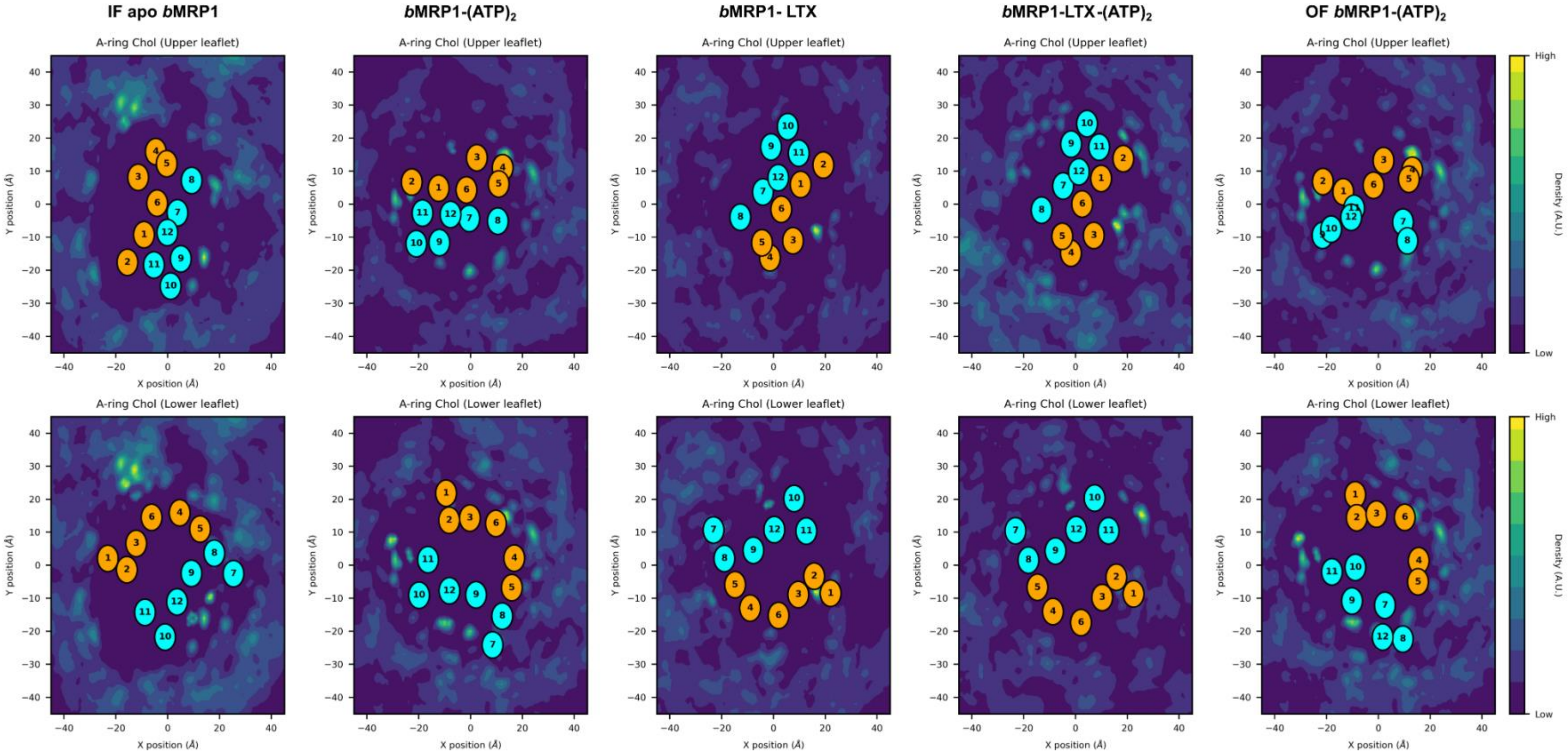

Supplementary Figure 35. Cholesterol distribution in POPC:POPE:Chol (2:1:1) lipid bilayer.

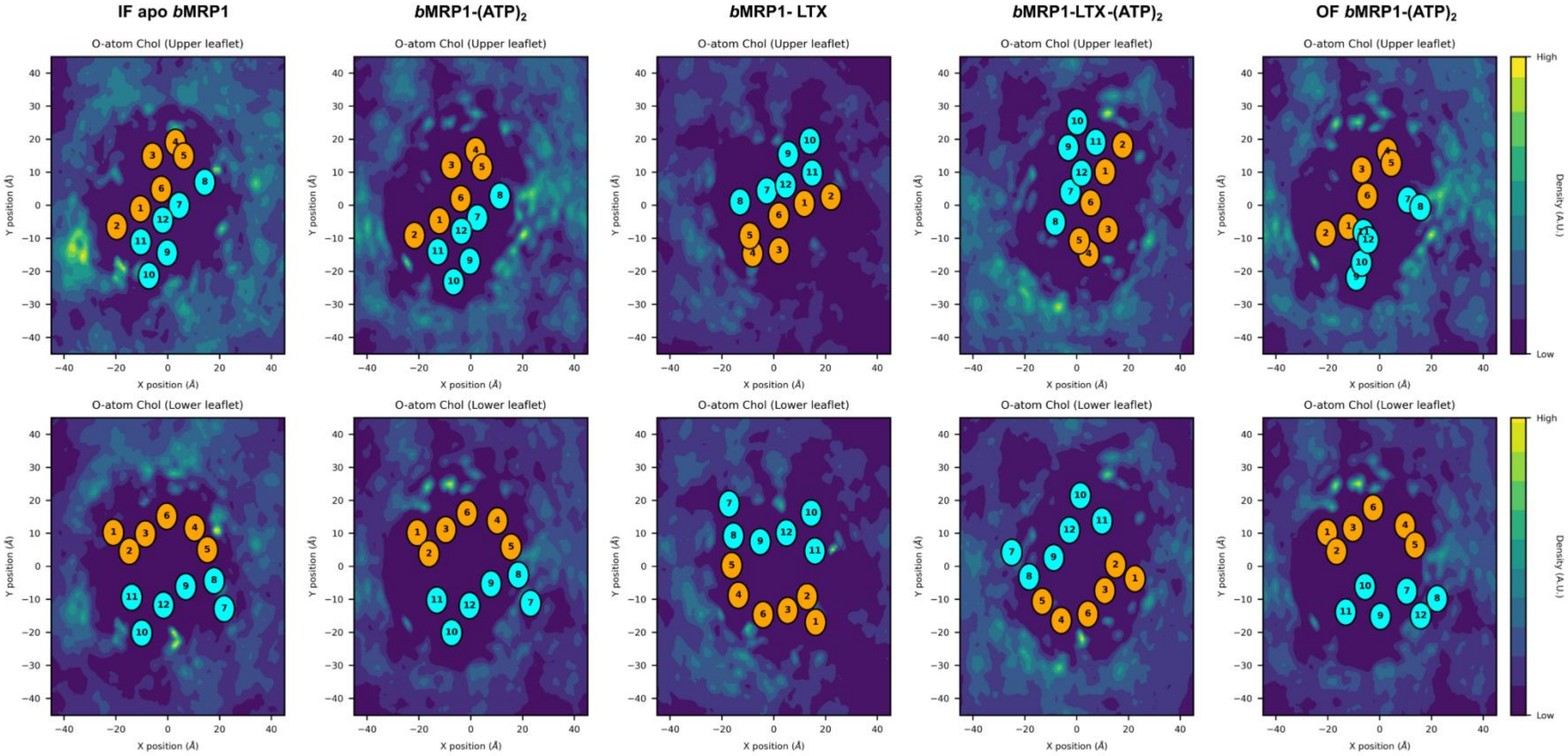

Supplementary Figure 36. Cholesterol distribution in POPC:Chol (3:1) lipid bilayer.

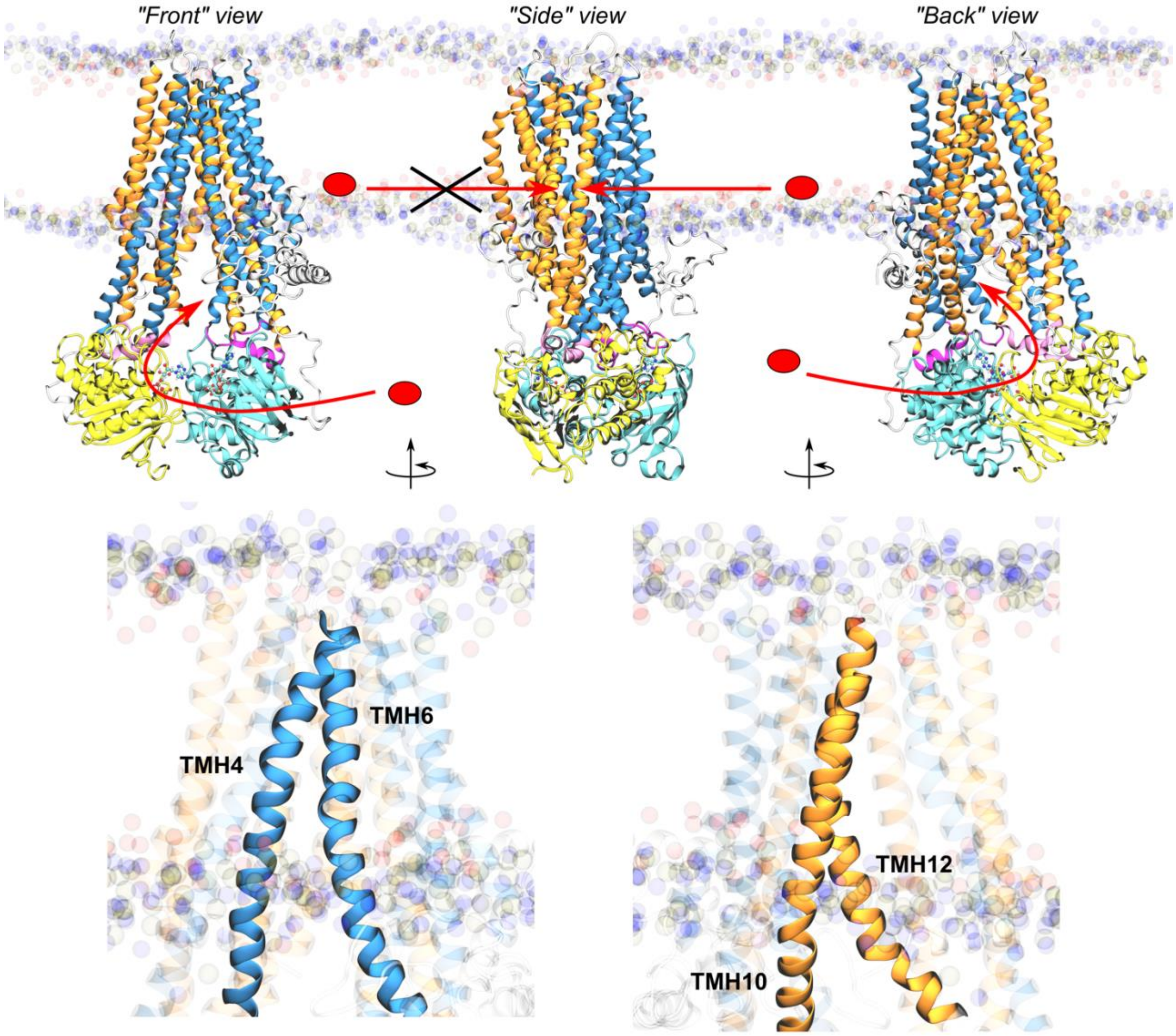

Supplementary Figure 37. Putative substrate access to *b*MRP1 binding pocket. We here propose different possible substrate access to *b*MRP1 binding pocket depending on substrate lipid bilayer partitioning. Amphiphilic compounds including charged molecules were shown to possibly partition within the high-density polar head region of lipid bilayer membrane. Our MD simulations suggest that access to TMD binding pocket may be possible between TMH4 and TMH6 while access between TMH10 and TMH12 might less likely.

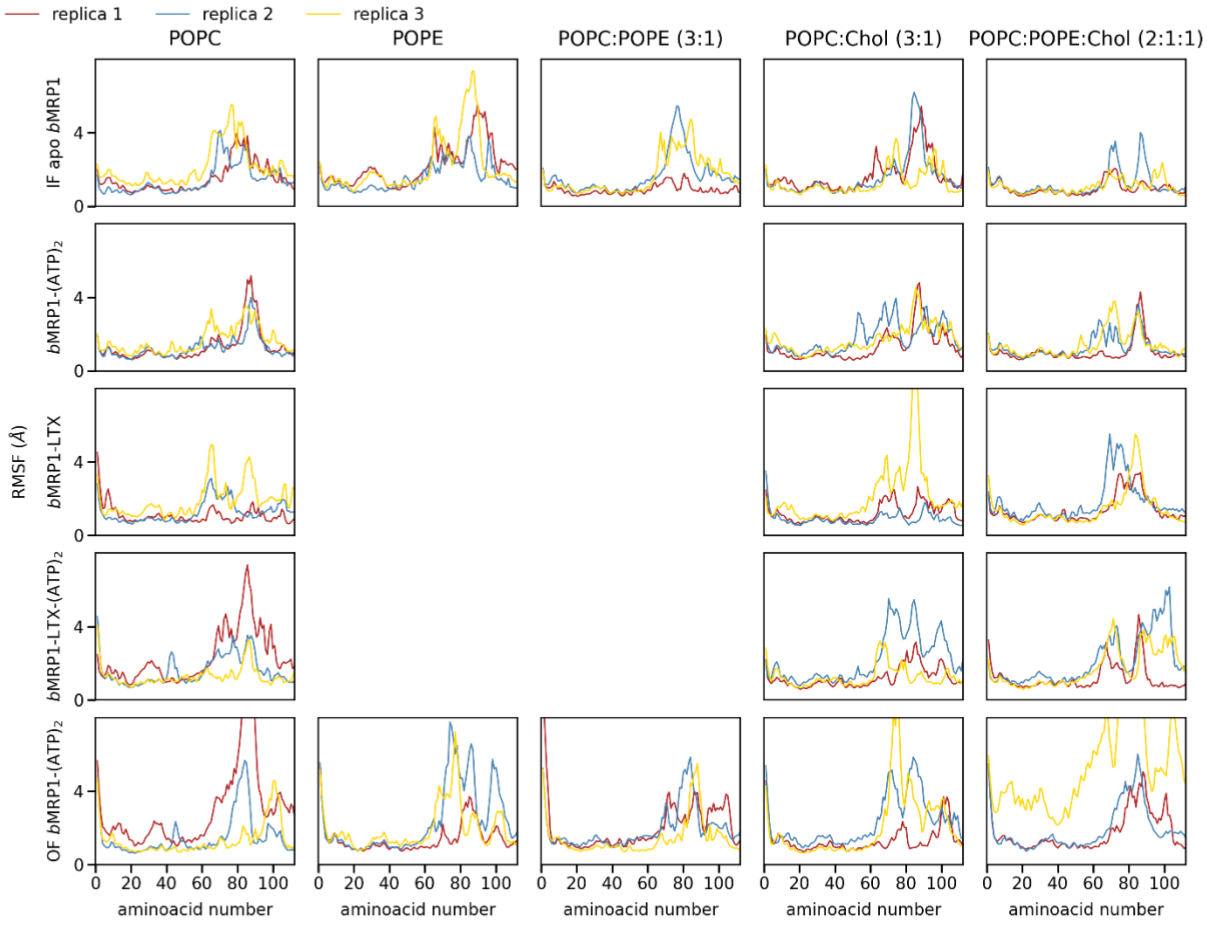

Supplementary Figure 38. Root-mean square fluctuation of L_0. The part which was modelled using the sequence and not the loop from the IF model (around 80-95) is the most flexible part in the IF models, as well.

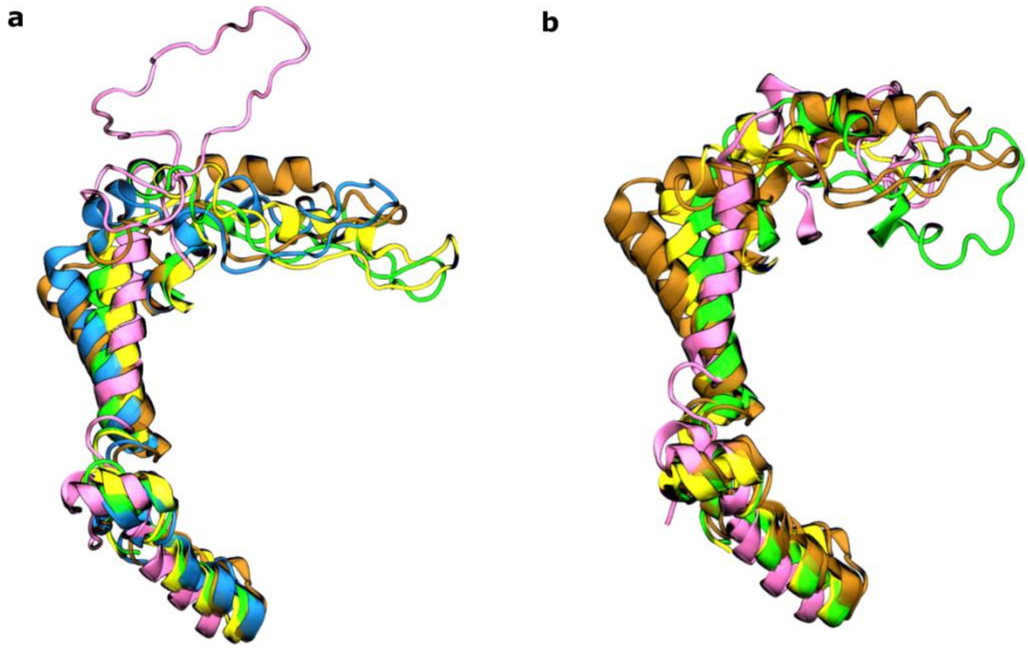

Supplementary Figure 39. L_0 structures. a) Starting frames converge to the same conformation shown by **b)** the final frames. Top view., aligned on TH1-3,6,10-11 (bundle A and C). IF apo is blue, IF ATP-bound ochre, IF LTX-bound yellow, IF LTX-ATP-bound green, and OF ATP-bound mauve/pink.

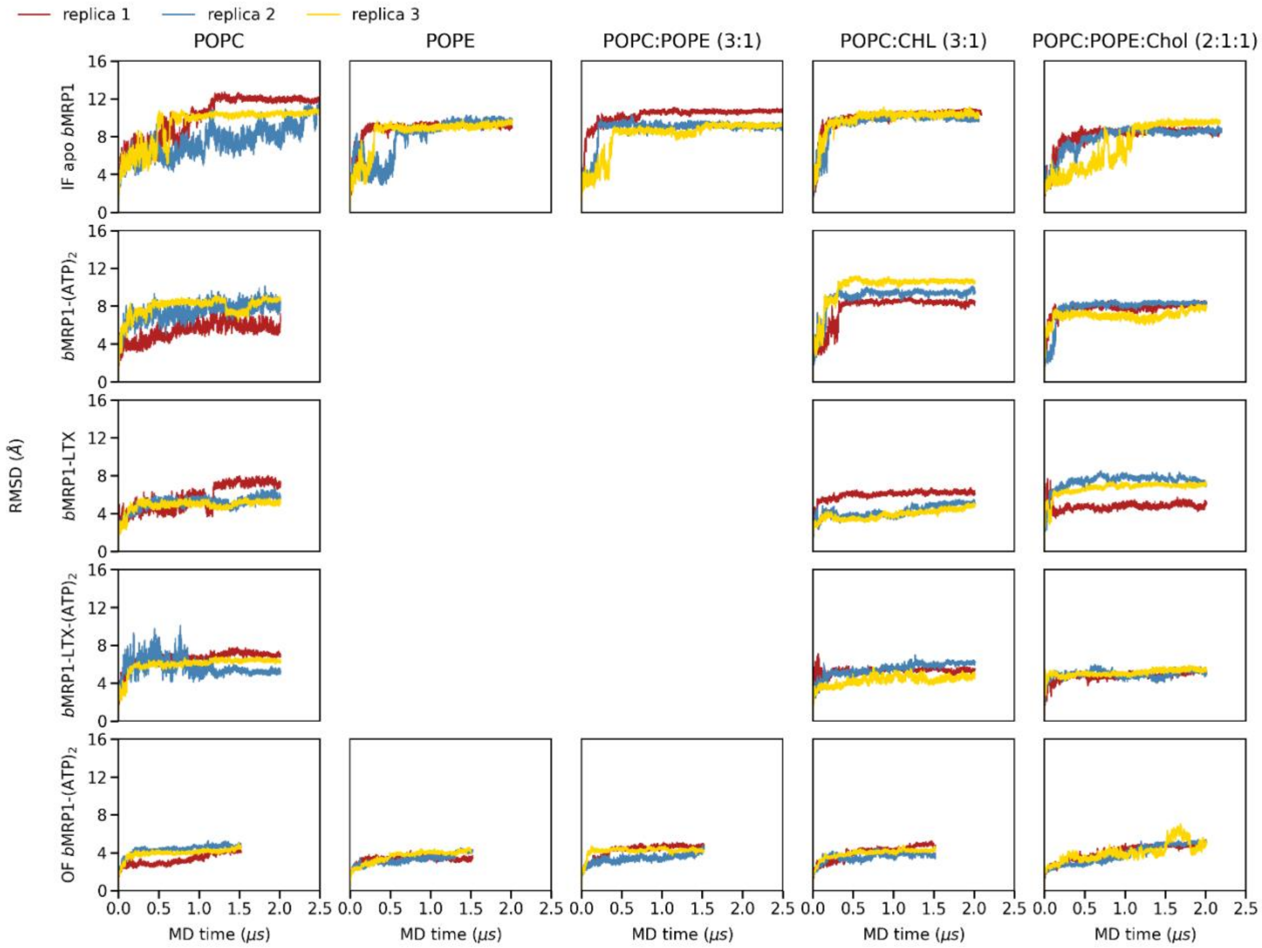

Supplementary Figure 40. Root-mean-square deviation (RMSD) along MD simulations calculated for all systems and for all different membrane compositions. Replica 1, 2 and 3 are coloured red, blue and yellow, respectively.

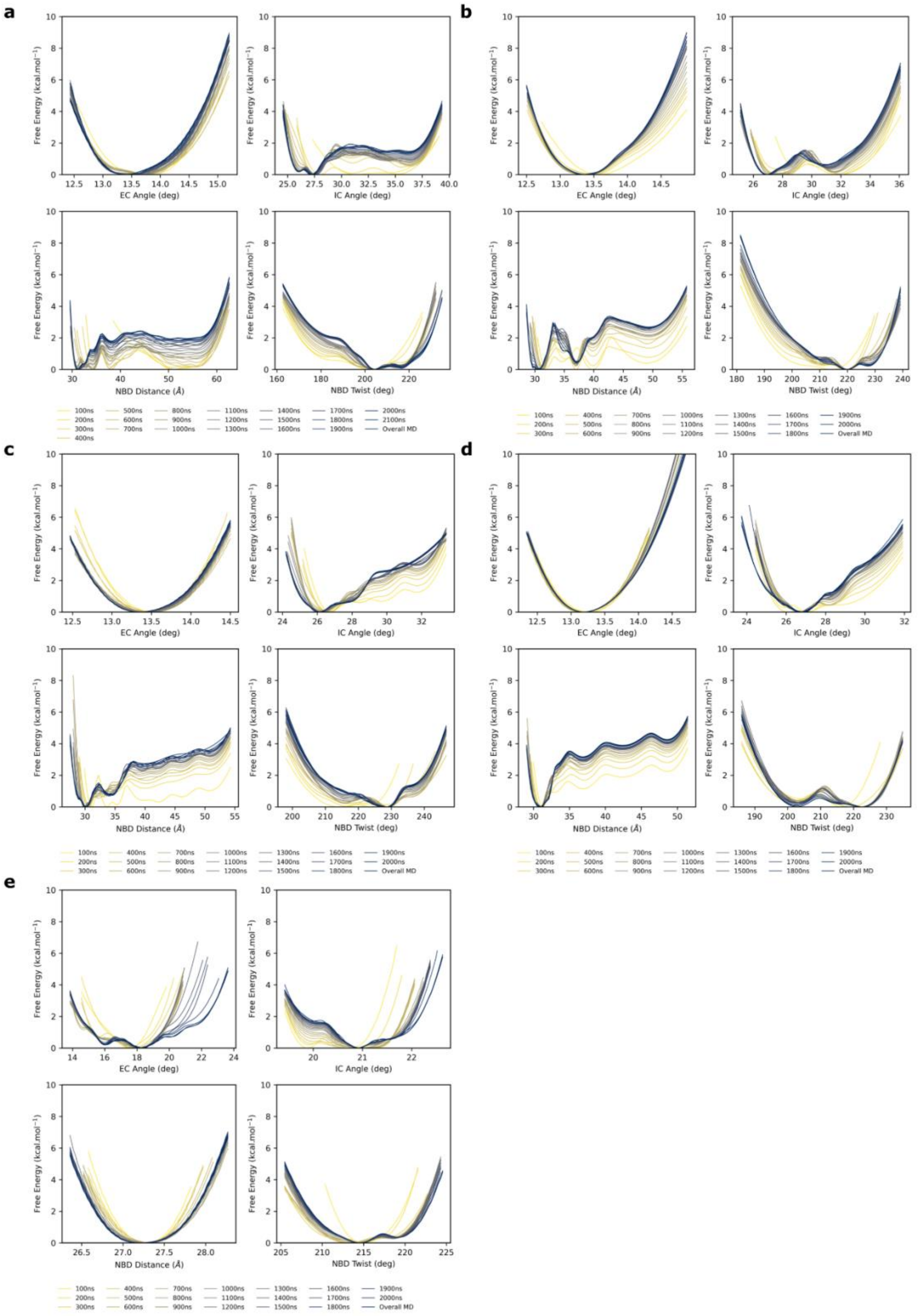

Supplementary Figure 41. Convergence of InfleCS calculations in POPC:POPE:Chol (2:1:1). **a)** IF apo *b*MRP1, **b)** *b*MRP1-$(ATP)_2$, **c)** *b*MRP1-LTX, **d)** *b*MRP1-LTX-$(ATP)_2$ and **e)** OF *b*MRP1-$(ATP)_2$.

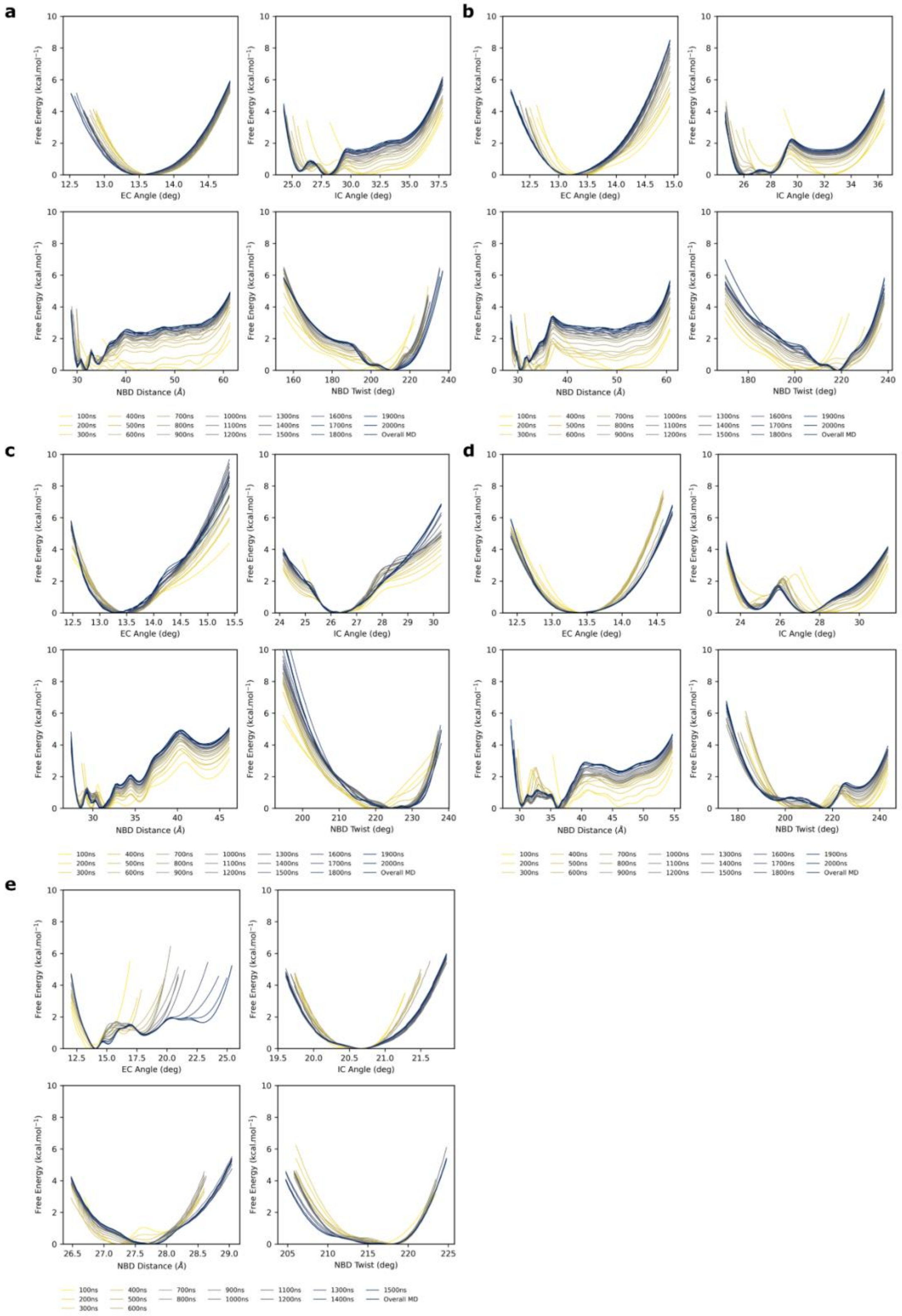

Supplementary Figure 42. Convergence of InfleCS calculations in POPC:Chol (3:1). a) IF apo *b*MRP1, **b)** *b*MRP1-(ATP)$_2$, **c)** *b*MRP1-LTX, **d)** *b*MRP1-LTX-(ATP)$_2$ and **e)** OF *b*MRP1-(ATP)$_2$.

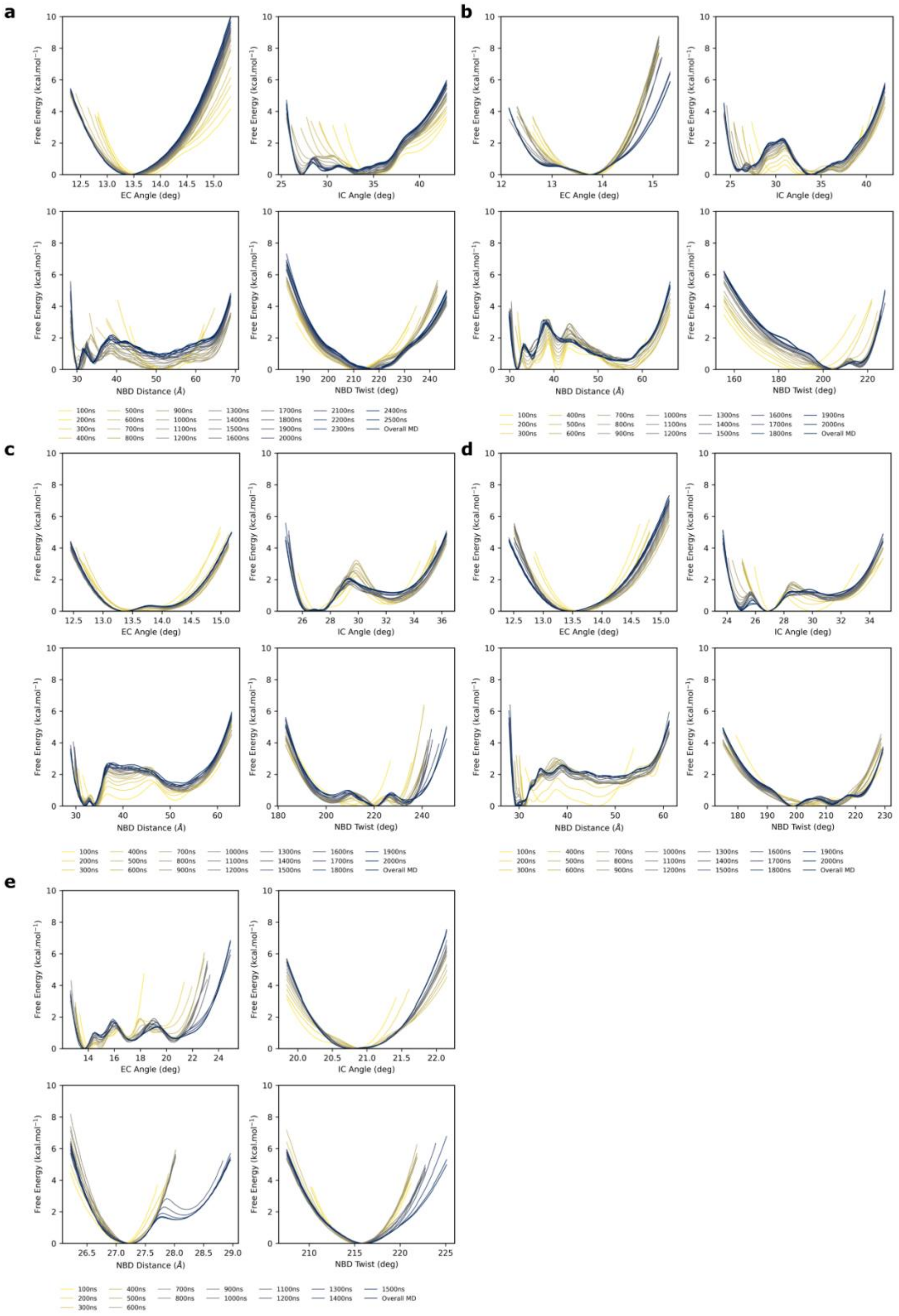

Supplementary Figure 43. Convergence of InfleCS calculations in pure POPC. a) IF apo *b*MRP1, **b)** *b*MRP1-$(ATP)_2$, **c)** *b*MRP1-LTX, **d)** *b*MRP1-LTX-$(ATP)_2$ and **e)** OF *b*MRP1-$(ATP)_2$.

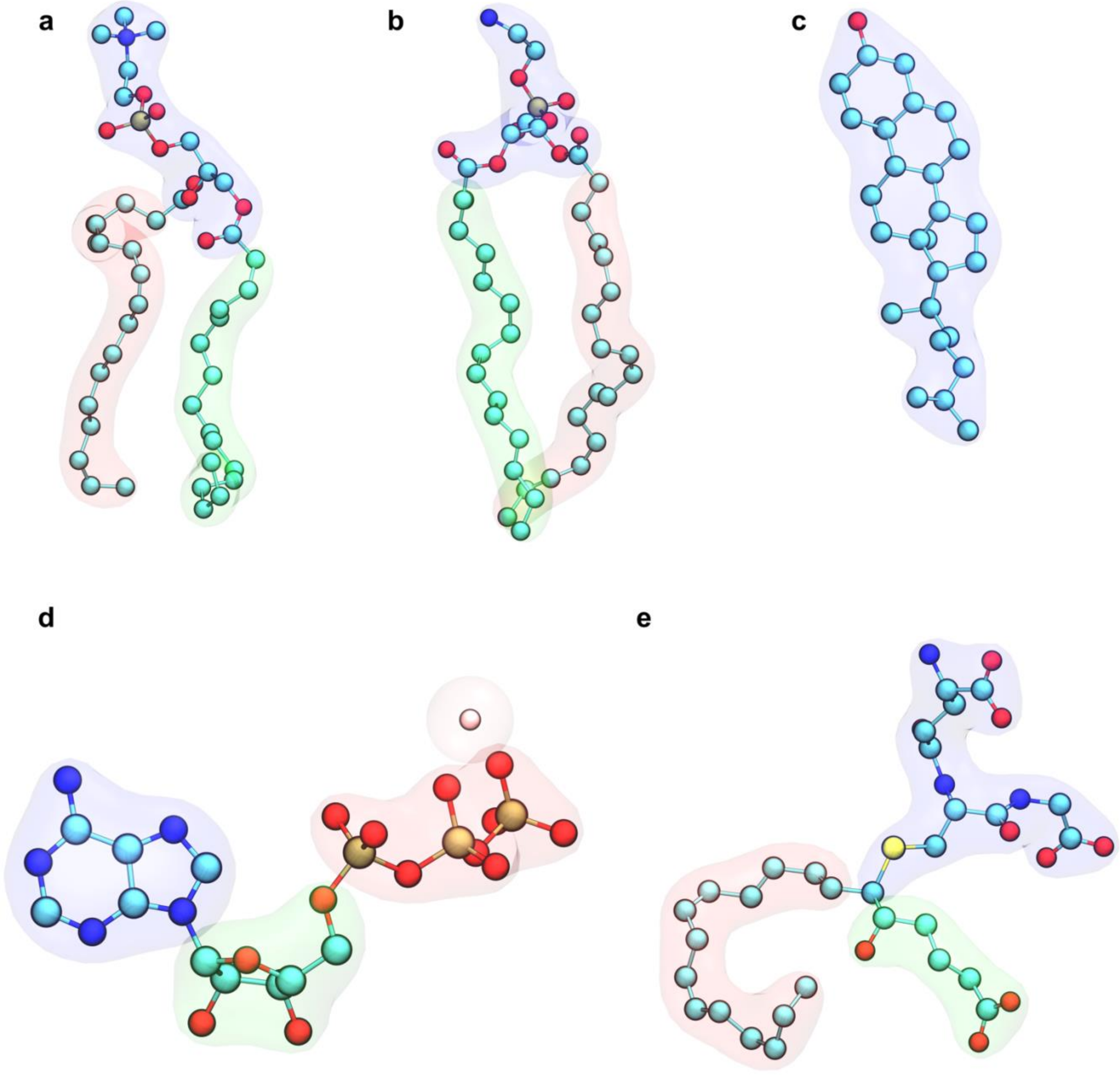

Supplementary Figure 44. Atom selections for co-factor nodes for allosteric pathway calculations. Each cofactor as split into fragments (depicted in blue, green, red and pink shades, accordingly) that were used to defined nodes for allosteric pathway calculations. PC and PE lipids were split into three nodes corresponding to polar head and lipid tails. Cholesterol and Mg^{2+} were considered as single node each. ATP molecule was split into three fragments (purine and sugar moieties as well as phosphate tail). LTX was split into three fragments (namely, glutathione moiety, aliphatic chain and 6-ketohexanoate moiety).

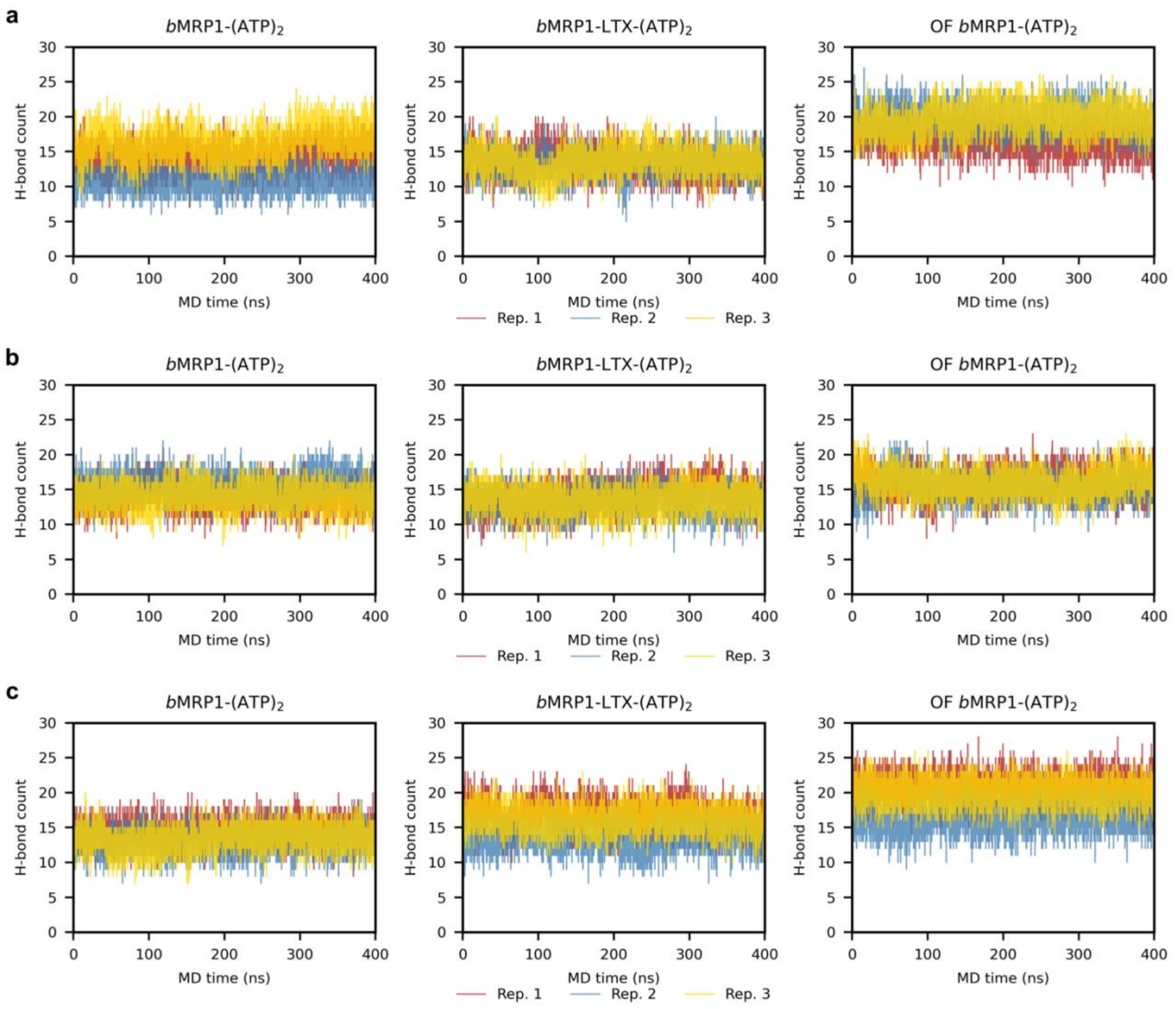

Supplementary Figure 45. Number of H-bonds calculated between nucleotides and *b*MRP1 protein over MD simulations. H-bond interactions were counted between nucleotides and protein residues over MD simulations performed on *b*MRP1-(ATP)$_2$, *b*MRP1-LTX-(ATP)$_2$ and OF *b*MRP1-(ATP)$_2$ systems embedded in **a)** POPC:POPE:Chol (2:1:1), **b)** POPC:Chol (3:1) and **c)** POPC. Distance and angle cutoffs were set 3.5 Å and 120°, respectively.

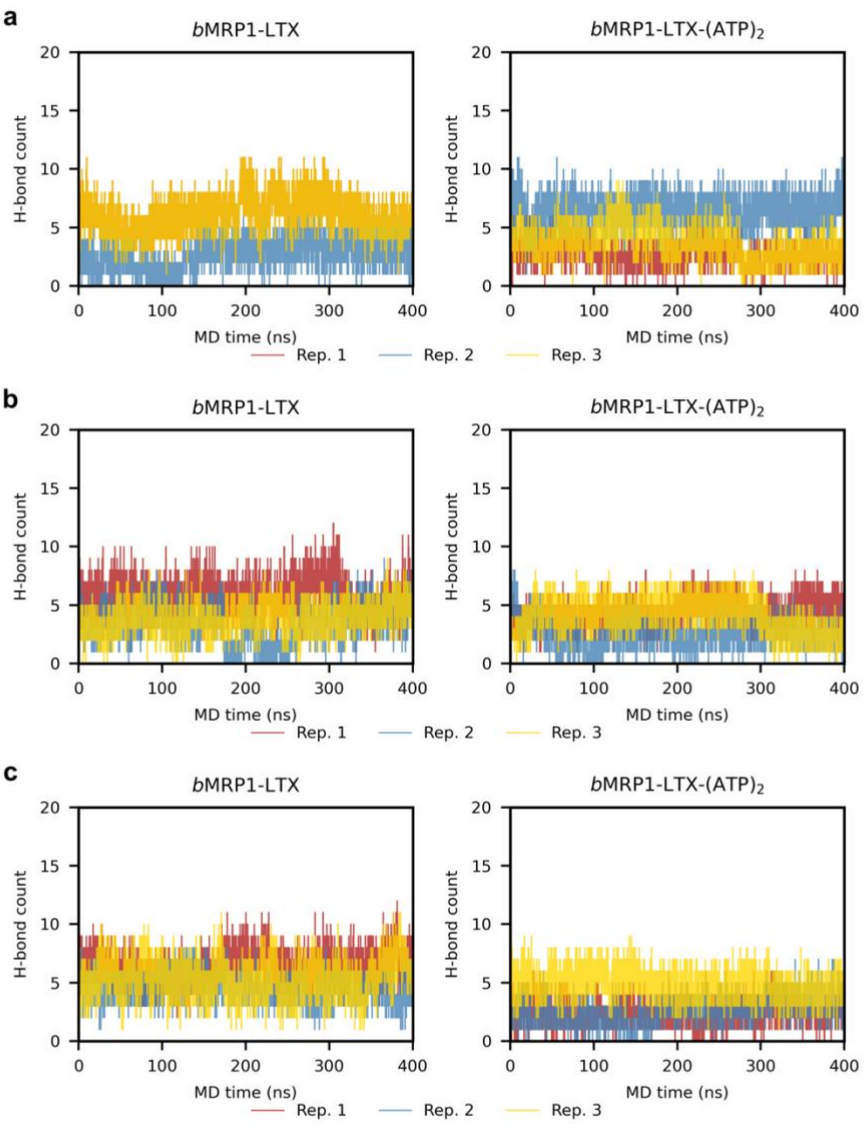

Supplementary Figure 46. Number of H-bonds calculated between LTX and *b*MRP1 protein over MD simulations. H-bond interactions were counted between LTX and proteins over MD simulations performed on *b*MRP1-LTX and *b*MRP1-LTX-$(ATP)_2$ systems embedded in **a)** POPC:POPE:Chol (2:1:1), **b)** POPC:Chol (3:1) and **c)** POPC. Distance and angle cutoffs were set 3.5 Å and 120°, respectively.

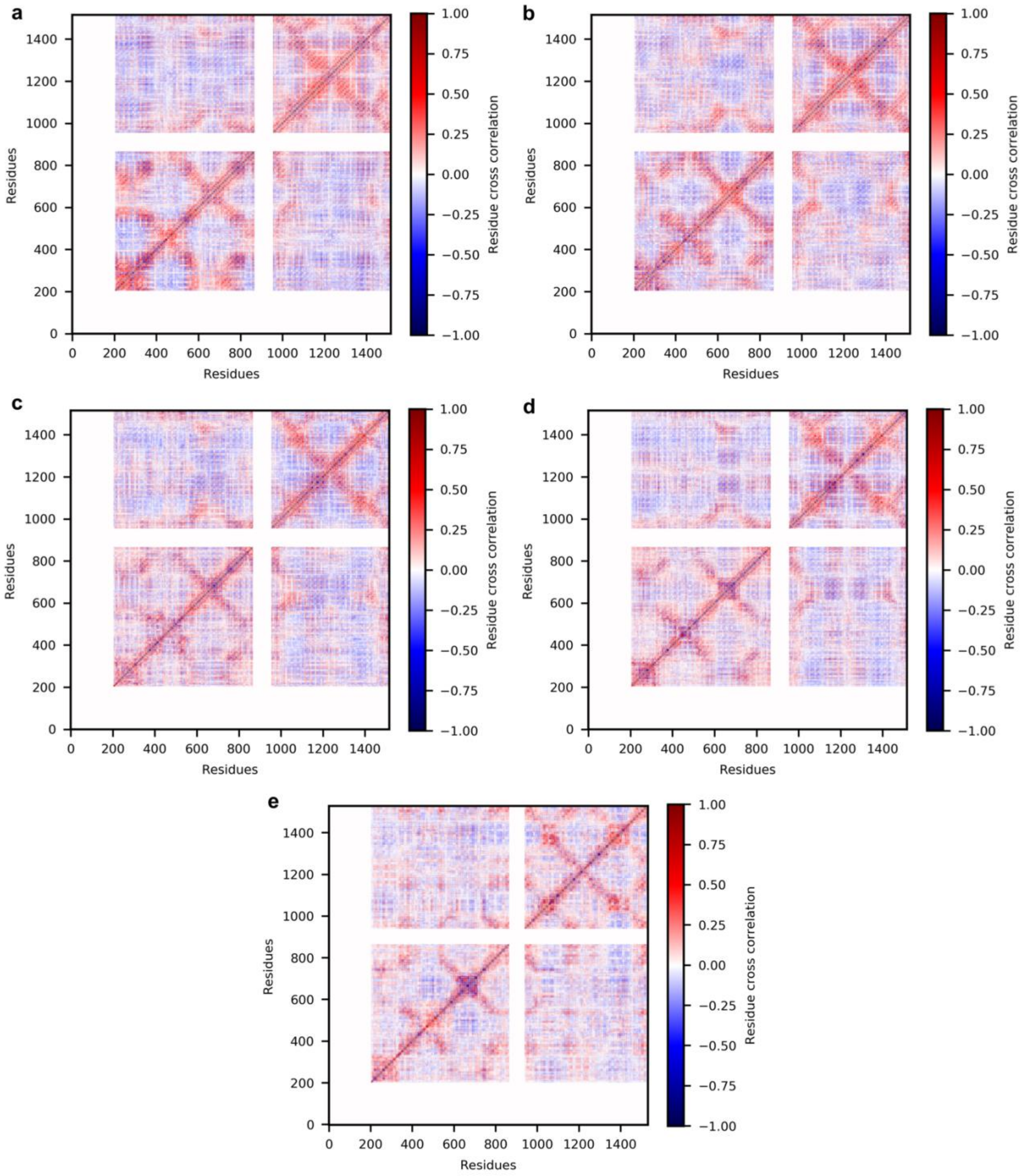

Supplementary Figure 47. Overall dynamic cross-correlation matrices from MD simulations performed in POPC. a) IF apo *b*MRP1, **b)** *b*MRP1-$(ATP)_2$, **c)** *b*MRP1-LTX, **d)** *b*MRP1-LTX-$(ATP)_2$ and **e)** OF *b*MRP1-$(ATP)_2$.

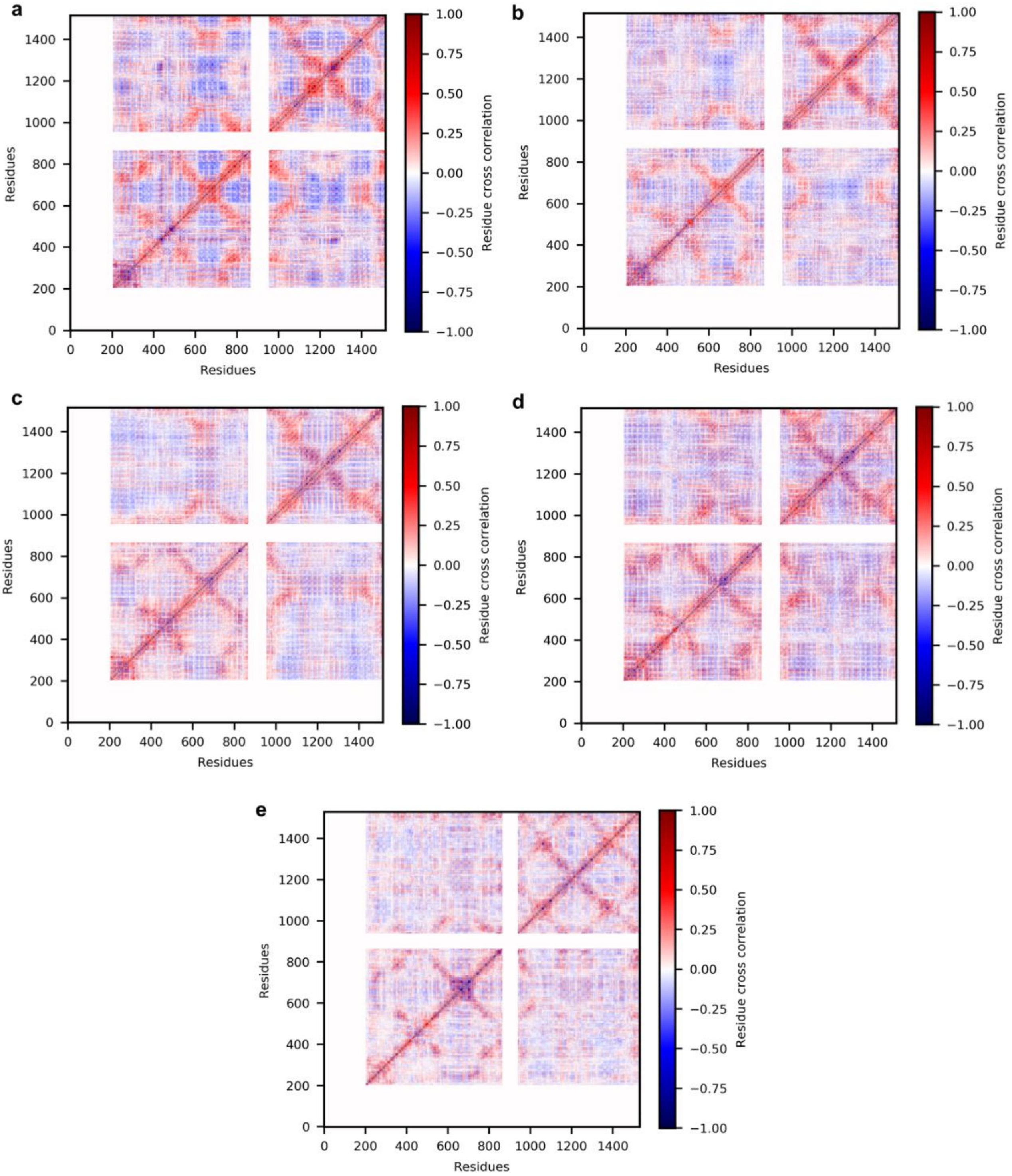

Supplementary Figure 48. Overall dynamic cross-correlation matrices from MD simulations performed in POPC:Chol (3:1). a) IF apo *b*MRP1, **b)** *b*MRP1-(ATP)$_2$, **c)** *b*MRP1-LTX, **d)** *b*MRP1-LTX-(ATP)$_2$ and **e)** OF *b*MRP1-(ATP)$_2$.

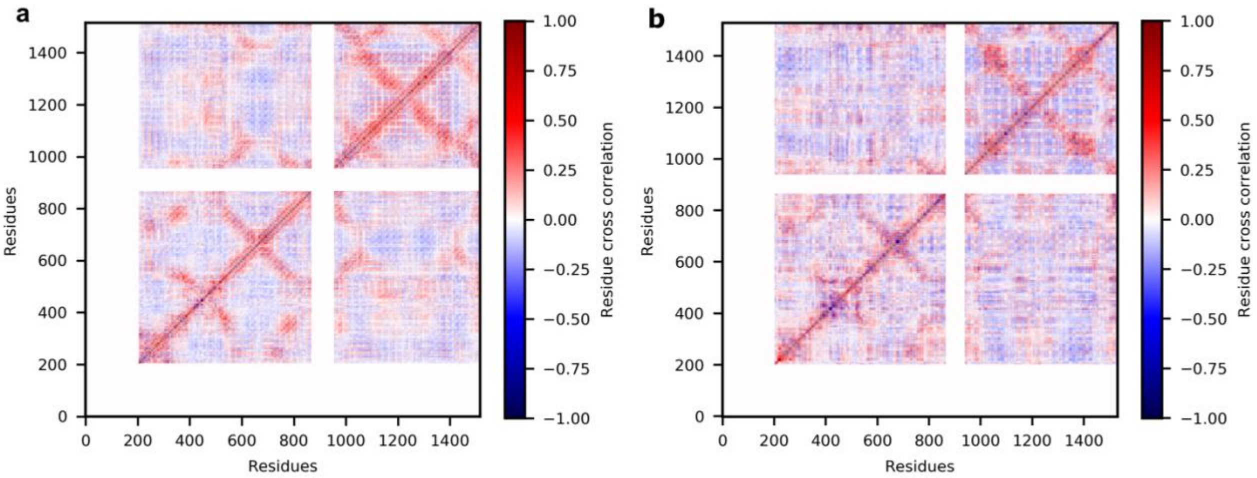

Supplementary Figure 49. Overall dynamic cross-correlation matrices from MD simulations performed in POPC:POPE (3:1). a) IF apo *b*MRP1 and **b)** OF *b*MRP1-$(ATP)_2$.

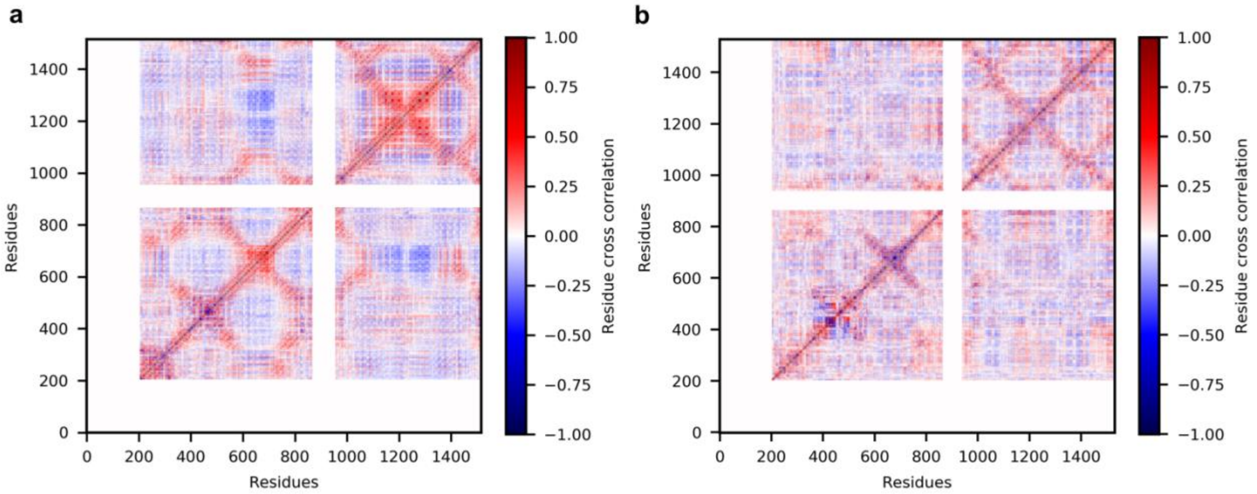

Supplementary Figure 50. Overall dynamic cross-correlation matrices from MD simulations performed in POPE. a) IF apo *b*MRP1 and **b)** OF *b*MRP1-$(ATP)_2$.

Supplementary Movies

Supplementary Movie 1. First principal component obtained from MD simulations performed on IF apo *b*MRP1 embedded in POPC:POPE:Chol (2:1:1). PCA were performed considering only the "so-called" ABC core, *i.e.*, TMHs and NBDs.

Supplementary Movie 2. First principal component obtained from MD simulations performed on IF *b*MRP1-(ATP)$_2$ embedded in POPC:POPE:Chol (2:1:1). PCA were performed considering only the "so-called" ABC core, *i.e.*, TMHs and NBDs.

Supplementary Movie 3. First principal component obtained from MD simulations performed on IF *b*MRP1-LTX embedded in POPC:POPE:Chol (2:1:1). PCA were performed considering only the "so-called" ABC core, *i.e.*, TMHs and NBDs.

Supplementary Movie 4. First principal component obtained from MD simulations performed on IF *b*MRP1-LTX-(ATP)$_2$ embedded in POPC:POPE:Chol (2:1:1). PCA were performed considering only the "so-called" ABC core, *i.e.*, TMHs and NBDs.

Supplementary Movie 5. First principal component obtained from MD simulations performed on OF *b*MRP1-(ATP)$_2$ embedded in POPC:POPE:Chol (2:1:1). PCA were performed considering only the "so-called" ABC core, *i.e.*, TMHs and NBDs.
