# Supplementary material for: On the interplay between lipids and asymmetric dynamics of an NBS degenerate ABC transporter
Source: Commun Biol. 2023 Feb 3;6:149. doi: 10.1038/s42003-023-04537-3 (PMC9898250; doi:10.1038/s42003-023-04537-3)
Supplement: Supplementary file 3 — Reporting Summary [file 42003_2023_4537_MOESM3_ESM.pdf]

nature portfolio

Corresponding author(s): Florent Di Meo
florent.di-meo@inserm.fr

Last updated by author(s): Jan 20, 2023

Reporting Summary

Nature Portfolio wishes to improve the reproducibility of the work that we publish. This form provides structure for consistency and transparency in reporting. For further information on Nature Portfolio policies, see our Editorial Policies and the Editorial Policy Checklist.

Statistics

For all statistical analyses, confirm that the following items are present in the figure legend, table legend, main text, or Methods section.

n/a	Confirmed	
☐	☒	The exact sample size (*n*) for each experimental group/condition, given as a discrete number and unit of measurement
☒	☐	A statement on whether measurements were taken from distinct samples or whether the same sample was measured repeatedly
☒	☐	The statistical test(s) used AND whether they are one- or two-sided<br>*Only common tests should be described solely by name; describe more complex techniques in the Methods section.*
☒	☐	A description of all covariates tested
☒	☐	A description of any assumptions or corrections, such as tests of normality and adjustment for multiple comparisons
☐	☒	A full description of the statistical parameters including central tendency (e.g. means) or other basic estimates (e.g. regression coefficient) AND variation (e.g. standard deviation) or associated estimates of uncertainty (e.g. confidence intervals)
☒	☐	For null hypothesis testing, the test statistic (e.g. *F*, *t*, *r*) with confidence intervals, effect sizes, degrees of freedom and *P* value noted<br>*Give P values as exact values whenever suitable.*
☒	☐	For Bayesian analysis, information on the choice of priors and Markov chain Monte Carlo settings
☒	☐	For hierarchical and complex designs, identification of the appropriate level for tests and full reporting of outcomes
☒	☐	Estimates of effect sizes (e.g. Cohen's *d*, Pearson's *r*), indicating how they were calculated

Our web collection on statistics for biologists contains articles on many of the points above.

Software and code

Policy information about availability of computer code

Data collection	Systems were prepared using the online CHARMM-GUI input generator, as well as the AmberTools suite (Version 18). Ligand parameterization was conducted using the R.E.D. vIII server.<br>Molecular dynamics simulations were performed using the Amber18&20 software (CPU and GPU version of the PMEMD binary).
Data analysis	Analyses were conducted using (i) cpptraj package in the AmberTools 21 package, (ii) VMD versions 1.9.3 and 1.9.4 (alpha). Allosteric pathway analyses and InfleCS were performed using Lucie Delemotte's lab gitlab (https://github.com/delemottelab). Membrane Free energy deformations were calculated using the CTMDapp software (http://memprotein.org/new-continuum-molecular-dynamics-ctmd-software-made-available-by-computational-modeling-core)

For manuscripts utilizing custom algorithms or software that are central to the research but not yet described in published literature, software must be made available to editors and reviewers. We strongly encourage code deposition in a community repository (e.g. GitHub). See the Nature Portfolio guidelines for submitting code & software for further information.

Data

Policy information about availability of data

All manuscripts must include a data availability statement. This statement should provide the following information, where applicable:

- Accession codes, unique identifiers, or web links for publicly available datasets
- A description of any restrictions on data availability
- For clinical datasets or third party data, please ensure that the statement adheres to our policy

Data set generated from MD simulations conducted in this work are available from the authors on reasonable requests. Source data underlying results and plot presented in the main figures, MD inputs as well as initial and final coordinate configurations for each state and lipid model can be downloaded at the following Zenodo access link: https://doi.org/10.5281/zenodo.7541178.

Human research participants

Policy information about studies involving human research participants and Sex and Gender in Research.

Reporting on sex and gender	Not applicable
Population characteristics	Not applicable
Recruitment	Not applicable
Ethics oversight	Not applicable

Note that full information on the approval of the study protocol must also be provided in the manuscript.

Field-specific reporting

Please select the one below that is the best fit for your research. If you are not sure, read the appropriate sections before making your selection.

☒ Life sciences ☐ Behavioural & social sciences ☐ Ecological, evolutionary & environmental sciences

For a reference copy of the document with all sections, see nature.com/documents/nr-reporting-summary-flat.pdf

Life sciences study design

All studies must disclose on these points even when the disclosure is negative.

Sample size	For each MD simulation, trajectory lengths were determined by monitoring overall structural RMSD and time-dependent evolutions of ABC structural parameters. MD simulations were performed for 1500 ns up to 2510 ns.
Data exclusions	Equilibration part of MD simulations (pictured by RMSD plots) were not systematically considered (except for time-dependent monitoring of ABC structural parameters displayed in Figure 1). Structural analyses must be conducted only when the whole system has reached the local subspace minimum.
Replication	For each system (i.e., conformations, bound-states and lipid bilayers), three replicas were performed, by performing three distinct thermalization during MD equilibration steps. Replicas were conducted in order to account the inherent structural and dynamic variabilities of molecular dynamics simulations.
Randomization	Seeds for initial velocities regarding MD thermalization steps were randomly set up (Amber manual, ig = -1 keywords).
Blinding	Not relevant to MD simulation trajectories since all data were exhaustively considered during MD analyses.

Reporting for specific materials, systems and methods

We require information from authors about some types of materials, experimental systems and methods used in many studies. Here, indicate whether each material, system or method listed is relevant to your study. If you are not sure if a list item applies to your research, read the appropriate section before selecting a response.

Materials & experimental systems

n/a	Involved in the study
☒	☐ Antibodies
☒	☐ Eukaryotic cell lines
☒	☐ Palaeontology and archaeology
☒	☐ Animals and other organisms
☒	☐ Clinical data
☒	☐ Dual use research of concern

Methods

n/a	Involved in the study
☒	☐ ChIP-seq
☒	☐ Flow cytometry
☒	☐ MRI-based neuroimaging
